# Supplementary material for: Adolescent stress impairs postpartum social behavior via anterior insula-prelimbic pathway in mice
Source: Nat Commun. 2023 May 23;14:2975. doi: 10.1038/s41467-023-38799-6 (PMC10205810; doi:10.1038/s41467-023-38799-6)
Supplement: Supplementary file 1 — Supplementary Information [file 41467_2023_38799_MOESM1_ESM.pdf]

Supplementary Information for

Adolescent stress impairs postpartum social behavior via anterior insula-prelimbic pathway in mice

Kyohei Kin[1], Jose Francis-Oliveira[1], Shin-ichi Kano[1,2], Minae Niwa[1,2,3]*

1 Department of Psychiatry and Behavioral Neurobiology, University of Alabama at Birmingham Heersink School of Medicine, Birmingham, AL 35233, USA

2 Department of Neurobiology, University of Alabama at Birmingham Heersink School of Medicine, Birmingham, AL 35233, USA

3 Department of Biomedical Engineering, University of Alabama at Birmingham School of Engineering, Birmingham, AL 22908, USA

*Corresponding author: Minae Niwa, mniwa@uabmc.edu

This PDF file includes:

Supplementary Figs. 1 to 16

Supplementary Tables 1 to 3

Supplementary Fig. 1

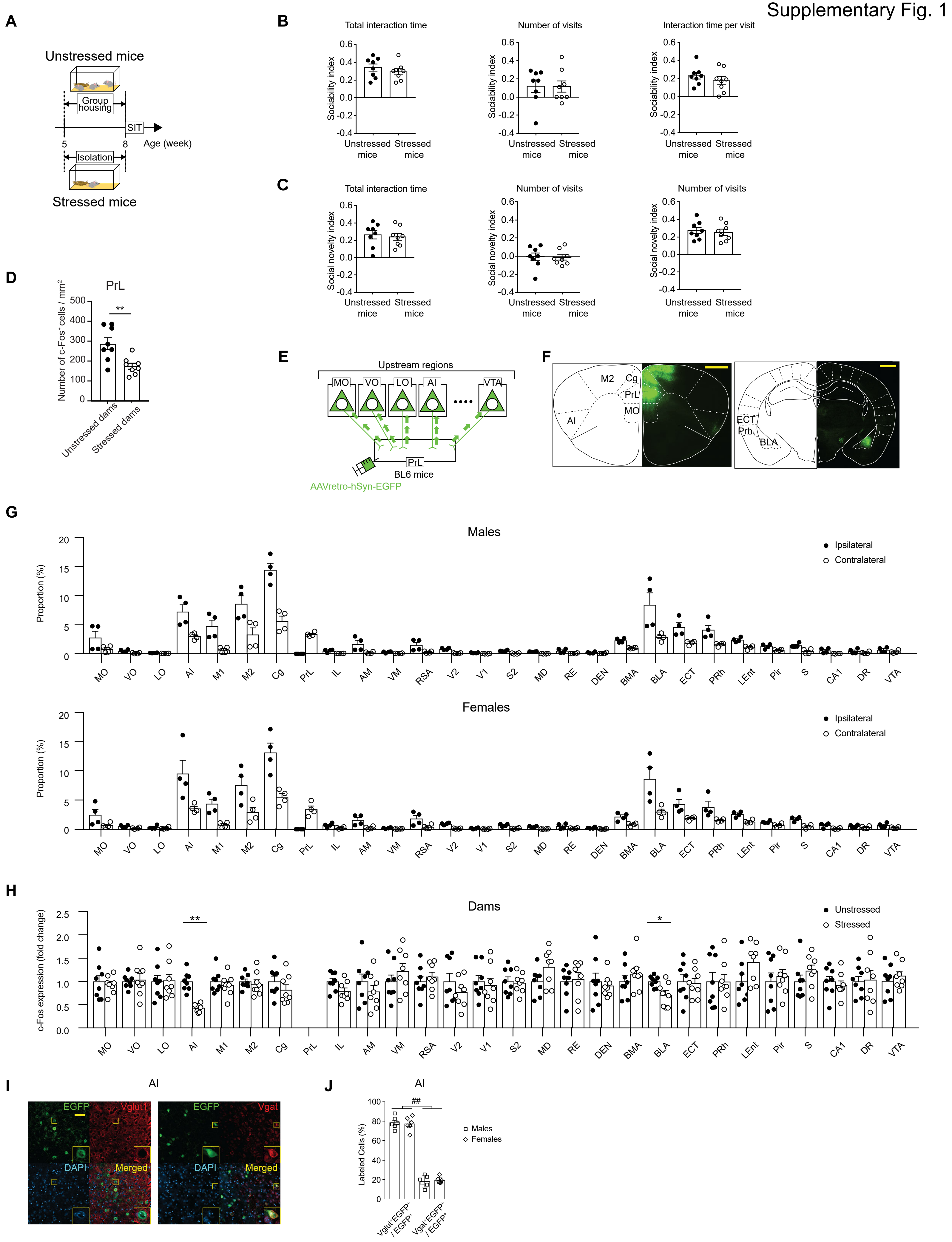

Supplementary Fig. 1. SILA led to reduced postpartum neuronal activity in PrL and AI after SN-trial. (**A**) Experimental timeline of the social interaction test (SIT) immediately after SILA. (**B** and **C**) No behavioral changes were observed during S- and SN-trials immediately after SILA (Student's *t* test). N = 8 mice. (**D**) c-Fos expression after the SN-trial was decreased in PrL of stressed dams in comparison to unstressed dams (Student's *t* test). N = 8 mice. (**E**) Scheme of retrograde tracing in BL6 mice injected with AAVretro-hSyn-EGFP into PrL. (**F**) Representative images showing labeled neurons in cingulate cortex (Cg), second motor cortex (M2), AI, and BLA. Scale bars, 1 mm. (**G**) Whole-brain quantification of projections to PrL, in male and female mice. For definitions of the abbreviations, see **Supplementary Table 1** (Multiple Paired Student's *t* test and Wilcoxon signed-rank test. Statistical significance not shown for clarity. See **Supplementary Table 3** for *p* values for each region analyzed). (**H**) c-Fos expression levels in dams upstream regions of PrL after SN-trials. c-Fos expression was decreased only in AI and basolateral amygdala (BLA) of stressed dams in comparison to unstressed dams. For definitions of the abbreviations, see **Supplementary Table 1** (Multiple Student's *t* test and Mann-Whitney U test. *p* values <0.10 are also indicated). N = 8 mice. (**I**) Representative images of $EGFP^+$ (green), $Vglut1^+$ or $Vgat^+$ (red), DAPI (blue), and colocalized cells in AI of mice injected with AAVretro-hSyn-EGFP into PrL. Scale bar, 50 µm. (**J**) AI-PrL pathway mainly consisted of $Vglut1^+$ neurons, not $Vgat^+$ neurons (two-way mixed ANOVA). N = 6 mice. All data are represented as mean ± SEM. For ANOVAs, * indicates statistical significance for *post hoc* Bonferroni comparisons, and # indicates statistical significance for the main effect. * = $p < 0.05$, ** = $p < 0.01$, ## = $p < 0.01$. See **Supplemental Table 3** for details on the statistical analyses.

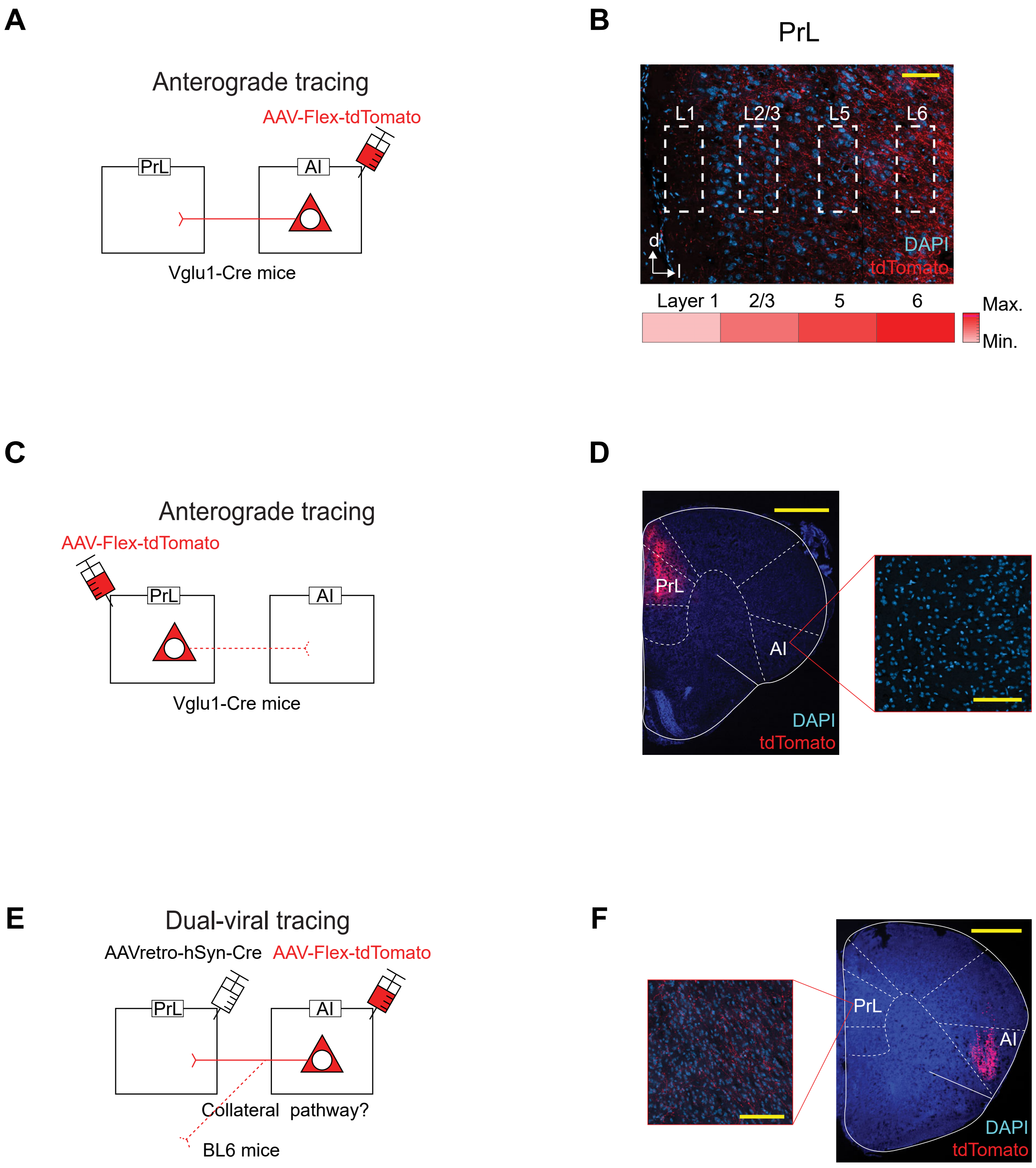
A
Anterograde tracing
AAV-Flex-tdTomato
PrL
AI
Vglu1-Cre mice
B
PrL
L1
L2/3
L5
L6
d
l
DAPI
tdTomato
Layer 1
2/3
5
6
Max.
Min.
C
Anterograde tracing
AAV-Flex-tdTomato
PrL
AI
Vglu1-Cre mice
D
PrL
AI
DAPI
tdTomato
E
Dual-viral tracing
AAVretro-hSyn-Cre
AAV-Flex-tdTomato
PrL
AI
Collateral pathway?
BL6 mice
F
PrL
AI
DAPI
tdTomato

Supplementary Fig. 2. AI-PrL pathway was a unidirectional pathway from AI to PrL with no collateral projections. (**A**) Scheme of anterograde tracing in Vglut1-Cre female mice injected with AAV-Flex-tdTomato into AI. (**B**) Representative image showing expression of tdTomato (red) at terminals of the AI-PrL pathway in PrL. Terminals of the AI-PrL pathway had the highest density in deep layers. Scale bar, 100 μm. (**C**) Scheme of anterograde tracing in Vglut1-Cre mice injected with AAV-Flex-tdTomato into PrL. (**D**) Representative images showing expression of tdTomato (red) in PrL, but not in AI. Scale bars, 1 mm and 100 μm. (**E**) Scheme of dual-virus tracing in BL6 female mice injected with AAV-Flex-tdTomato and AAVretro-hSyn-Cre into AI and PrL, respectively, to explore the collateral pathway of the AI-PrL pathway. (**F**) Representative images showing expression of tdTomato (red) in only AI and PrL. Scale bars, 100 μm and 1 mm.

Supplementary Fig. 3

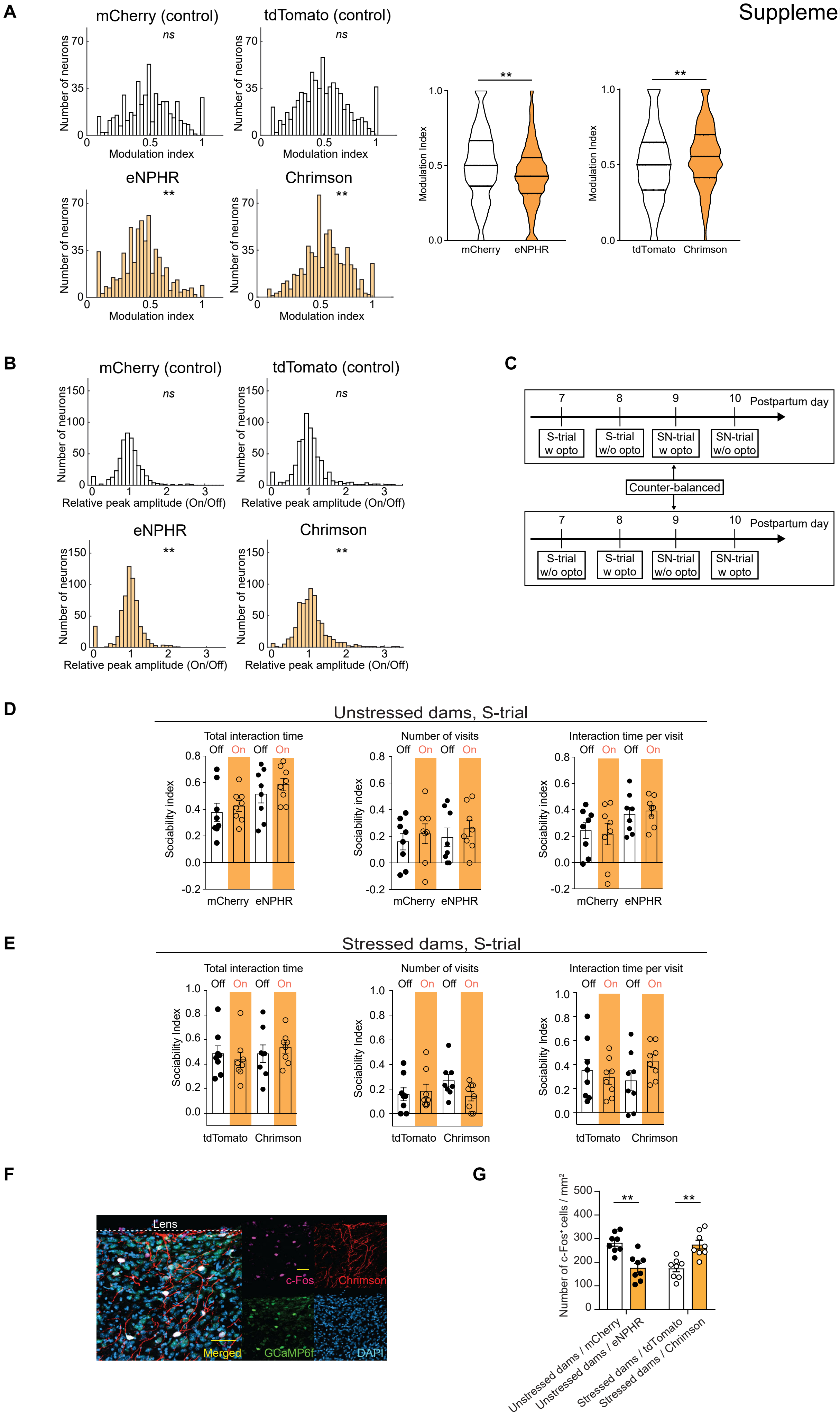

Supplementary Fig. 3. AI-PrL pathway did not affect sociability in the postpartum period. (**A**) Modulation index for PrL neurons showed inhibition or activation of calcium transients during optogenetic activation or suppression of the AI-PrL pathway, respectively (Wilcoxon signed-rank test for one sample, $r > 0.25$ indicating small to intermediate effect size). Comparison between control and opsin-expressing group of cells, showing the median ± interquartile range in a violin plot (Mann Whitney U test, $r > 0.10$ indicating small effect size). (**B**) Calcium transient peak amplitudes of PrL were increased or decreased by optogenetic activation or inhibition of AI-PrL pathway, respectively (Wilcoxon signed-rank test for one sample, $r > 0.1$ indicating small effect size). (**C**) Experimental timeline of SIT without and with optogenetic manipulation. (**D** and **E**) Optogenetic manipulation of the AI-PrL pathway did not affect sociability in both stressed and unstressed dams (two-way mixed ANOVA, Wilcoxon signed-rank test). N = 8 mice. (**F**) Representative images showing expression of GCaMP6f (green) underneath a GRIN lens, pathway from AI expressing Chrimson (red), and labeled c-Fos^{+} (far red) cells in PrL. Scale bars, 50 μm. (**G**) c-Fos expression in PrL was decreased by optogenetic inhibition of the AI-PrL pathway in unstressed dams, and increased by optogenetic activation of the AI-PrL pathway in stressed dams (two-way ANOVA, $p < 0.01$ for interaction). N = 8 mice. Except for the modulation indexes in **Supplementary Figure 3A**, data are represented as mean ± SEM. For ANOVAs, * indicates statistical significance for *post hoc* Bonferroni comparisons. * = $p < 0.05$, ** = $p < 0.01$. *ns* = non-significant ($p > 0.05$). See **Supplemental Table 3** for details on the statistical analyses.

Supplementary Fig. 4

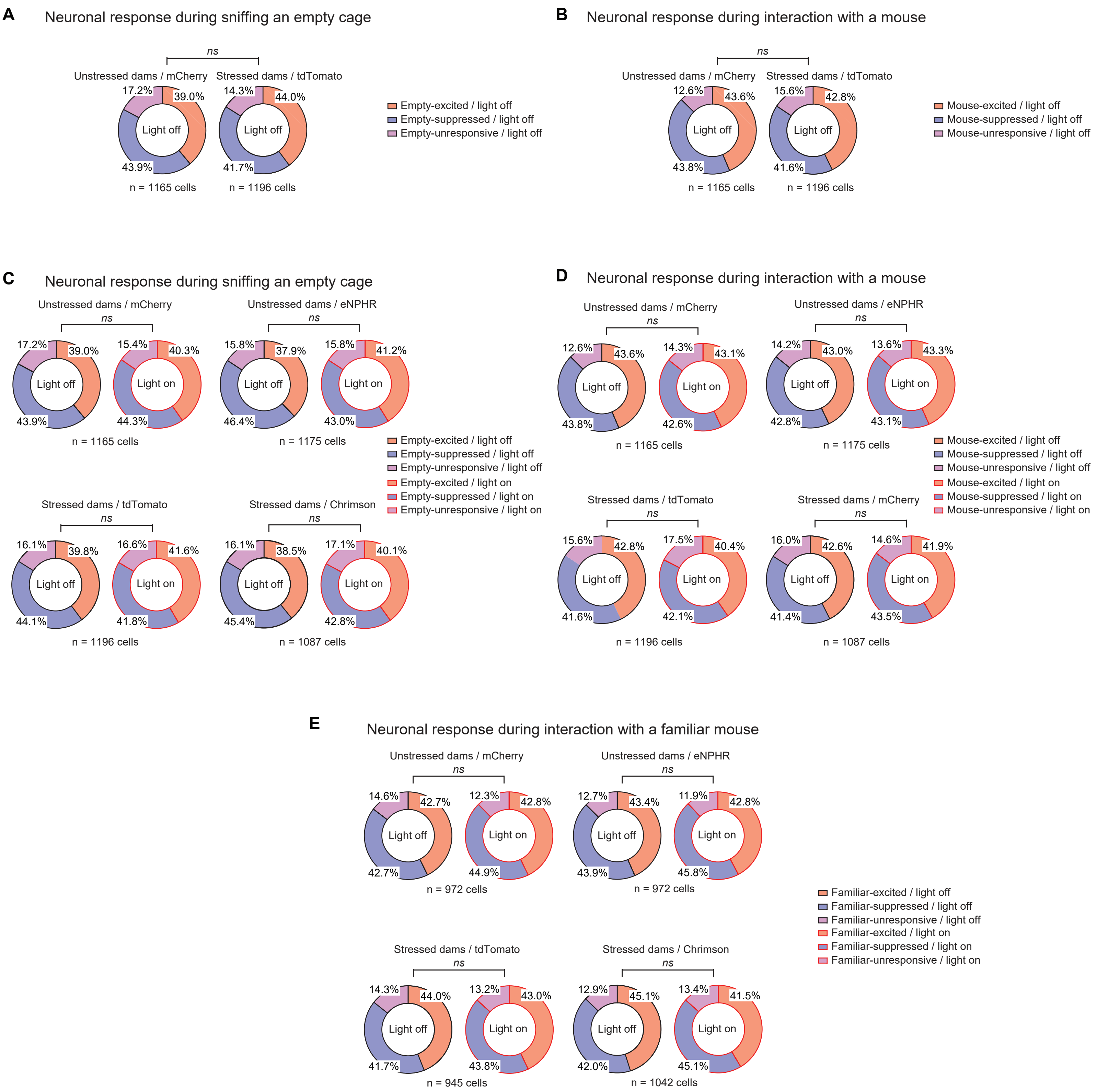

Supplementary Fig. 4. AI-PrL pathway did not affect neuronal activity in PrL and sociability in the postpartum period. (**A** and **B**) PrL activity during S-trials did not show any differences between unstressed and stressed dams expressing control viruses (Chi-squared test). (**C** and **D**) Optogenetic manipulation of the AI-PrL pathway did not affect PrL activity patterns during S-trials in both stressed and unstressed dams (Chi-squared test). (**E**) Optogenetic manipulation of the AI-PrL pathway in both stressed and unstressed dams did not affect PrL activity patterns during interaction with familiar mice (Chi-squared test). *ns* = non-significant ($p > 0.05$). See **Supplemental Table 3** for details on the statistical analyses.

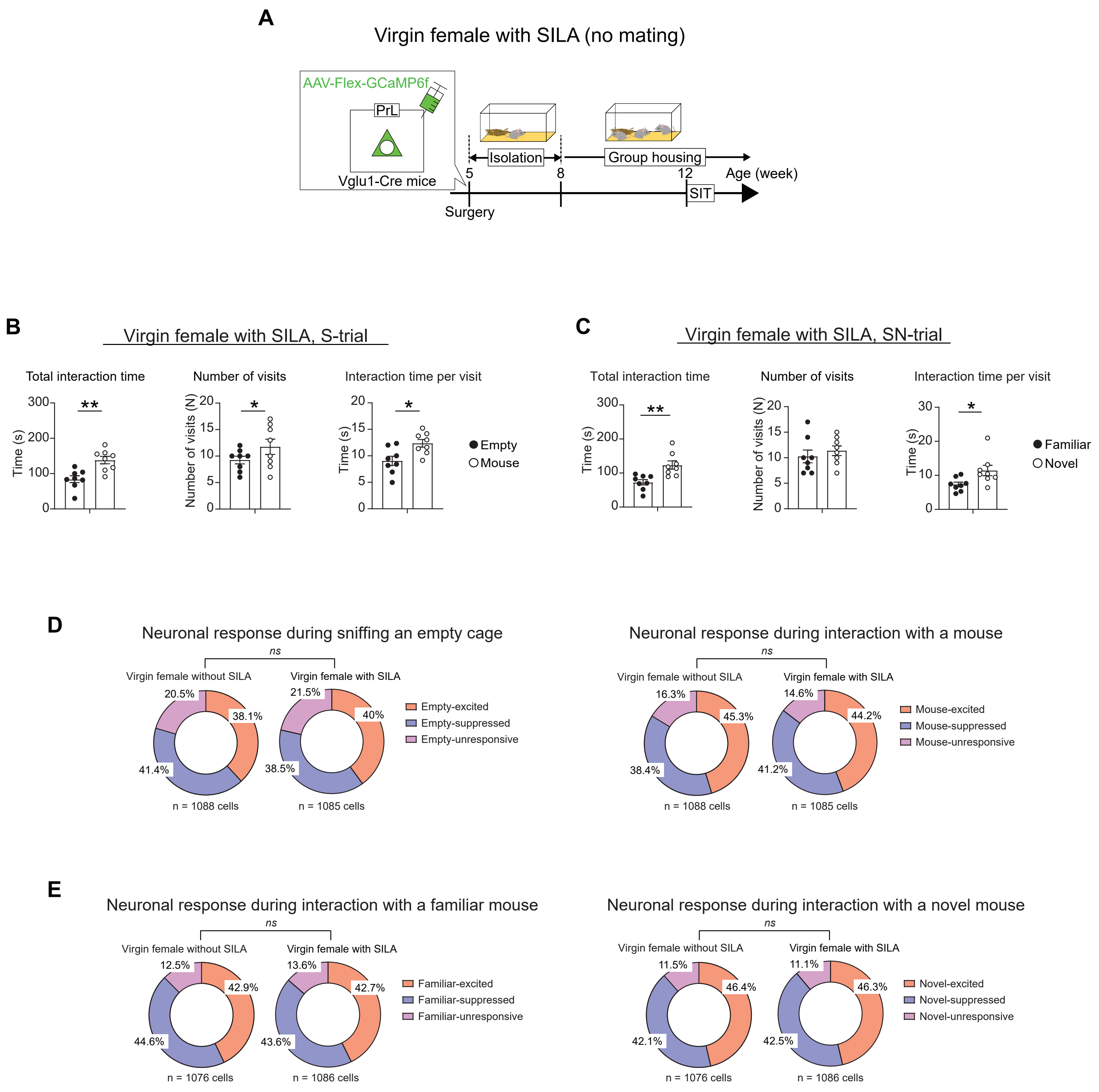
A
Virgin female with SILA (no mating)
AAV-Flex-GCaMP6f
PrL
Vglu1-Cre mice
Isolation
Group housing
5
8
12
Age (week)
SIT
Surgery
B
Virgin female with SILA, S-trial
Total interaction time
Number of visits
Interaction time per visit
Time (s)
Number of visits (N)
**
*
*
Empty
Mouse
C
Virgin female with SILA, SN-trial
Total interaction time
Number of visits
Interaction time per visit
**
*
Familiar
Novel
D
Neuronal response during sniffing an empty cage
ns
Virgin female without SILA
Virgin female with SILA
20.5%
38.1%
41.4%
21.5%
40%
38.5%
n = 1088 cells
n = 1085 cells
Empty-excited
Empty-suppressed
Empty-unresponsive
Neuronal response during interaction with a mouse
16.3%
45.3%
38.4%
14.6%
44.2%
41.2%
Mouse-excited
Mouse-suppressed
Mouse-unresponsive
E
Neuronal response during interaction with a familiar mouse
12.5%
42.9%
44.6%
13.6%
42.7%
43.6%
n = 1076 cells
n = 1086 cells
Familiar-excited
Familiar-suppressed
Familiar-unresponsive
Neuronal response during interaction with a novel mouse
11.5%
46.4%
42.1%
11.1%
46.3%
42.5%
Novel-excited
Novel-suppressed
Novel-unresponsive

Supplementary Fig. 5. SILA alone did not affect PrL activity patterns during SIT. (**A**) Scheme of experimental timeline and strategy for *in vivo* microendoscopic calcium imaging in PrL. (**B** and **C**) In virgin females, SILA alone did not cause behavioral changes both in S- and SN-trials, in which they showed strong sociability and social novelty preference (Paired Student's t test, Cohen's D > 0.8 for all significant p values, and Wilcoxon signed rank test, $r > 0.8$). N = 8 mice. (**D** and **E**) In virgin females, SILA alone did not affect PrL activity patterns during S- and SN-trials (Chi-squared test). * $p < 0.05$, ** $p < 0.01$. *ns* = non-significant ($p > 0.05$). All data are represented as mean ± SEM. See **Supplemental Table 3** for details on the statistical analyses.

Supplementary Fig. 6

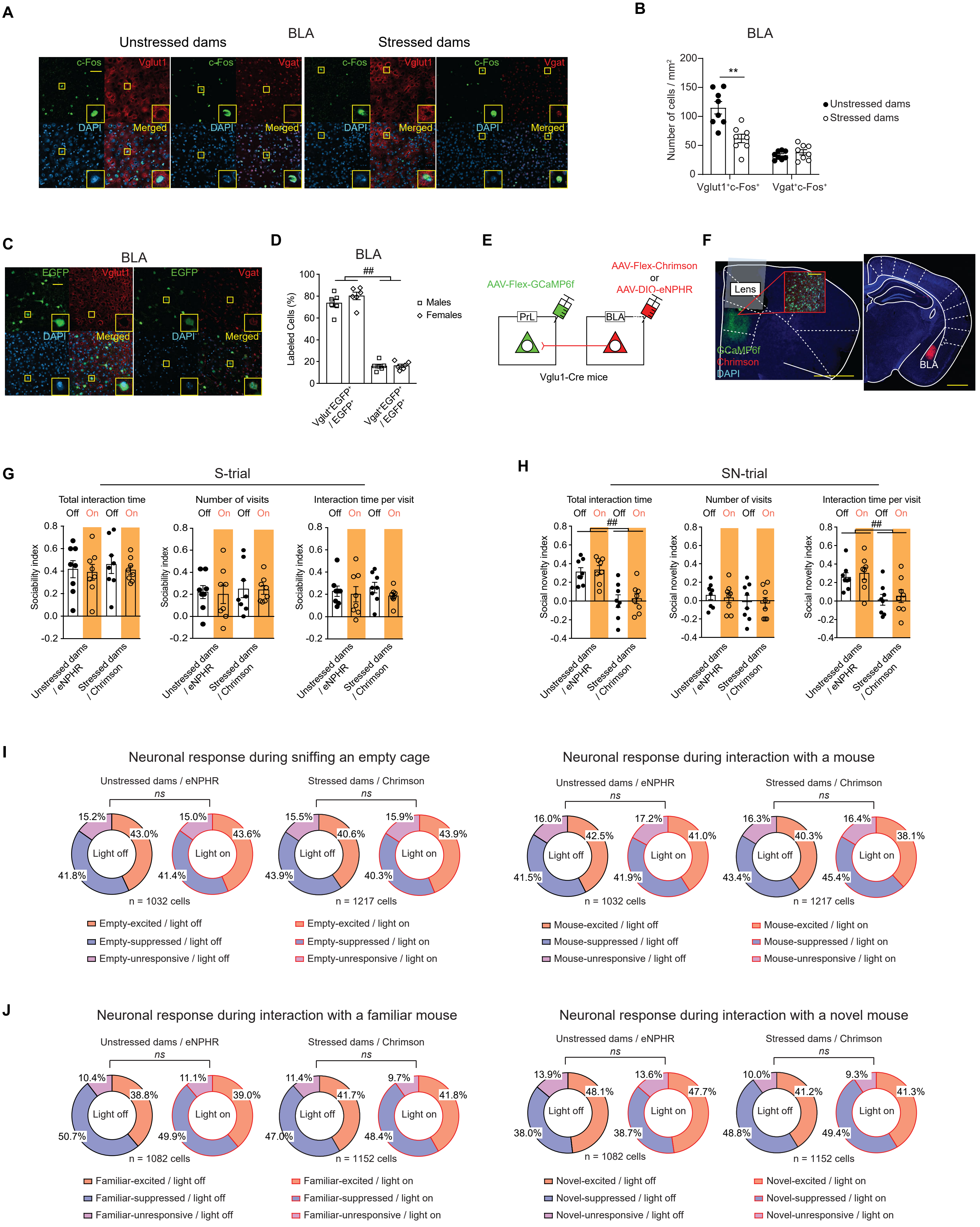

Supplementary Fig. 6. BLA-PrL pathway did not affect PrL activity patterns and social behavior in the postpartum period. (**A**) Representative images of c-Fos$^+$ (green), Vglut1$^+$ or Vgat$^+$ (red), DAPI (blue), and colocalized cells in BLA of unstressed or stressed dams. Scale bar, 50 µm. (**B**) Stressed dams showed decreased number of Vglut1$^+$c-Fos$^+$ cells, but not Vgat$^+$c-Fos$^+$, in BLA after SN-trials (Student's *t* test, Welch *t* test). N = 8 mice. (**C**) Representative images of EGFP$^+$ (green), Vglut1$^+$ or Vgat$^+$ (red), DAPI (blue), and colocalized cells in BLA from mice injected with AAVretro-hSyn-EGFP into PrL. Scale bar, 50 µm. (**D**) BLA-PrL pathway mainly consisted of Vglut1$^+$ neurons, but not Vgat$^+$ neurons (two-way ANOVA, main effect partial $\eta^2 = 0.98$). N = 6 mice. (**E**) Strategy of *in vivo* microendoscopic calcium imaging of PrL through optogenetic manipulation of the BLA-PrL pathway. (**F**) Representative images showing expressions of Chrimson (red) in the BLA-PrL pathway and GCaMP6f (green) underneath a GRIN lens. Scale bars, 1 mm and 100 µm. (**G** and **H**) Optogenetic manipulation of the BLA-PrL pathway did not affect social behavior in S- and SN-trials (two-way mixed ANOVA, Wilcoxon signed-rank test). N = 8 mice. (**I** and **J**) Optogenetic manipulation of the BLA-PrL pathway did not affect PrL activity patterns during S- and SN-trials (Chi-squared test). All data are represented as mean ± SEM. For ANOVAs, * indicates statistical significance for *post hoc* Bonferroni comparisons, and $^{\#}$ indicates statistical significance for the main effect. ** = $p < 0.01$, $^{\#\#} = p < 0.01$. *ns* = non-significant ($p > 0.05$). See **Supplemental Table 3** for details on the statistical analyses.

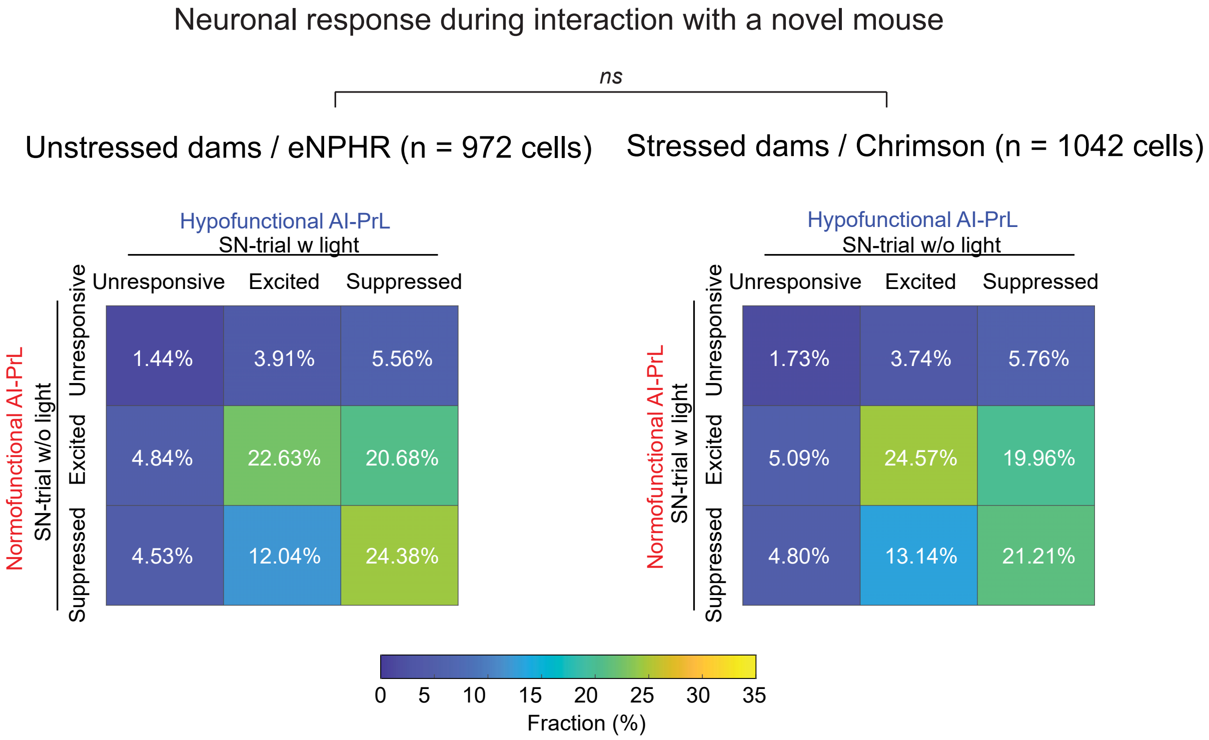
Neuronal response during interaction with a novel mouse
ns
Unstressed dams / eNPHR (n = 972 cells)
Stressed dams / Chrimson (n = 1042 cells)
Hypofunctional AI-PrL
SN-trial w light
Unresponsive
Excited
Suppressed
Normofunctional AI-PrL
SN-trial w/o light
1.44%
3.91%
5.56%
4.84%
22.63%
20.68%
4.53%
12.04%
24.38%
Hypofunctional AI-PrL
SN-trial w/o light
Normofunctional AI-PrL
SN-trial w light
1.73%
3.74%
5.76%
5.09%
24.57%
19.96%
4.80%
13.14%
21.21%
0 5 10 15 20 25 30 35
Fraction (%)

Supplementary Fig. 7. Optogenetic activation of the AI-PrL pathway in stressed dams and optogenetic inhibition of AI-PrL pathway in unstressed dams showed opposite effects on patterns of PrL activity changes between SN-trials with and without light stimulation. There were no significant differences between patterns of PrL activity changes in stressed dams from SN-trials with optogenetic activation to SN-trials without optogenetic activation, and those in unstressed dams from SN-trials without optogenetic inhibition to SN-trials with optogenetic inhibition (Chi-squared test). *ns* = non-significant ($p > 0.05$). See **Supplemental Table 3** for details on the statistical analyses.

Supplementary Fig. 8

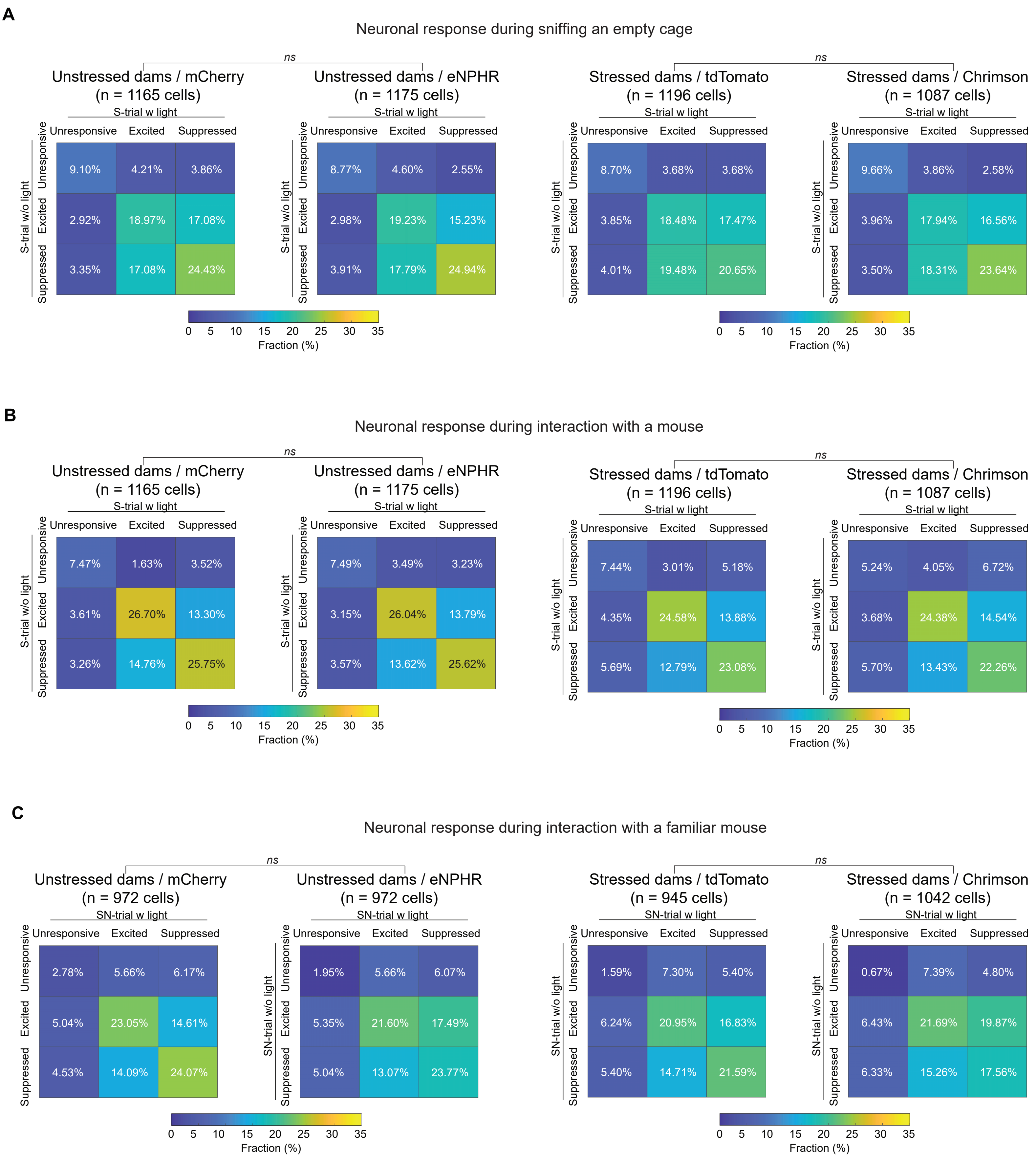

Supplementary Fig. 8. AI-PrL pathway did not affect patterns of PrL activity changes during S-trials and interaction with a familiar mouse in SN-trials. (**A** and **B**) Optogenetic manipulation of the AI-PrL pathway did not affect patterns of PrL activity changes from S-trials without light stimulation to S-trials with light stimulation (Chi-squared test). (**C**) Optogenetic manipulation of the AI-PrL pathway did not affect patterns of PrL activity changes during interaction with a familiar mouse from SN-trials without light stimulation to SN-trials with light stimulation (Chi-squared test). *ns* = non-significant ($p > 0.05$). See **Supplemental Table 3** for details on the statistical analyses.

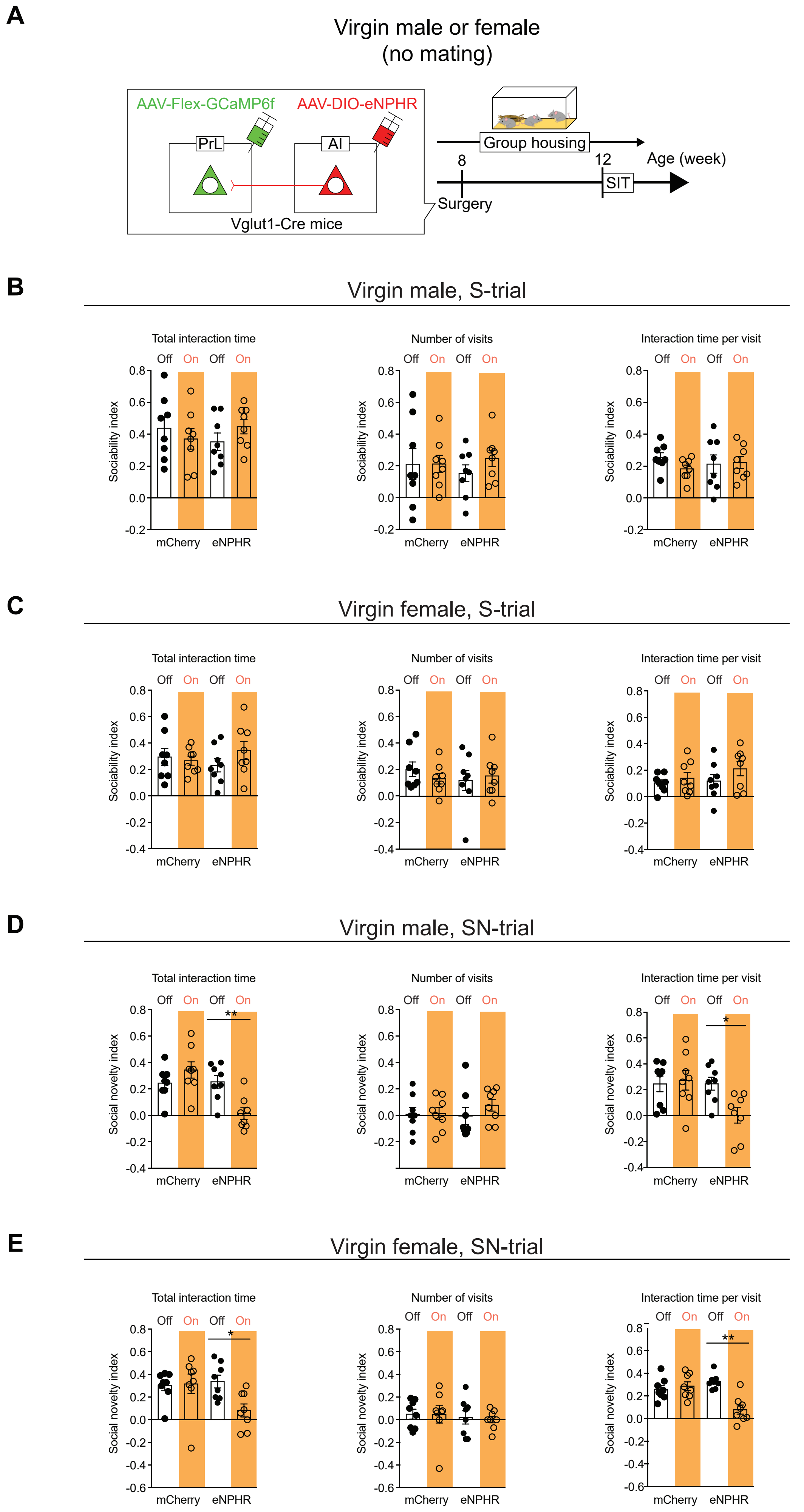
A
Virgin male or female
(no mating)
AAV-Flex-GCaMP6f
AAV-DIO-eNPHR
PrL
AI
Vglut1-Cre mice
Group housing
8
12
Age (week)
SIT
Surgery
B
Virgin male, S-trial
C
Virgin female, S-trial
D
Virgin male, SN-trial
E
Virgin female, SN-trial
Total interaction time
Number of visits
Interaction time per visit
Off
On
Sociability index
Social novelty index
mCherry
eNPHR
*
**

Supplementary Fig. 9. Hypofunction of the AI-PrL pathway in virgin mice led to behavioral changes in social novelty recognition, but not sociability. (**A**) Experimental timeline of SIT in virgin male and female mice. (**B** and **C**) Optogenetic inhibition of the AI-PrL pathway in virgin male and female mice did not affect sociability (two-way mixed ANOVA, Related samples sign test). N = 8 mice. (**D** and **E**) Optogenetic inhibition of the AI-PrL pathway in virgin male and female mice decreased total interaction time and interaction time per visit with novel mouse, but not the number of visits (two-way mixed ANOVA, Wilcoxon signed-rank test). N = 8 mice. All data are represented as mean ± SEM. For ANOVAs, * indicates statistical significance for *post hoc* Bonferroni comparisons. * = $p < 0.05$, ** = $p < 0.01$. See **Supplemental Table 3** for details on the statistical analyses.

Supplementary Fig. 10

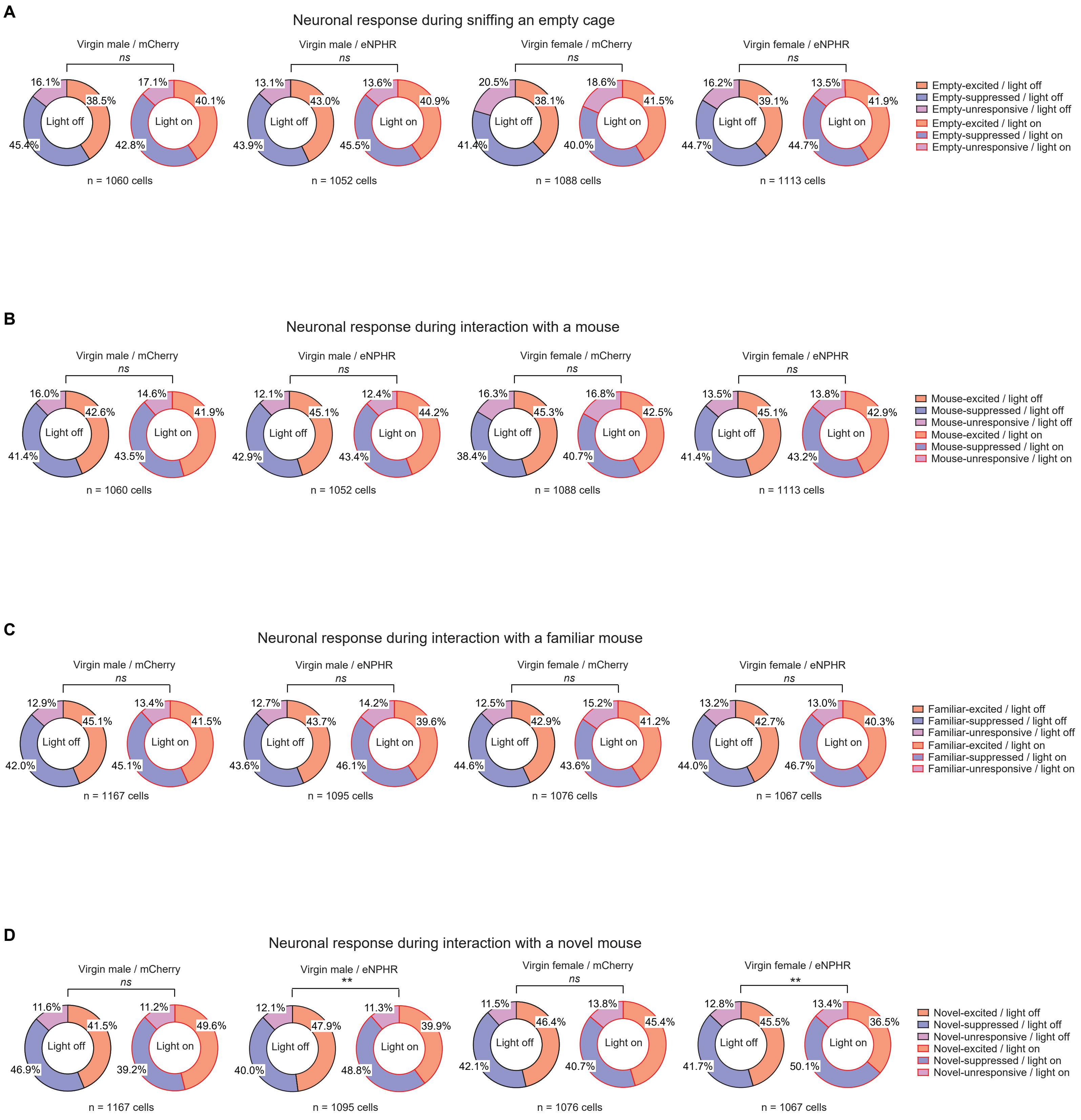

Supplementary Fig. 10. Effects of optogenetic inhibition of the AI-PrL pathway on PrL activity in virgin mice were similar to those in unstressed dams. (**A** and **B**) Optogenetic inhibition of the AI-PrL pathway in virgin mice did not affect PrL activity patterns during S-trials (Chi-squared test). (**C**) Optogenetic inhibition of the AI-PrL in virgin mice did not affect PrL activity patterns during interaction with a familiar mouse (Chi-squared test). (**D**) Optogenetic inhibition of the AI-PrL pathway in virgin mice resulted in similar patterns of PrL activity during interaction with a novel mouse to those observed in unstressed dams (Chi-squared test). *ns* = non-significant ($p > 0.05$). ** = $p < 0.01$. See **Supplemental Table 3** for details on the statistical analyses.

A

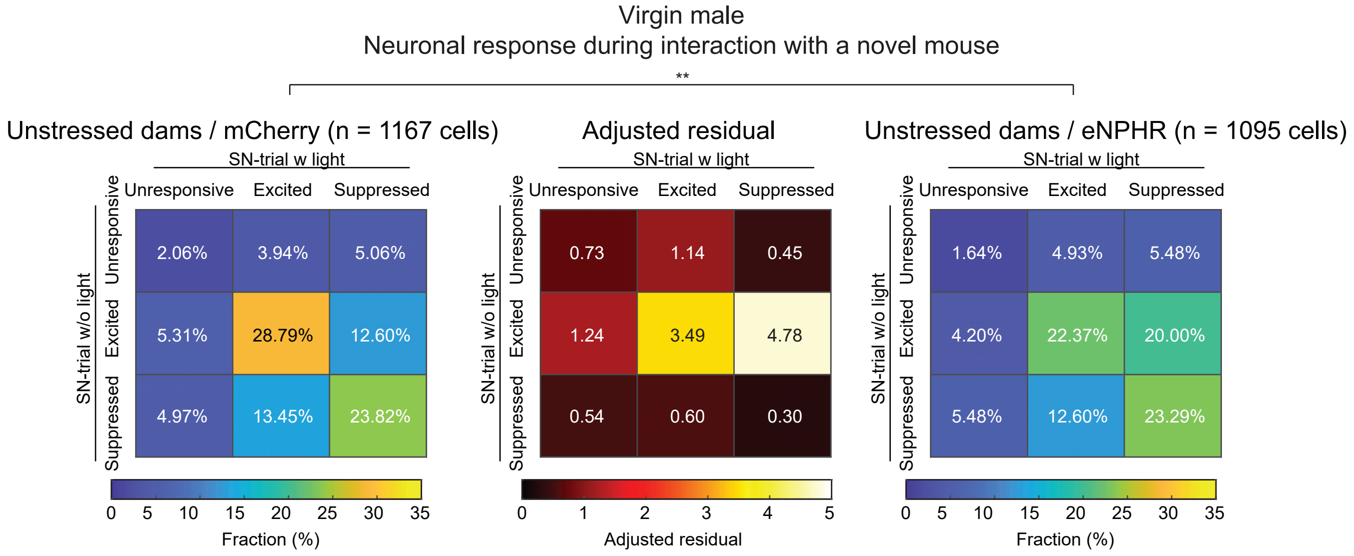
Virgin male
Neuronal response during interaction with a novel mouse
**
Unstressed dams / mCherry (n = 1167 cells)
Adjusted residual
Unstressed dams / eNPHR (n = 1095 cells)
SN-trial w light
SN-trial w/o light
Unresponsive
Excited
Suppressed
2.06%
3.94%
5.06%
5.31%
28.79%
12.60%
4.97%
13.45%
23.82%
0.73
1.14
0.45
1.24
3.49
4.78
0.54
0.60
0.30
1.64%
4.93%
5.48%
4.20%
22.37%
20.00%
5.48%
12.60%
23.29%
0 5 10 15 20 25 30 35
Fraction (%)
0 1 2 3 4 5
Adjusted residual

B

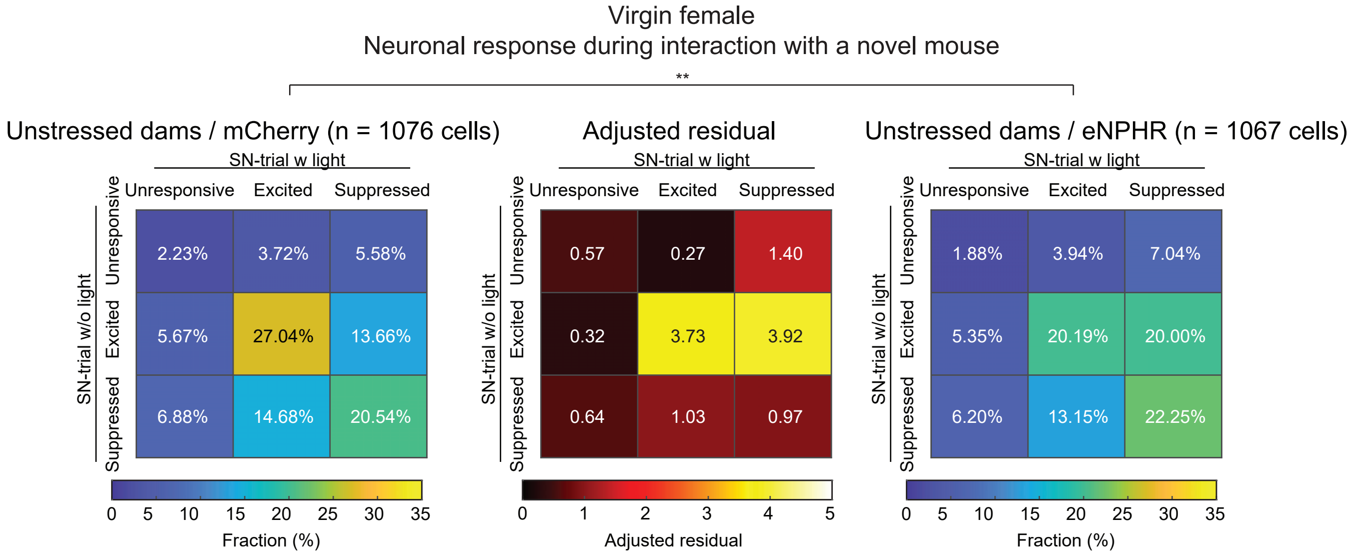
Virgin female
Neuronal response during interaction with a novel mouse
**
Unstressed dams / mCherry (n = 1076 cells)
Adjusted residual
Unstressed dams / eNPHR (n = 1067 cells)
SN-trial w light
SN-trial w/o light
Unresponsive
Excited
Suppressed
2.23%
3.72%
5.58%
5.67%
27.04%
13.66%
6.88%
14.68%
20.54%
0.57
0.27
1.40
0.32
3.73
3.92
0.64
1.03
0.97
1.88%
3.94%
7.04%
5.35%
20.19%
20.00%
6.20%
13.15%
22.25%
0 5 10 15 20 25 30 35
Fraction (%)
0 1 2 3 4 5
Adjusted residual

Supplementary Fig. 11. Optogenetic inhibition of the AI-PrL pathway in virgin mice resulted in similar changes in PrL activity patterns to those observed in unstressed dams. (**A** and **B**) Significant differences in the patterns of PrL activity changes from SN-trials without light stimulation to SN-trials with light stimulation were observed between virgin mice expressing control viruses and eNPHR (Chi-squared test). ** = $p < 0.01$. See **Supplemental Table 3** for details on the statistical analyses.

Supplementary Fig. 12

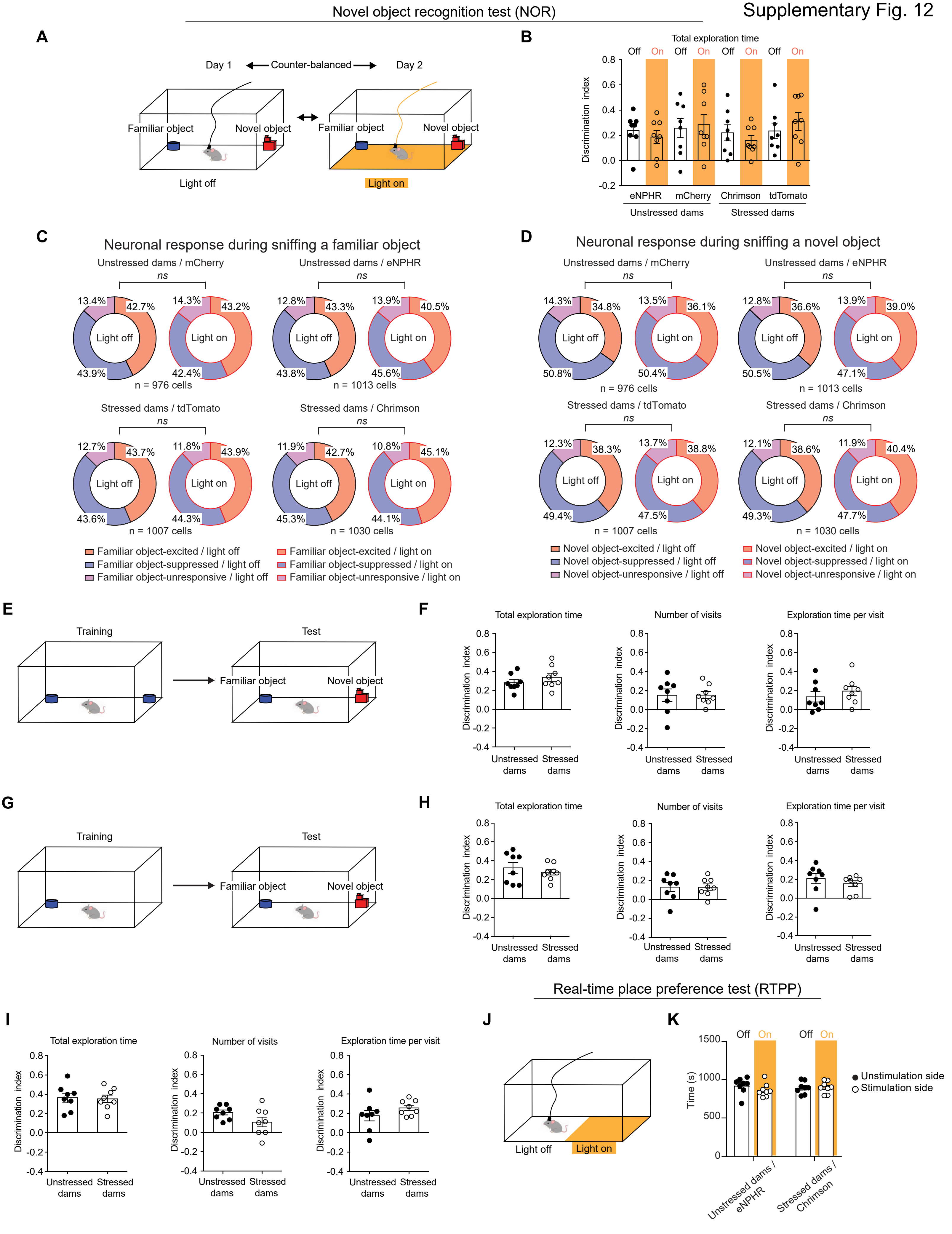

Supplementary Fig. 12. AI-PrL pathway did not affect non-social object recognition and valence processing. (**A**) Experimental timeline of NOR without and with optogenetic manipulation. (**B**) Optogenetic manipulation of the AI-PrL pathway did not affect novel object preference in both stressed and unstressed dams (three-way mixed ANOVA). N = 8 mice. (**C** and **D**) Optogenetic manipulation of the AI-PrL pathway did not affect PrL activity patterns during interaction with familiar or novel objects (Chi-squared test). **(E)** Scheme for NOR test. **(F)** NOR on postpartum day seven did not show significant differences between unstressed and stressed dams (Student's *t* test). N = 8 mice. **(G)** Scheme for modified NOR, analogous to SIT. **(H)** Modified NOR on postpartum day seven did not show significant differences between unstressed and stressed dams (Student's *t* test, Welch *t* test, Mann Whitney U test). N = 8 mice. **(I)** Modified NOR on postpartum day 13 did not show significant differences between unstressed and stressed dams (Student's *t* test). N = 8 mice. (**J**) Scheme for RTPP test. (**K**) Optogenetic manipulation of the AI-PrL pathway did not induce either reward or aversion for the side of the chamber (two-way mixed ANOVA). N = 8 mice. All data are represented as mean ± SEM. *ns* = non-significant ($p > 0.05$). See **Supplemental Table 3** for details on the statistical analyses.

Supplementary Fig. 13

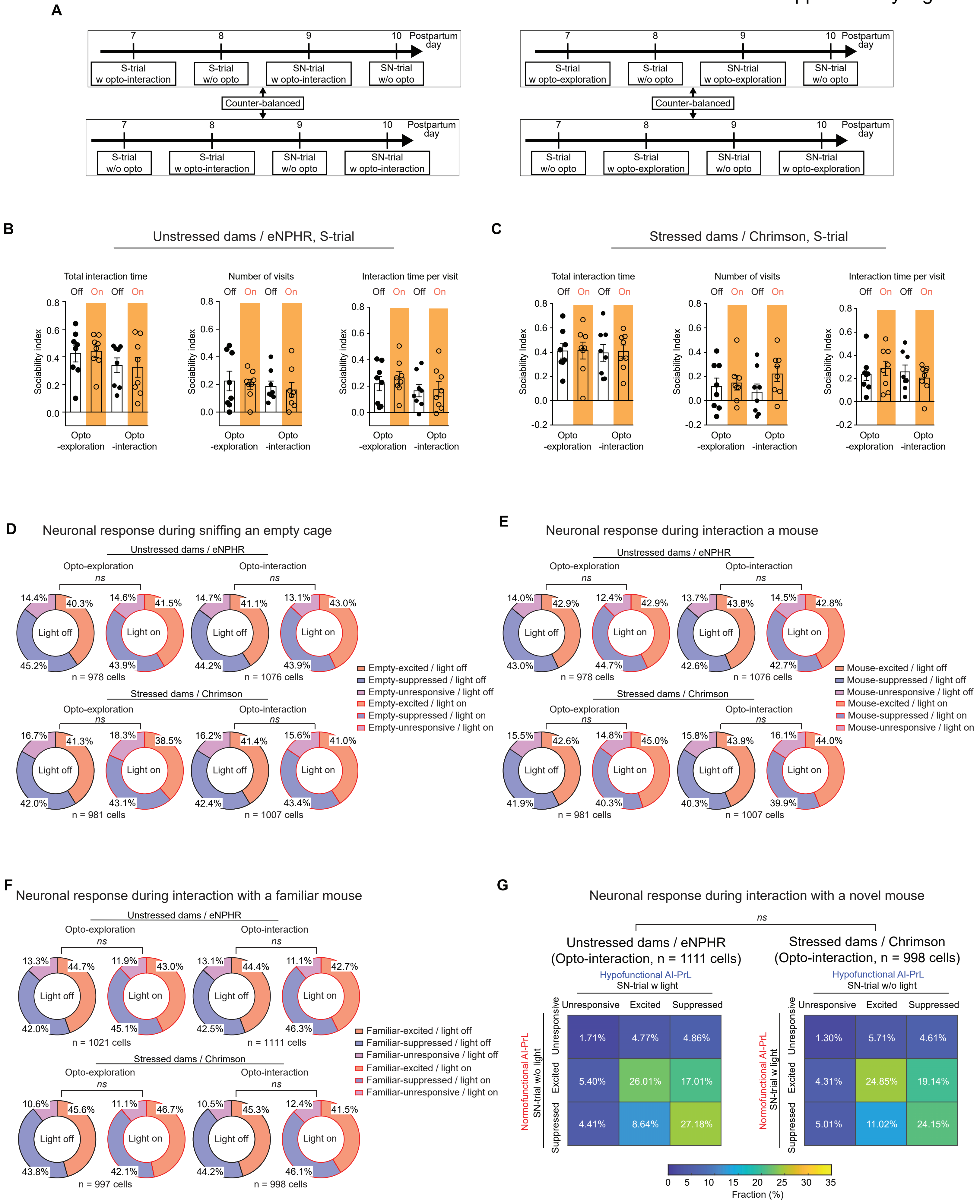

Supplementary Fig. 13. Behavioral closed-loop optogenetic manipulation of the AI-PrL pathway did not affect sociability and PrL activity during S-trials. (**A**) Experimental timeline of SIT with and without behavioral closed-loop optogenetic manipulation. (**B** and **C**) Behavioral closed-loop optogenetic manipulation did not affect sociability in both unstressed and stressed dams (two-way mixed ANOVA, Wilcoxon signed-rank test). N = 8 mice. (**D** and **E**) Behavioral closed-loop optogenetic manipulation did not affect PrL activity patterns during S-trials (Chi-squared test). (**F**) Behavioral closed-loop optogenetic manipulation did not affect PrL activity patterns during interaction with a familiar mouse in SN-trials (Chi-squared test). (**G**) There were no significant differences between patterns of PrL activity changes in stressed dams from SN-trials with optogenetic activation during interaction to SN-trials without optogenetic activation, and those in unstressed dams from SN-trials without optogenetic inhibition to SN-trials with optogenetic inhibition during interaction (Chi-squared test). All data are represented as mean ± SEM. *ns* = non-significant ($p > 0.05$). See **Supplemental Table 3** for details on the statistical analyses.

A

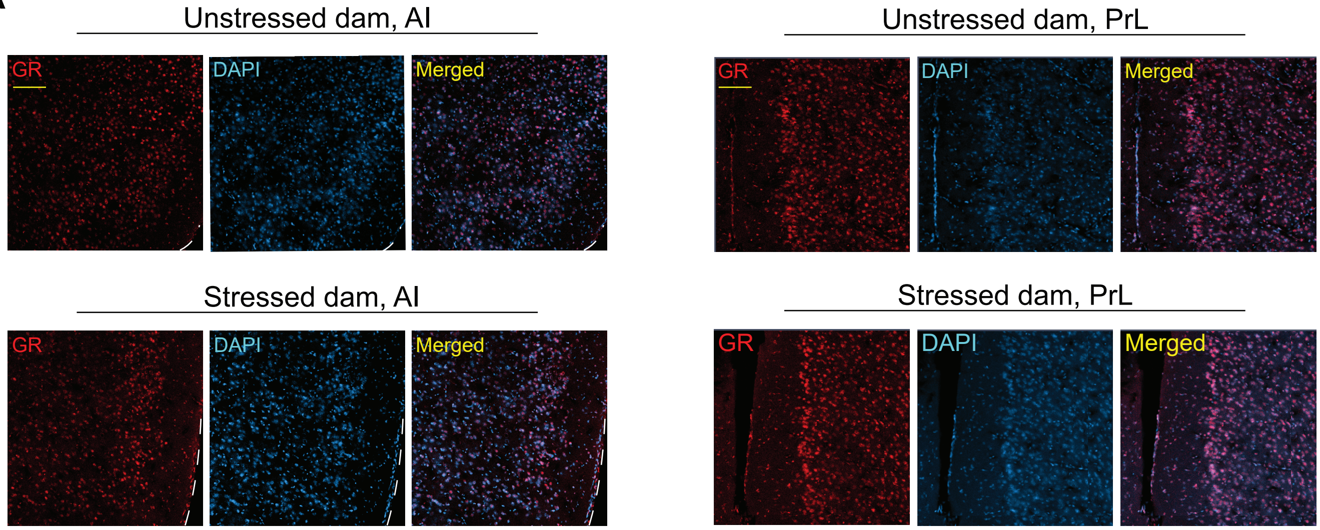
Unstressed dam, AI
GR
DAPI
Merged
Unstressed dam, PrL
GR
DAPI
Merged
Stressed dam, AI
GR
DAPI
Merged
Stressed dam, PrL
GR
DAPI
Merged

B

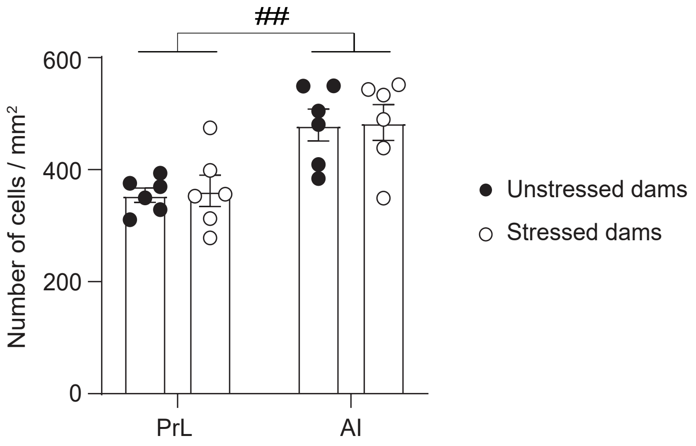
##
600
400
200
0
Number of cells / mm²
PrL
AI
Unstressed dams
Stressed dams

Supplementary Fig. 14. Expression level of the glucocorticoid receptor (GR) was not affected by SILA. (A) Representative images of GR^+ (red) cells in PrL and AI of unstressed and stressed dams. Scale bar, 100 µm. **(B)** There were no significant differences in the expression level of GR both in PrL and AI between unstressed and stressed dams (two-way mixed ANOVA). N = 6 mice. All data are represented as mean ± SEM. $^{\#\#}$ = ANOVA main effect for brain region, $p < 0.01$. See **Supplemental Table 3** for details on the statistical analyses.

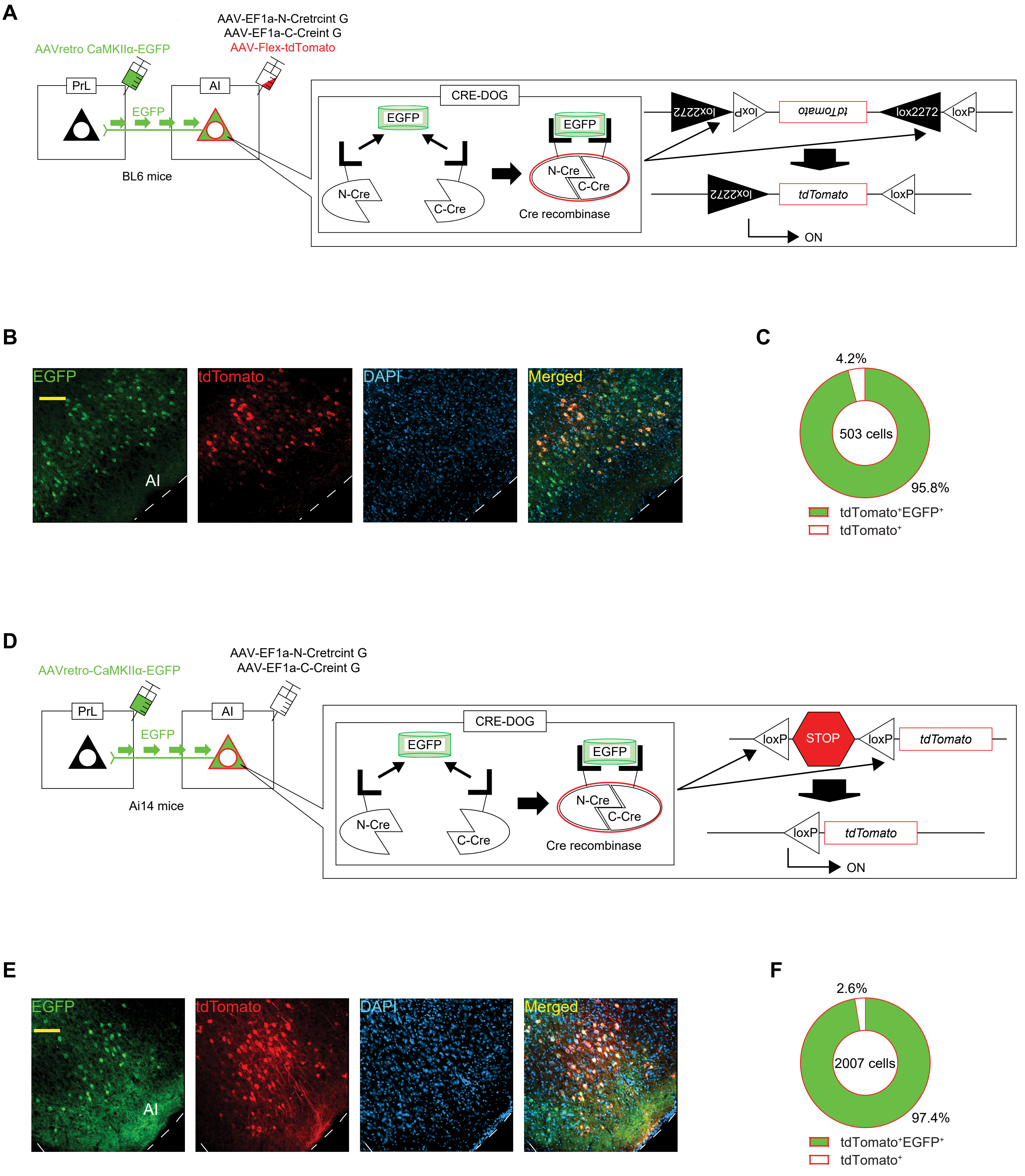
A
AAVretro CaMKIIα-EGFP
AAV-EF1a-N-Cretrcint G
AAV-EF1a-C-Creint G
AAV-Flex-tdTomato
PrL
AI
EGFP
BL6 mice
CRE-DOG
EGFP
N-Cre
C-Cre
Cre recombinase
lox2272
loxP
tdTomato
ON
B
EGFP
tdTomato
DAPI
Merged
AI
C
4.2%
503 cells
95.8%
tdTomato⁺EGFP⁺
tdTomato⁺
D
AAVretro-CaMKIIα-EGFP
AAV-EF1a-N-Cretrcint G
AAV-EF1a-C-Creint G
Ai14 mice
STOP
E
F
2.6%
2007 cells
97.4%

Supplementary Fig. 15. Cre recombinase was successfully expressed in the AI-PrL pathway by the CRE-DOG method. (**A**) Scheme of the CRE-DOG validation study with B6J mice injected with AAV-Flex-tdTomato. (**B**) Representative images of $EGFP^+$ (green), $tdTomato^+$ (red), DAPI (blue), and colocalized cells in AI of B6J mice. Scale bar, 100 μm. (**C**) CRE-DOG method successfully expressed tdTomato in cells expressing EGFP in B6J mice. (**D**) Scheme of the CRE-DOG validation study with Ai14 mice. (**E**) Representative images of $EGFP^+$ (green), $tdTomato^+$ (red), DAPI (blue), and colocalized cells in AI of Ai14 mice. (**F**) CRE-DOG method successfully expressed tdTomato in cells expressing EGFP in Ai14 mice. Scale bar, 100 μm.

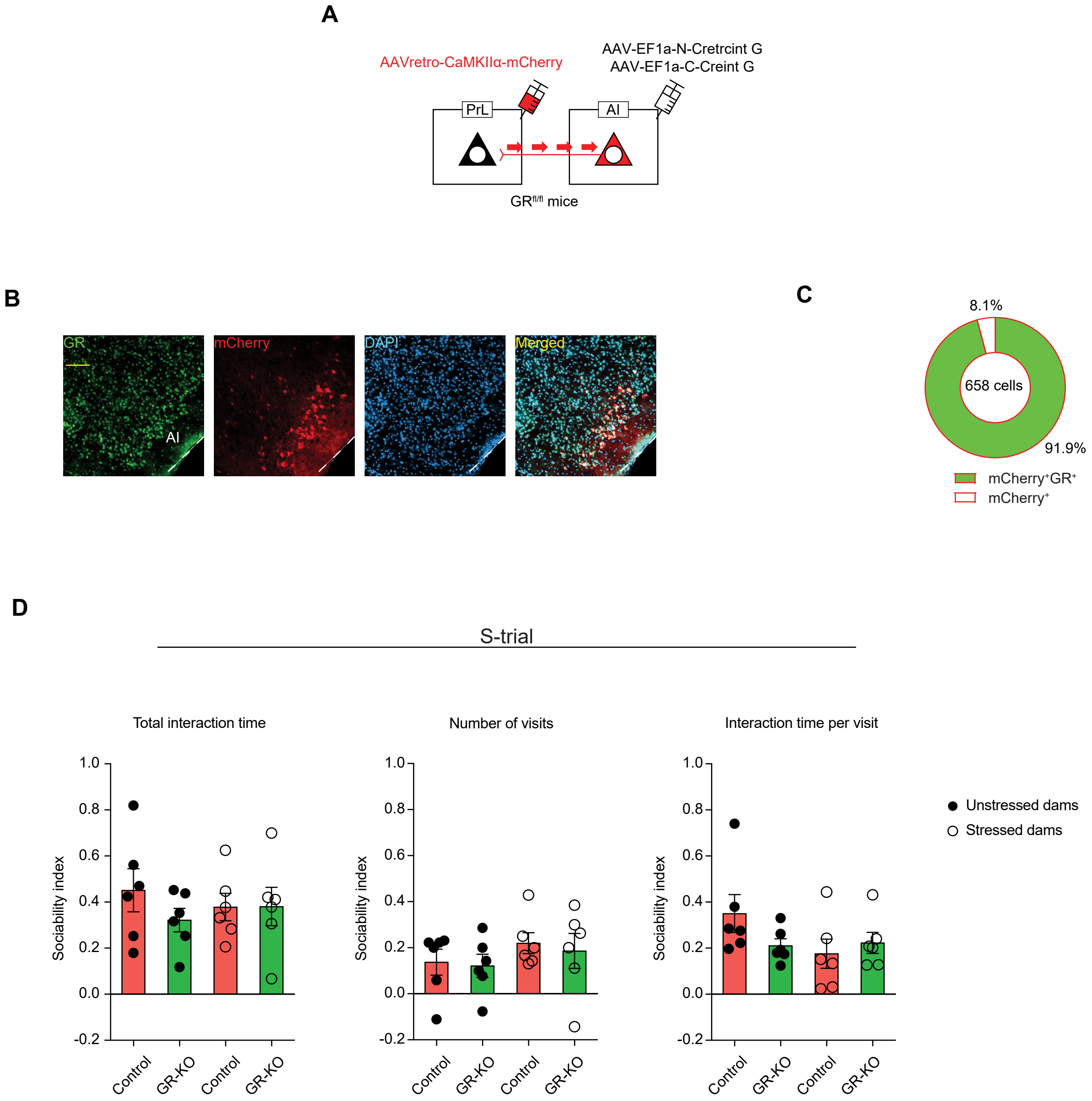
A
AAVretro-CaMKIIα-mCherry
AAV-EF1a-N-Cretrcint G
AAV-EF1a-C-Creint G
PrL
AI
GRfl/fl mice
B
GR
mCherry
DAPI
Merged
AI
C
8.1%
658 cells
91.9%
mCherry+GR+
mCherry+
D
S-trial
Total interaction time
Number of visits
Interaction time per visit
Sociability index
Control
GR-KO
Unstressed dams
Stressed dams

Supplementary Fig. 16. Pathway-specific GR-KO did not affect the results of S-trials. (**A**) Scheme of the virus strategy in $GR^{fl/fl}$ mice injected with AAV-retro-CaMKIIα-mCherry instead of AAV2-retro CaMKIIα-EGFP. (**B**) Representative images of GR^+, $mCherry^+$, DAPI, and colocalized cells in AI of $GR^{fl/fl}$ mice without CRE-DOG (no Cre expression in the AI-PrL pathway). Scale bar, 100 μm. (**C**) GR expression was not deleted in $GR^{fl/fl}$ mice without CRE-DOG. (**D**) No significant change was identified in sociability in AI-PrL pathway-specific GR-KO mice (two-way ANOVA). N = 6 mice. All data are represented as mean ± SEM. See **Supplemental Table 3** for details on the statistical analyses.

Supplementary Table 1. Abbreviations of brain areas.

Abbreviation	Brain area
MO	Medial orbital cortex
VO	Ventral orbital cortex
LO	Lateral orbital cortex
M1	Primary motor cortex
M2	Secondary motor cortex
Cg	Cingulate cortex
IL	Infralimbic cortex
AM	Anteromedial nucleus of thalamus
VM	Ventromedial nucleus of thalamus
RSA	Retrosplenial agran cortex
V2	Secondary visual cortex
V1	Primary visual cortex
S2	Secondary somatosensory cortex
MD	Mediodorsal nucleus of thalamus
RE	Nucleus reuniens of thalamus
DEN	Dorsal endopiriform nucleus
BMA	Basomedial nucleus of amygdala
BLA	Basolateral nucleus of amygdala
ECT	Ectorhinal cortex
PRh	Perirhinal cortex
LEnt	Lateral entorhinal cortex
Pir	Piriform cortex
S	Subiculum
CA1	CA1 region of hippocampus
DR	Dorsal raphe nucleus
VTA	Ventral tegmental area

Supplementary Table 2. Definitions of subsets of PrL neurons.

Behavioral test	Event used for ROC analysis	Name	Definition
S-trial of SIT	Sniffing an empty cage	Empty-excited / light off	Neurons which that are excited during sniffing an empty cage in S-trials without light stimulation
		Empty-suppressed / light off	Neurons that are suppressed during sniffing an empty cage in S-trials without light stimulation
		Empty-unresponsive / light off	Neurons that are not excited or suppressed during sniffing an empty cage in S-trials without light stimulation
	Interaction with a mouse	Mouse-excited / light off	Neurons that are excited during interaction with a mouse in S-trials without light stimulation
		Mouse-suppressed / light off	Neurons that are suppressed during interaction with a mouse in S-trials without light stimulation
		Mouse-unresponsive / light off	Neurons that are not excited or suppressed during interaction with a mouse in S-trials without light stimulation
	Sniffing an empty cage	Empty-excited / light on	Neurons that are excited during sniffing an empty cage in S-trials with light stimulation
		Empty-suppressed / light on	Neurons that are suppressed during sniffing an empty cage in S-trials with light stimulation
		Empty-unresponsive / light on	Neurons that are not excited or suppressed during sniffing an empty cage in S-trials with light stimulation
	Interaction with a mouse	Mouse-excited / light on	Neurons that are excited during interaction with a mouse in S-trials with light stimulation
		Mouse-suppressed / light on	Neurons that are suppressed during interaction with a mouse in S-trials with light stimulation
		Mouse-unresponsive / light on	Neurons that are not excited or suppressed during interaction with a mouse in S-trials with light stimulation
SN-trial of SIT	Interaction with a familiar mouse	Familiar-excited / light off	Neurons that are excited during interaction with a familiar mouse in SN-trials without light stimulation
		Familiar-suppressed / light off	Neurons that are suppressed during interaction with a familiar mouse in SN-trials without light stimulation
		Familiar-unresponsive / light off	Neurons that are not excited or suppressed during interaction with a familiar mouse in SN-trials without light stimulation

	Interaction with a novel mouse	Novel-excited / light off	Neurons that are excited during interaction with a novel mouse in SN-trials without light stimulation
		Novel-suppressed / light off	Neurons that are suppressed during interaction with a novel mouse in SN-trials without light stimulation
		Novel-unresponsive / light off	Neurons that are not excited or suppressed during interaction with a novel mouse in SN-trials without light stimulation
	Interaction with a familiar mouse	Familiar-excited / light on	Neurons that are excited during interaction with a familiar mouse in SN-trials with light stimulation
		Familiar-suppressed / light on	Neurons that are suppressed during interaction with a familiar mouse in SN-trials with light stimulation
		Familiar-unresponsive / light on	Neurons that are not excited or suppressed during interaction with a familiar mouse in SN-trials with light stimulation
	Interaction with a novel mouse	Novel-excited / light on	Neurons that are excited during interaction with a novel mouse in SN-trials with light stimulation
		Novel-suppressed / light on	Neurons that are suppressed during interaction with a novel mouse in SN-trials with light stimulation
		Novel-unresponsive / light on	Neurons that are not excited or suppressed during interaction with a novel mouse in SN-trials with light stimulation
NOR	Sniffing a familiar object	Familiar object-excited / light off	Neurons that are excited during sniffing a familiar object in NOR without light stimulation
		Familiar object-suppressed / light off	Neurons that are suppressed during sniffing a familiar object in NOR without light stimulation
		Familiar object-unresponsive / light off	Neurons that are not excited or suppressed during sniffing a familiar object in NOR without light stimulation
	Sniffing a novel object	Novel object-excited / light off	Neurons that are excited during sniffing a novel object in NOR without light stimulation
		Novel object-suppressed / light off	Neurons that are suppressed during sniffing a novel object in NOR without light stimulation
		Novel object-unresponsive / light off	Neurons that are not excited or suppressed during sniffing a novel object in NOR without light stimulation

	Sniffing a familiar object	Familiar object-excited / light on	Neurons that are excited during sniffing a familiar object in NOR with light stimulation
		Familiar object-suppressed / light on	Neurons that are suppressed during sniffing a familiar object in NOR with light stimulation
		Familiar object-unresponsive / light on	Neurons that are not excited or suppressed during sniffing a familiar object in NOR with light stimulation
	Sniffing a novel object	Novel object-excited / light on	Neurons that are excited during sniffing a novel object in NOR with light stimulation
		Novel object-suppressed / light on	Neurons that are suppressed during sniffing a novel object in NOR with light stimulation
		Novel object-unresponsive / light on	Neurons that are not excited or suppressed during sniffing a novel object in NOR with light stimulation
Note that neurons with a suppressed response as defined with this method do not necessarily exhibit an immediate decrease of activity during the corresponding behavior, but rather display an overall negative correlation with the corresponding behavior.			

Supplementary Table 3. Detailed information on statistical analyses and sample sizes.

Statistical tests performed for each data set are shown.

In the ANOVA designs, (W) indicates a within-subjects factor, and (B) indicates a between-subjects factor.

"Opsin" is used as a factor in several designs, meaning that groups have been treated (+) with and (-) without opsin expression.

In the optogenetics experiments, "OFF" and "ON" are used to indicate the light conditions.

Figure	Shapiro-Wilk test	Levene's test	Two-Way mixed ANOVA
1C	Total interaction time		
	Unstressed Empty *p* = 0.595	Empty *p* = 0.330	Interaction F(1,14) = 0.214 , *p* = 0.651 , partial η^2 = 0.015
	Stressed Empty *p* = 0.178	Mouse *p* = 0.171	Sociability (W) F(1,14) = 64.333 , *p* = <0.001 , partial η^2 = 0.821
	Unstressed Mouse *p* = 0.104		Stress (B) F(1,14) = 0.014 , *p* = 0.907 , partial η^2 = 0.001
	Stressed Mouse *p* = 0.329		
		Box's M test	post hoc (Bonferroni)
		p = 0.335	Unstressed Empty x Mouse *p* = <0.001
			Stressed Empty x Mouse *p* = <0.001
	Number of visits		**Two-Way mixed ANOVA**
	Unstressed Empty *p* = 0.085	Empty *p* = 0.082	Interaction F(1,14) = 0.009 , *p* = 0.924 , partial η^2 = <0.001
	Stressed Empty *p* = 0.424	Mouse *p* = 0.230	Sociability (W) F(1,14) = 15.668 , *p* = 0.001 , partial η^2 = 0.528
	Unstressed Mouse *p* = 0.067		Stress (B) F(1,14) = 0.002 , *p* = 0.966 , partial η^2 = <0.001
	Stressed Mouse *p* = 0.535		
		Box's M test	post hoc (Bonferroni)
		p = 0.632	Unstressed Empty x Mouse *p* = 0.016
			Stressed Empty x Mouse *p* = 0.012
	Interaction time per visit		**Two-Way mixed ANOVA**
	Unstressed Empty *p* = 0.386	Empty *p* = 0.580	Interaction F(1,14) = 0.081 , *p* = 0.780 , partial η^2 = 0.006
	Stressed Empty *p* = 0.111	Mouse *p* = 0.265	Sociability (W) F(1,14) = 12.206 , *p* = 0.004 , partial η^2 = 0.466
	Unstressed Mouse *p* = 0.111		Stress (B) F(1,14) = 0.478 , *p* = 0.501 , partial η^2 = 0.033
	Stressed Mouse *p* = 0.610		
	**Sample Size**	Box's M test	post hoc (Bonferroni)
	Female ♀ Unstressed *n* = 8	*p* = 0.038	Unstressed Empty x Mouse *p* = 0.018
	Female ♀ Stressed *n* = 8		Stressed Empty x Mouse *p* = 0.040
**Figure**	**Shapiro-Wilk test**	**Levene's test**	**Student t-Test Independent samples, 2-tailed**
**1C (Socialbility index)**	Total interaction time		
	Unstressed *p* = 0.991	*p* = 0.302	*t* (14) = -0.331 , *p* = 0.746
	Stressed *p* = 0.600		Cohen's d = -0.165
	Number of visits		**Student t-Test Independent samples, 2-tailed**
	Unstressed *p* = 0.293	*p* = 0.493	*t* (14) = -0.127 , *p* = 0.900
	Stressed *p* = 0.356		Cohen's d = -0.064
	Interaction time per visit		**Student t-Test Independent samples, 2-tailed**
	Unstressed *p* = 0.092	*p* = 0.537	*t* (14) = 0.215 , *p* = 0.833
	Stressed *p* = 0.268		Cohen's d = 0.108
	**Sample Size**		
	Female ♀ Unstressed *n* = 8		
	Female ♀ Stressed *n* = 8		
**Figure**	**Shapiro-Wilk test**	**Levene's test**	**Two-Way mixed ANOVA**
**1E**	Total interaction time		
	Unstressed Familiar *p* = 0.245	Familiar *p* = 0.316	Interaction F(1,14) = 90.513 , *p* = <0.001 , partial η^2 = 0.866
	Stressed Familiar *p* = 0.058	Novel *p* = 0.934	Social novelty (W) F(1,14) = 71.536 , *p* = <0.001 , partial η^2 = 0.836
	Unstressed Novel *p* = 0.517		Stress (B) F(1,14) = 0.567 , *p* = 0.464 , partial η^2 = 0.039
	Stressed Novel *p* = 0.901		
		Box's M test	post hoc (Bonferroni)
		p = 0.048	Unstressed Familiar x Novel *p* = <0.001
			Stressed Familiar x Novel *p* = 0.468
	Number of visits		**Two-Way mixed ANOVA**
	Unstressed Familiar *p* = 0.117	Familiar *p* = 0.405	Interaction F(1,14) = 0.003 , *p* = 0.960 , partial η^2 = <0.001
	Stressed Familiar *p* = 0.861	Novel *p* = 0.211	Social novelty (W) F(1,14) = 0.064 , *p* = 0.803 , partial η^2 = 0.005
	Unstressed Novel *p* = 0.164		Stress (B) F(1,14) = 0.344 , *p* = 0.567 , partial η^2 = 0.024
	Stressed Novel *p* = 0.551		
		Box's M test	post hoc (Bonferroni)
		p = 0.496	Unstressed Familiar x Novel *p* = 0.833
			Stressed Familiar x Novel *p* = 0.888
	Interaction time per visit		**Two-Way mixed ANOVA**
	Unstressed Familiar *p* = 0.098	Familiar *p* = 0.141	Interaction F(1,14) = 6.425 , *p* = 0.024 , partial η^2 = 0.315
	Stressed Familiar *p* = 0.387	Novel *p* = 0.616	Social novelty (W) F(1,14) = 7.481 , *p* = 0.016 , partial η^2 = 0.348

	Unstressed Novel $p =$ 0.050		Stress (B) $F(1,14) =$ 0.001 , $p =$ 0.979 , partial $\eta^2 =$ <0.001
	Stressed Novel $p =$ 0.062		
	**Sample Size**	Box's M test	post hoc (Bonferroni)
	Female ♀ Unstressed $n =$ 8	$p =$ 0.249	Unstressed Familiar x Novel $p =$ 0.002
	Female ♀ Stressed $n =$ 8		Stressed Familiar x Novel $p =$ 0.889
**Figure 1E (social novelty index)**	**Shapiro-Wilk test**	**Levene's test**	**Student t-Test Independent samples, 2-tailed**
	Total interaction time		
	Unstressed $p =$ 0.771	$p =$ 0.094	t (14) = 9.531 , $p =$ <0.001
	Stressed $p =$ 0.292		Cohen's d = 4.766
	Number of visits		**Student t-Test Independent samples, 2-tailed**
	Unstressed $p =$ 0.813	$p =$ 0.797	t (14) = 0.1373 , $p =$ 0.893
	Stressed $p =$ 0.745		Cohen's d = 0.0687
	Interaction time per visit		**Student t-Test Independent samples, 2-tailed**
	Unstressed $p =$ 0.767	$p =$ 0.248	t (14) = 2.9561 , $p =$ 0.010
	Stressed $p =$ 0.659		Cohen's d = 1.478
	**Sample Size**		
	Female ♀ Unstressed $n =$ 8		
	Female ♀ Stressed $n =$ 8		
**Figure 1G**	**Shapiro-Wilk test**	**Levene's test**	**Two-Way mixed ANOVA**
	Unstressed Vglut $p =$ 0.501	Vglut $p =$ 0.970	Interaction F(1,14) = 36.148 , $p =$ <0.001 , partial $\eta^2 =$ 0.721
	Stressed Vglut $p =$ 0.343	Vgat $p =$ 0.823	Marker (W) F(1,14) = 150.36 , $p =$ <0.001 , partial $\eta^2 =$ 0.915
	Unstressed Vgat $p =$ 0.192		Stress (B) F(1,14) = 34.523 , $p =$ <0.001 , partial $\eta^2 =$ 0.711
	Stressed Vgat $p =$ 0.145		
		Box's M test	post hoc (Bonferroni)
		$p =$ 0.618	Vglut Unstressed x Stressed $p =$ <0.001
			Vgat Unstressed x Stressed $p =$ 0.699
	**Sample Size**		
	Female ♀ Unstressed $n =$ 8		
	Female ♀ Stressed $n =$ 8		
**Figure 1I**	**Shapiro-Wilk test**	**Levene's test**	**Two-Way mixed ANOVA**
	Unstressed Vglut $p =$ 0.699	Vglut $p =$ 0.823	Interaction F(1,14) = 55.763 , $p =$ <0.001 , partial $\eta^2 =$ 0.799
	Stressed Vglut $p =$ 0.127	Vgat $p =$ 0.266	Marker (W) F(1,14) = 112.21 , $p =$ <0.001 , partial $\eta^2 =$ 0.889
	Unstressed Vgat $p =$ 0.607		Stress (B) F(1,14) = 67.6 , $p =$ <0.001 , partial $\eta^2 =$ 0.828
	Stressed Vgat $p =$ 0.222		
		Box's M test	post hoc (Bonferroni)
		$p =$ 0.112	Vglut Unstressed x Stressed $p =$ <0.001
			Vgat Unstressed x Stressed $p =$ 0.233
	**Sample Size**		
	Female ♀ Unstressed $n =$ 8		
	Female ♀ Stressed $n =$ 8		
**Figure 1K**	**Shapiro-Wilk test**	**Levene's test**	**Two-Way mixed ANOVA**
	Unstressed Vglut $p =$ 0.139	Vglut $p =$ 0.198	Interaction F(1,14) = 7.8963 , $p =$ 0.013 , partial $\eta^2 =$ 0.345
	Stressed Vglut $p =$ 0.350	Vgat $p =$ 0.608	Marker (W) F(1,14) = 7.8963 , $p =$ 0.013 , partial $\eta^2 =$ 0.345
	Unstressed Vgat $p =$ 0.851		Stress (B) F(1,14) = 12.165 , $p =$ 0.003 , partial $\eta^2 =$ 0.448
	Stressed Vgat $p =$ 0.318		
		Box's M test	post hoc (Bonferroni)
		$p =$ 0.657	Vglut Unstressed x Stressed $p =$ <0.001
			Vgat Unstressed x Stressed $p =$ 0.864
	**Sample Size**		
	Female ♀ Unstressed $n =$ 8		
	Female ♀ Stressed $n =$ 8		
**Figure 2E**	**Shapiro-Wilk test**	**Levene's test**	**Two-Way mixed ANOVA**
	Total interaction time		
	mCherry OFF $p =$ 0.061	OFF $p =$ 0.358	Interaction F(1,14) = 4.528 , $p =$ 0.052 , partial $\eta^2 =$ 0.244
	eNPHR OFF $p =$ 0.511	ON $p =$ 0.441	Light (W) F(1,14) = 14.698 , $p =$ 0.002 , partial $\eta^2 =$ 0.512
	mCherry ON $p =$ 0.623		Opsin (B) F(1,14) = 18.049 , $p =$ <0.001 , partial $\eta^2 =$ 0.563
	eNPHR ON $p =$ 0.716		
		Box's M test	post hoc (Bonferroni)
		$p =$ 0.365	mCherry ON x OFF $p =$ 0.248
			eNPHR ON x OFF $p =$ <0.001
	Number of visits		**Wilcoxon signed-rank test, two-sided**
	mCherry OFF $p =$ 0.795	OFF $p =$ 0.658	mCherry ON x OFF , $p =$ 0.735 , $z =$ -0.338
	eNPHR OFF $p =$ 0.269	ON $p =$ 0.450	$r =$ -0.085
	mCherry ON $p =$ 0.040		eNPHR ON x OFF , $p =$ 0.575 , $z =$ -0.560
	eNPHR ON $p =$ 0.358		$r =$ -0.140
	Interaction time per visit		**Two-Way mixed ANOVA**

	mCherry OFF $p =$ 0.214<br>eNPHR OFF $p =$ 0.744<br>mCherry ON $p =$ 0.891<br>eNPHR ON $p =$ 0.706<br><br>**Sample Size**<br>Female ♀ mCherry $n =$ 8<br>Female ♀ eNPHR $n =$ 8	OFF $p =$ 0.656<br>ON $p =$ 0.393<br><br>Box's M test<br>$p =$ 0.531	Interaction F(1,14) = 2.8073 , $p =$ 0.116 , partial $\eta^2 =$ 0.167<br>Light (W) F(1,14) = 8.7977 , $p =$ 0.010 , partial $\eta^2 =$ 0.386<br>Opsin (B) F(1,14) = 6.8209 , $p =$ 0.021 , partial $\eta^2 =$ 0.328<br><br>post hoc (Bonferroni)<br>mCherry ON x OFF $p =$ 0.377<br>eNPHR ON x OFF $p =$ 0.005
**Figure 2G**	**Shapiro-Wilk test**<br>Total interaction time<br>tdTomato OFF p = 0.860<br>Chrimson OFF p = 0.643<br>tdTomato ON p = 0.907<br>Chrimson ON p = 0.525<br><br>Number of visits<br>tdTomato OFF $p =$ 0.138<br>Chrimson OFF $p =$ 0.744<br>tdTomato ON $p =$ 0.102<br>Chrimson ON $p =$ 0.435<br><br>Interaction time per visit<br>tdTomato OFF $p =$ 0.234<br>Chrimson OFF $p =$ 0.071<br>tdTomato ON $p =$ 0.650<br>Chrimson ON $p =$ 0.383<br>**Sample Size**<br>Female ♀ tdTomato $n =$ 8<br>Female ♀ Chrimson $n =$ 8	**Levene's test**<br><br>OFF p = 0.032<br>ON p = 0.527<br><br><br><br>OFF $p =$ 0.107<br>ON $p =$ 0.627<br><br>Box's M test<br>$p =$ 0.012<br><br>OFF $p =$ 0.323<br>ON $p =$ 0.034	**Wilcoxon signed-rank test, two-sided**<br>tdTomato ON x OFF $p =$ 0.674 $z =$ 0.420<br>$r =$ 0.105<br>Chrimson ON x OFF $p =$ 0.012 $z =$ 2.521<br>$r =$ 0.630<br><br>**Two-Way mixed ANOVA**<br>Interaction F(1,14) = 0.1500 , $p =$ 0.704 , partial $\eta^2 =$ 0.011<br>Light (W) F(1,14) = 1.8462 , $p =$ 0.196 , partial $\eta^2 =$ 0.117<br>Opsin (B) F(1,14) = 0.0022 , $p =$ 0.963 , partial $\eta^2 =$ <0.001<br><br>post hoc (Bonferroni)<br>tdTomato ON x OFF $p =$ 0.237<br>Chrimson ON x OFF $p =$ 0.503<br><br>**Wilcoxon signed-rank test, two-sided**<br>tdTomato ON x OFF $p =$ 0.123 $z =$ 1.542<br>$r =$ 0.386<br>Chrimson ON x OFF $p =$ 0.025 $z =$ 2.240<br>$r =$ 0.56
**Figure 2M**	**Sample Size (# cells)**<br>Unstressed mCherry = 972<br>Stressed tdTomato = 945<br>**Sample Size (#animals)**<br>Female ♀ tdTomato $n =$ 8<br>Female ♀ mCherry $n =$ 8	**Expected Count <5**<br>0%	**Chi-square test of homogeneity**<br>tdtomato x mCherry $p =$ 0.842 $\chi^2(2) =$ 0.343<br>Phi = 0.013
**Figure 2N**	**Sample Size (# cells)**<br>Unstressed mCherry = 972<br>Stressed tdTomato = 945<br>**Sample Size (#animals)**<br>Female ♀ tdTomato $n =$ 8<br>Female ♀ mCherry $n =$ 8	**Expected Count <5**<br>0%	**Chi-square test of homogeneity**<br>tdtomato x mCherry $p =$ <0.001 $\chi^2(2) =$ 24.7<br><br>Phi = 0.114
**Figure 2O**	**Sample Size (# cells)**<br>Unstressed mCherry = 972<br>Unstressed eNPHR = 972<br>Stressed tdTomato = 945<br>Stressed Chrimson = 1042<br>**Sample Size (#animals)**<br>Female ♀ tdTomato $n =$ 8<br>Female ♀ Chrimson $n =$ 8	**Expected Count <5**<br>0%	**Chi-square test of homogeneity**<br>Unstressed mCherry ON x OFF $p =$ 0.673<br>Unstressed eNPHR ON x OFF $p =$ <0.001<br>Stressed tdTomato ON x OFF $p =$ 0.320<br>Stressed Chrimson ON x OFF $p =$ <0.001
**Figure 3B**	**Sample Size (# cells)**<br>Unstressed mCherry = 972<br>Stressed tdTomato = 945<br>**Sample Size (#animals)**<br>Female ♀ tdTomato $n =$ 8<br>Female ♀ mCherry $n =$ 8	**Expected Count <5**<br>0%	**Chi-square test of homogeneity**<br>tdtomato x mCherry $p =$ <0.001 $\chi^2(8) =$ 34.67<br>Phi = 0.134
**Figure 3D**	**Sample Size (# cells)**<br>Unstressed mCherry = 972<br>Unstressed eNPHR = 972<br>**Sample Size (#animals)**<br>Female ♀ mCherry $n =$ 8<br>Female ♀ eNPHR $n =$ 8	**Expected Count <5**<br>0%	**Chi-square test of homogeneity**<br>eNPHR x mCherry $p =$ <0.001 $\chi^2(8) =$ 35.49<br>Phi = 0.135
**Figure 3E**	**Sample Size (# cells)**<br>Stressed tdTomato = 972<br>Stressed Chrimson = 1042<br>**Sample Size (#animals)**<br>Female ♀ tdTomato $n =$ 8<br>Female ♀ Chrimson $n =$ 8	**Expected Count <5**<br>0%	**Chi-square test of homogeneity**<br>tdTomato x mCherry $p =$ <0.001 $\chi^2(8) =$ 34.29<br>Phi = 0.131

**Figure**	**Shapiro-Wilk test**	**Levene's test**	**Wilcoxon signed-rank test, two-sided**
**4C**	Total interaction time		
	Exploration OFF *p* = 0.827	OFF *p* = 0.322	Exploration ON x OFF *p* = 0.401 z = 0.84
	Interaction OFF *p* = 0.290	ON *p* = 0.179	*r* = 0.210
	Exploration ON *p* = 0.046		Interaction ON x OFF *p* = 0.036 z = 2.10
	Interaction ON *p* = 0.590		*r* = 0.525
	Number of visits		**Two-Way mixed ANOVA**
	Exploration OFF *p* = 0.530	OFF *p* = 0.817	Interaction $F(1,14)$ = 0.3836 , *p* = 0.546 , partial η^2 = 0.027
	Interaction OFF *p* = 0.375	ON *p* = 0.260	Light (W) $F(1,14)$ = 1.1049 , *p* = 0.311 , partial η^2 = 0.073
	Exploration ON *p* = 0.655		Condition (B) $F(1,14)$ = 0.0843 , *p* = 0.776 , partial η^2 = 0.006
	Interaction ON *p* = 0.708		
		Box's M test	post hoc (Bonferroni)
		p = 0.749	Exploration ON x OFF *p* = 0.257
			Interaction ON x OFF *p* = 0.765
	Interaction time per visit		**Two-Way mixed ANOVA**
	Exploration OFF *p* = 0.300	OFF *p* = 0.514	Interaction $F(1,14)$ = 9.772 , *p* = 0.007 , partial η^2 = 0.411
	Interaction OFF *p* = 0.165	ON *p* = 0.460	Light (W) $F(1,14)$ = 4.8626 , *p* = 0.045 , partial η^2 = 0.258
	Exploration ON *p* = 0.463		Condition (B) $F(1,14)$ = 1.6049 , *p* = 0.226 , partial η^2 = 0.103
	Interaction ON *p* = 0.388		
	**Sample Size**	Box's M test	post hoc (Bonferroni)
	Female ♀ Exploration *n* = 8	*p* = 0.402	Exploration ON x OFF *p* = 0.525
	Female ♀ Interaction *n* = 8		Interaction ON x OFF *p* = 0.002
**Figure**	**Shapiro-Wilk test**	**Levene's test**	**Two-Way mixed ANOVA**
**4E**	Total interaction time		
	Exploration OFF *p* = 0.445	OFF *p* = 0.948	Interaction $F(1,14)$ = 10.735 , *p* = 0.006 , partial η^2 = 0.434
	Interaction OFF *p* = 0.598	ON *p* = 0.641	Light (W) $F(1,14)$ = 13.664 , *p* = 0.002 , partial η^2 = 0.494
	Exploration ON *p* = 0.795		Condition (B) $F(1,14)$ = 13.766 , *p* = 0.002 , partial η^2 = 0.496
	Interaction ON *p* = 0.631		
		Box's M test	post hoc (Bonferroni)
		p = 0.983	Exploration ON x OFF *p* = 0.771
			Interaction ON x OFF *p* = <0.001
	Number of visits		**Two-Way mixed ANOVA**
	Exploration OFF *p* = 0.275	OFF *p* = 0.498	Interaction $F(1,14)$ = 2.236 , *p* = 0.157 , partial η^2 = 0.138
	Interaction OFF *p* = 0.886	ON *p* = 0.219	Light (W) $F(1,14)$ = 0.1076 , *p* = 0.748 , partial η^2 = 0.008
	Exploration ON *p* = 0.407		Condition (B) $F(1,14)$ = 0.9752 , *p* = 0.340 , partial η^2 = 0.065
	Interaction ON *p* = 0.837		
		Box's M test	post hoc (Bonferroni)
		p = 0.626	Exploration ON x OFF *p* = 0.423
			Interaction ON x OFF *p* = 0.218
	Interaction time per visit		**Two-Way mixed ANOVA**
	Exploration OFF *p* = 0.191	OFF *p* = 0.389	Interaction $F(1,14)$ = 1.2811 , *p* = 0.277 , partial η^2 = 0.084
	Interaction OFF *p* = 0.097	ON *p* = 0.490	Light (W) $F(1,14)$ = 6.1802 , *p* = 0.026 , partial η^2 = 0.306
	Exploration ON *p* = 0.560		Condition (B) $F(1,14)$ = 2.8065 , *p* = 0.116 , partial η^2 = 0.167
	Interaction ON *p* = 0.331		
	**Sample Size**	Box's M test	post hoc (Bonferroni)
	Female ♀ Exploration *n* = 8	*p* = 0.720	Exploration ON x OFF *p* = 0.355
	Female ♀ Interaction *n* = 8		Interaction ON x OFF *p* = 0.023
**Figure**	**Sample Size (# cells)**	**Expected Count <5**	**Chi-square test of homogeneity**
**4F**	eNPHR/Exploration = 1021	0%	Exploration eNPHR ON x OFF *p* = 0.551
	eNPHR/Interaction = 1111		Interaction eNPHR ON x OFF *p* = 0.001
	Chrimson/Exploration = 997		Exploration Chrimson ON x OFF *p* = 0.327
	Chrimson/Interaction = 998		Interaction Chrimson ON x OFF *p* = 0.002
	**Sample Size (#animals)**		
	Female ♀ eNPHR Exploration *n* = 8		
	Female ♀ eNPHR Interaction *n* = 8		
	Female ♀ Chrimson Exploration *n* = 8		
	Female ♀ Chrimson Interaction *n* = 8		
**Figure**	**Shapiro-Wilk test**	**Levene's test**	**One-way ANOVA**
**5C**	EGFP expression level		
	Week 1 *p* = 0.179	*p* = 0.587	Week (B) $F(2,11)$ = 96.155 , *p* = <0.001 , partial η^2 = 0.955
	Week 2 *p* = 0.319		
	Week 3 *p* = 0.460		post hoc (Bonferroni)
			Week 1 x Week 2 *p* = 0.039
			Week 1 x Week 3 *p* = <0.001
			Week 2 x Week 3 *p* = <0.001
	GR expression level		**Welch's ANOVA**
	Week 1 *p* = 0.703	*p* = 0.001	

	Week 2 *p* = 0.554		Week (B) F(2,5) = 86.756 , *p* = <0.001 , partial η^2 = 0.966
	Week 3 *p* = 0.292		
			post hoc (Games-Howell)
			Week 1 x Week 2 *p* = 0.012
			Week 1 x Week 3 *p* = 0.001
			Week 2 x Week 3 *p* = 0.001
	**Sample Size**		
	Female ♀ Week 1 *n* = 4		
	Female ♀ Week 2 *n* = 4		
	Female ♀ Week 3 *n* = 4		
**Figure 5D**	**Shapiro-Wilk test**	**Levene's test**	**Two-Way ANOVA**
	Total interaction time		
	Unstressed Control *p* = 0.787	*p* = 0.302	Interaction F(3,20) = 3.418 , *p* = 0.079 , partial η^2 = 0.146
	Unstressed GR-KO *p* = 0.698		Stress (B) F(3,20) = 1.531 , *p* = 0.230 , partial η^2 = 0.071
	Stressed Control *p* = 0.082		KO (B) F(3,20) = 7.821 , *p* = 0.011 , partial η^2 = 0.281
	Stressed GR-KO *p* = 0.354		
			post hoc (Bonferroni)
			Unstressed Control x GR-KO *p* = 0.510
			Stressed Control x GR-KO *p* = 0.004
	Number of visits		**Mann Whitney U test, two-sided**
	Unstressed Control *p* = 0.135	*p* = 0.044	Unstressed Control x GR-KO *p* = 0.699 *U* = 15.00
	Unstressed GR-KO *p* = 0.056		*r* = -0.12 *z* = -0.48
	Stressed Control *p* = 0.261		Stressed Control x GR-KO *p* = 0.589 *U* = 14.00
	Stressed GR-KO *p* = 0.727		*r* = -0.16 *z* = -0.64
	Interaction time per visit		**Two-Way ANOVA**
	Unstressed Control *p* = 0.996	*p* = 0.475	Interaction F(3,20) = 4.2815 , *p* = 0.052 , partial η^2 = 0.176
	Unstressed GR-KO *p* = 0.600		Stress (B) F(3,20) = 3.1581 , *p* = 0.091 , partial η^2 = 0.136
	Stressed Control *p* = 0.260		KO (B) F(3,20) = 9.2584 , *p* = 0.006 , partial η^2 = 0.316
	Stressed GR-KO *p* = 0.395		
			post hoc (Bonferroni)
			Unstressed Control x GR-KO *p* = 0.499
			Stressed Control x GR-KO *p* = 0.002
	**Sample Size**		
	Female ♀ Unstressed Control *n* = 6		
	Female ♀ Unstressed GR-KO *n* = 6		
	Female ♀ Stressed Control *n* = 6		
	Female ♀ Stressed GR-KO *n* = 6		
**Figure 5F**	**Shapiro-Wilk test**	**Levene's test**	**Two-Way ANOVA**
	AI		
	Unstressed Control p = 0.669	*p* = 0.309	Interaction F(3,20) = 10.027 , *p* = 0.005 , partial η^2 = 0.334
	Unstressed GR-KO p = 0.520		Stress (B) F(3,20) = 17.218 , *p* = <0.001 , partial η^2 = 0.463
	Stressed Control p = 0.454		KO (B) F(3,20) = 7.720 , *p* = 0.012 , partial η^2 = 0.279
	Stressed GR-KO p = 0.315		
			post hoc (Bonferroni)
			Unstressed Control x GR-KO *p* = 0.787
			Stressed Control x GR-KO *p* = <0.001
			Control Unstressed x Stressed *p* = <0.001
	PrL		**Two-Way ANOVA**
	Unstressed Control p = 0.570	*p* = 0.231	Interaction F(3,20) = 8.251 , *p* = 0.009 , partial η^2 = 0.292
	Unstressed GR-KO p = 0.767		Stress (B) F(3,20) = 26.178 , *p* = <0.001 , partial η^2 = 0.567
	Stressed Control p = 0.461		KO (B) F(3,20) = 9.619 , *p* = 0.006 , partial η^2 = 0.325
	Stressed GR-KO p = 0.320		
	**Sample Size**		post hoc (Bonferroni)
	Female ♀ Unstressed Control *n* = 6		Unstressed Control x GR-KO *p* = 0.873
	Female ♀ Unstressed GR-KO *n* = 6		Stressed Control x GR-KO *p* = <0.001
	Female ♀ Stressed Control *n* = 6		Control Unstressed x Stressed *p* = <0.001
	Female ♀ Stressed GR-KO *n* = 6		
**Figure S1B**	**Shapiro-Wilk test**	**Levene's test**	**Student t-Test Independent samples, 2-tailed**
	Total interaction time		
	Unstressed *p* = 0.571	*p* = 0.326	*t* (14) = 0.918 , *p* = 0.374
	Stressed *p* = 0.526		Cohen's d = 0.459
	Number of visits		**Student t-Test Independent samples, 2-tailed**
	Unstressed *p* = 0.076	*p* = 0.840	*t* (14) = 0.0215 , *p* = 0.983
	Stressed *p* = 0.176		Cohen's d = 0.0108
	Interaction time per visit		**Student t-Test Independent samples, 2-tailed**

		Unstressed *p* = 0.566	*p* = 0.466	*t* (14) = 0.9261 , *p* = 0.370
		Stressed *p* = 0.633		Cohen's d = 0.4631
		**Sample Size**		
		Female ♀ Unstressed *n* = 8		
		Female ♀ Stressed *n* = 8		
**Figure S1C**	**Shapiro-Wilk test**		**Levene's test**	**Student t-Test Independent samples, 2-tailed**
	Total interaction time			
		Unstressed *p* = 0.479	*p* = 0.572	*t* (14) = 0.3837 , *p* = 0.707
		Stressed *p* = 0.704		Cohen's d = 0.1919
	Number of visits			**Student t-Test Independent samples, 2-tailed**
		Unstressed *p* = 0.302	*p* = 0.548	*t* (14) = 0.1412 , *p* = 0.890
		Stressed *p* = 0.364		Cohen's d = 0.0706
	Interaction time per visit			**Student t-Test Independent samples, 2-tailed**
		Unstressed *p* = 0.813	*p* = 0.999	*t* (14) = 0.3646 , *p* = 0.721
		Stressed *p* = 0.824		Cohen's d = 0.1823
		**Sample Size**		
		Female ♀ Unstressed *n* = 8		
		Female ♀ Stressed *n* = 8		
**Figure S1D**	**Shapiro-Wilk test**		**Levene's test**	**Student t-Test Independent samples, 2-tailed**
	PrL			
		Unstressed *p* = 0.413	*p* = 0.062	*t* (14) = 3.345 , *p* = 0.005
		Stressed *p* = 0.820		Cohen's d = 1.672
		**Sample Size**		
		Female ♀ Unstressed *n* = 8		
		Female ♀ Stressed *n* = 8		
**Figure S1G**	**Shapiro-Wilk test**			**Paired Student t-Test, 2-tailed**
	Area			
	MO	Male ♂ *p* = 0.055		*t* (3) = 2.375 , *p* = 0.098 , Cohen's d = 1.188
	VO	Male ♂ *p* = 0.628		*t* (3) = 6.662 , *p* = 0.007 , Cohen's d = 3.331
	LO	Male ♂ *p* = 0.714		*t* (3) = 12.880 , *p* = 0.001 , Cohen's d = 6.440
	AI	Male ♂ *p* = 0.233		*t* (3) = 3.660 , *p* = 0.035 , Cohen's d = 1.830
	M1	Male ♂ *p* = 0.186		*t* (3) = 4.993 , *p* = 0.015 , Cohen's d = 2.496
	M2	Male ♂ *p* = 0.074		*t* (3) = 20.048 , *p* = <0.001 , Cohen's d = 10.024
	Cg	Male ♂ *p* = 0.597		*t* (3) = 14.311 , *p* = <0.001 , Cohen's d = 7.156
	PrL	Male ♂ *p* = 0.515		*t* (3) = -18.585 , *p* = <0.001 , Cohen's d = -9.292
	IL	Male ♂ *p* = 0.079		*t* (3) = 3.884 , *p* = 0.030 , Cohen's d = 1.942
	AM	Male ♂ *p* = 0.293		*t* (3) = 2.980 , *p* = 0.059 , Cohen's d = 1.490
	VM	Male ♂ *p* = 0.156		*t* (3) = 4.079 , *p* = 0.027 , Cohen's d = 2.040
	RSA	Male ♂ *p* = 0.115		*t* (3) = 3.155 , *p* = 0.051 , Cohen's d = 1.577
	V2	Male ♂ *p* = 0.130		*t* (3) = 13.462 , *p* = <0.001 , Cohen's d = 6.731
	V1	Male ♂ *p* = 0.262		*t* (3) = 7.577 , *p* = 0.005 , Cohen's d = 3.789
	MD	Male ♂ *p* = 0.572		*t* (3) = 37.044 , *p* = <0.001 , Cohen's d = 18.522
	RE	Male ♂ *p* = 0.144		*t* (3) = 2.307 , *p* = 0.104 , Cohen's d = 1.154
	DEN	Male ♂ *p* = 0.345		*t* (3) = 2.359 , *p* = 0.099 , Cohen's d = 1.180
	BMA	Male ♂ *p* = 0.567		*t* (3) = 7.920 , *p* = 0.004 , Cohen's d = 3.960
	BLA	Male ♂ *p* = 0.283		*t* (3) = 2.550 , *p* = 0.084 , Cohen's d = 1.275
	ECT	Male ♂ *p* = 0.301		*t* (3) = 3.146 , *p* = 0.051 , Cohen's d = 1.573
	PRh	Male ♂ *p* = 0.354		*t* (3) = 3.060 , *p* = 0.055 , Cohen's d = 1.530
	LEnt	Male ♂ *p* = 0.860		*t* (3) = 6.663 , *p* = 0.007 , Cohen's d = 3.332
	Pir	Male ♂ *p* = 0.245		*t* (3) = 4.522 , *p* = 0.020 , Cohen's d = 2.261
	S	Male ♂ *p* = 0.354		*t* (3) = 2.831 , *p* = 0.066 , Cohen's d = 1.416
	CA1	Male ♂ *p* = 0.065		*t* (3) = 2.602 , *p* = 0.080 , Cohen's d = 1.301
	VTA	Male ♂ *p* = 0.105		*t* (3) = 1.360 , *p* = 0.267 , Cohen's d = 0.680
	MO	Female ♀ *p* = 0.368		*t* (3) = 2.441 , *p* = 0.092 , Cohen's d = 1.220
	VO	Female ♀ *p* = 0.572		*t* (3) = 8.560 , *p* = 0.003 , Cohen's d = 4.280
	LO	Female ♀ *p* = 0.650		*t* (3) = 2.011 , *p* = 0.138 , Cohen's d = 1.005
	AI	Female ♀ *p* = 0.679		*t* (3) = 2.891 , *p* = 0.063 , Cohen's d = 1.445
	M1	Female ♀ *p* = 0.580		*t* (3) = 6.088 , *p* = 0.009 , Cohen's d = 3.044
	M2	Female ♀ *p* = 0.114		*t* (3) = 5.394 , *p* = 0.012 , Cohen's d = 2.697
	Cg	Female ♀ *p* = 0.558		*t* (3) = 6.209 , *p* = 0.008 , Cohen's d = 3.105
	PrL	Female ♀ *p* = 0.794		*t* (3) = -5.914 , *p* = 0.010 , Cohen's d = -2.957
	IL	Female ♀ *p* = 0.936		*t* (3) = 3.296 , *p* = 0.046 , Cohen's d = 1.648
	AM	Female ♀ *p* = 0.237		*t* (3) = 4.029 , *p* = 0.027 , Cohen's d = 2.015
	VM	Female ♀ *p* = 0.798		*t* (3) = 3.050 , *p* = 0.055 , Cohen's d = 1.525
	RSA	Female ♀ *p* = 0.192		*t* (3) = 3.530 , *p* = 0.039 , Cohen's d = 1.765
	V2	Female ♀ *p* = 0.513		*t* (3) = 10.226 , *p* = 0.002 , Cohen's d = 5.113
	V1	Female ♀ *p* = 0.279		*t* (3) = 2.901 , *p* = 0.062 , Cohen's d = 1.450
	S2	Female ♀ *p* = 0.644		*t* (3) = 7.165 , *p* = 0.006 , Cohen's d = 3.582
	MD	Female ♀ *p* = 0.458		*t* (3) = 10.561 , *p* = 0.002 , Cohen's d = 5.280
	RE	Female ♀ *p* = 0.104		*t* (3) = 1.311 , *p* = 0.281 , Cohen's d = 0.655
	DEN	Female ♀ *p* = 0.985		*t* (3) = 2.782 , *p* = 0.069 , Cohen's d = 1.391
	BMA	Female ♀ *p* = 0.561		*t* (3) = 5.063 , *p* = 0.015 , Cohen's d = 2.532
	BLA	Female ♀ *p* = 0.629		*t* (3) = 2.805 , *p* = 0.068 , Cohen's d = 1.403

Figure	Area	Shapiro-Wilk test		Levene's	Area	Test	Statistic		Effect size
	ECT	Female ♀ p =	0.304			t (3)	= 2.834 ,	p = 0.066 ,	Cohen's d = 1.417
	PRh	Female ♀ p =	0.056			t (3)	= 2.647 ,	p = 0.077 ,	Cohen's d = 1.323
	LEnt	Female ♀ p =	0.367			t (3)	= 5.561 ,	p = 0.011 ,	Cohen's d = 2.781
	Pir	Female ♀ p =	0.761			t (3)	= 2.857 ,	p = 0.065 ,	Cohen's d = 1.429
	S	Female ♀ p =	0.662			t (3)	= 3.855 ,	p = 0.031 ,	Cohen's d = 1.928
	CA1	Female ♀ p =	0.374			t (3)	= 3.312 ,	p = 0.045 ,	Cohen's d = 1.656
	DR	Female ♀ p =	0.062			t (3)	= 4.265 ,	p = 0.024 ,	Cohen's d = 2.132
	VTA	Female ♀ p =	0.676			t (3)	= 1.982 ,	p = 0.142 ,	Cohen's d = 0.991
						**Wilcoxon signed-rank test, two-sided**			
	S2	Male ♂ p =	0.017			Ipsi x Contra		p = 0.068	z = 1.826
									r = 0.646
	DR	Male ♂ p =	0.049			Ipsi x Contra		p = 0.068	z = 1.825
		**Sample Size**							r = 0.645
		Male ♂ n =	4						
		Female ♀ n =	4						

Figure	Area	Shapiro-Wilk test		Levene's	Area	Student t-Test Independent samples, 2-tailed			
**S1H**	Area				Area				
	MO	Unstressed p =	0.420	p = 0.054	MO	t (14)	= 0.4489 ,	p = 0.660 ,	Cohen's d = 0.224
	VO	Unstressed p =	0.951	p = 0.078	VO	t (14)	= -0.2627 ,	p = 0.797 ,	Cohen's d = -0.131
	LO	Unstressed p =	0.661	p = 0.799	LO	t (14)	= -0.1005 ,	p = 0.921 ,	Cohen's d = -0.050
	AI	Unstressed p =	0.896	p = 0.076	AI	t (14)	= 7.2071 ,	p = <0.001 ,	Cohen's d = 3.604
	M1	Unstressed p =	0.242	p = 0.815	M1	t (14)	= 0.7030 ,	p = 0.494 ,	Cohen's d = 0.352
	M2	Unstressed p =	0.846	p = 0.272	M2	t (14)	= 0.5075 ,	p = 0.620 ,	Cohen's d = 0.254
	Cg	Unstressed p =	0.679	p = 0.944	Cg	t (14)	= 1.1508 ,	p = 0.269 ,	Cohen's d = 0.575
	IL	Unstressed p =	0.431	p = 0.763	IL	t (14)	= 2.1112 ,	p = 0.053 ,	Cohen's d = 1.056
	AM	Unstressed p =	0.711	p = 0.692	AM	t (14)	= 1.0403 ,	p = 0.316 ,	Cohen's d = 0.520
	VM	Unstressed p =	0.358	p = 0.523	VM	t (14)	= -1.0253 ,	p = 0.323 ,	Cohen's d = -0.513
	RSA	Unstressed p =	0.849	p = 0.904	RSA	t (14)	= -0.6915 ,	p = 0.501 ,	Cohen's d = -0.346
	V2	Unstressed p =	0.061	p = 0.059	V2	t (14)	= 1.1576 ,	p = 0.266 ,	Cohen's d = 0.579
	V1	Unstressed p =	0.384	p = 0.682	V1	t (14)	= 0.3981 ,	p = 0.697 ,	Cohen's d = 0.199
	S2	Unstressed p =	0.409	p = 0.264	S2	t (14)	= 0.2366 ,	p = 0.816 ,	Cohen's d = 0.118
	MD	Unstressed p =	0.613	p = 0.239	MD	t (14)	= -1.8322 ,	p = 0.088 ,	Cohen's d = -0.916
	RE	Unstressed p =	0.294	p = 0.435	RE	t (14)	= -0.2929 ,	p = 0.774 ,	Cohen's d = -0.146
	DEN	Unstressed p =	0.562	p = 0.165	DEN	t (14)	= 0.4471 ,	p = 0.662 ,	Cohen's d = 0.224
	BMA	Unstressed p =	0.578	p = 0.822	BMA	t (14)	= -0.9130 ,	p = 0.377 ,	Cohen's d = -0.456
	BLA	Unstressed p =	0.596	p = 0.051	BLA	t (14)	= 2.8513 ,	p = 0.013 ,	Cohen's d = 1.426
	ECT	Unstressed p =	0.955	p = 0.299	ECT	t (14)	= 0.2354 ,	p = 0.817 ,	Cohen's d = 0.118
	PRh	Unstressed p =	0.085	p = 0.246	PRh	t (14)	= 0.0110 ,	p = 0.991 ,	Cohen's d = 0.006
	LEnt	Unstressed p =	0.523	p = 0.940	LEnt	t (14)	= -1.9096 ,	p = 0.077 ,	Cohen's d = -0.955
	Pir	Unstressed p =	0.237	p = 0.245	Pir	t (14)	= -0.4888 ,	p = 0.633 ,	Cohen's d = -0.244
	CA1	Unstressed p =	0.723	p = 0.536	CA1	t (14)	= 0.5443 ,	p = 0.595 ,	Cohen's d = 0.272
	DR	Unstressed p =	0.903	p = 0.114	DR	t (14)	= -0.1640 ,	p = 0.872 ,	Cohen's d = -0.082
	VTA	Unstressed p =	0.624	p = 0.517	VTA	t (14)	= -0.6980 ,	p = 0.496 ,	Cohen's d = -0.349
	MO	Stressed p =	0.880						
	VO	Stressed p =	0.782						
	LO	Stressed p =	0.130						
	AI	Stressed p =	0.561						
	M1	Stressed p =	0.886						
	M2	Stressed p =	0.411						
	Cg	Stressed p =	0.134						
	IL	Stressed p =	0.721						
	AM	Stressed p =	0.909						
	VM	Stressed p =	0.841						
	RSA	Stressed p =	0.463						
	V2	Stressed p =	0.879						
	V1	Stressed p =	0.476						
	S2	Stressed p =	0.964						
	MD	Stressed p =	0.308						
	RE	Stressed p =	0.109						
	DEN	Stressed p =	0.234						
	BMA	Stressed p =	0.152						
	BLA	Stressed p =	0.354						
	ECT	Stressed p =	0.719						
	PRh	Stressed p =	0.389						
	LEnt	Stressed p =	0.204						
	Pir	Stressed p =	0.636						
	CA1	Stressed p =	0.330						
	DR	Stressed p =	0.784						
	VTA	Stressed p =	0.337						
						**Mann Whitney U test**			
	S	Unstressed p =	0.026	p = 0.932		Unstressed x Stressed		p = 0.195	U = 45.00
	S	Stressed p =	0.888					r = 0.341	z = 1.37
		**Sample Size**							

	Female ♀ Unstressed n = 8<br>Female ♀ Stressed n = 8		
**Figure S1J**	**Shapiro-Wilk test**<br>Male ♂ Vglut p = 0.967<br>Male ♂ Vgat p = 0.796<br>Female ♀ Vglut p = 0.922<br>Female ♀ Vgat p = 0.153<br>**Sample Size**<br>Male ♂ Vglut n = 6<br>Female ♀ Vglut n = 6<br>Male ♂ Vgat n = 6<br>Female ♀ Vgat n = 6	**Levene's test**<br>Vglut p = 0.769<br>Vgat p = 0.126<br>Box's M test<br>p = 0.555	**Two-Way mixed ANOVA**<br>Interaction F(1,10) = 0.3396 , p = 0.573 , partial η^2 = 0.033<br>Marker (W) F(1,10) = 829.12 , p = <0.001 , partial η^2 = 0.988<br>Sex (B) F(1,10) = 0.0003 , p = 0.987 , partial η^2 = <0.001<br>post hoc (Bonferroni)<br>Male ♂ Vglut x Vgat p = <0.001<br>Female ♀ Vglut x Vgat p = <0.001
**Figure S2**	**NA**<br>**Sample Size**<br>Female ♀ B6J mice n = 4	**NA**	**NA**
**Figure S3A**	**Shapiro-Wilk test**<br>Modulation index<br>Unstressed mCherry p = <0.001<br>Unstressed eNPHR p = <0.001<br>Stressed tdTomato p = 0.001<br>Stressed Chrimson p = <0.001<br>**Sample Size (# animals)**<br>Female ♀ Unstressed mCherry n = 6<br>Female ♀ Unstressed eNPHR n = 6<br>Female ♀ Stressed tdTomato n = 6<br>Female ♀ Stressed Chrimson n = 6	**Sample Size (# cells)**<br>N= 512<br>N= 618<br>N= 708<br>N= 658	**Wilcoxon signed rank test (One Sample)**<br>Against 0.5<br>p = 0.692 , z = 0.396 r = 0.018<br>p = <0.001 , z = -8.283 r = -0.333<br>p = 0.714 , z = -0.367 r = -0.014<br>p = <0.001 , z = 6.784 r = 0.264
**Figure S3A (Modulation index)**	**Shapiro-Wilk test**<br>Modulation index<br>Unstressed mCherry p = <0.001<br>Unstressed eNPHR p = <0.001<br><br>Modulation index<br>Stressed tdTomato p = 0.001<br>Stressed Chrimson p = <0.001<br>**Sample Size (# animals)**<br>Female ♀ Unstressed mCherry n = 6<br>Female ♀ Unstressed eNPHR n = 6<br>Female ♀ Stressed tdTomato n = 6<br>Female ♀ Stressed Chrimson n = 6	**Levene's test**<br>p = 0.029<br><br>p = 0.175<br>**Sample Size (# cells)**<br>N= 512<br>N= 618<br>N= 708<br>N= 658	**Mann Whitney U test, two-sided**<br>mCherry x eNPHR p = <0.001 U = >1200<br>r = 0.163 z = 5.46<br><br>**Mann Whitney U test, two-sided**<br>tdTomato x Chrimson p = <0.001 U = >1200<br>r = 0.134 z = 4.97
**Figure S3B**	**Shapiro-Wilk test**<br>Relative Peak Amplitude<br>Unstressed mCherry p = <0.001<br>Unstressed eNPHR p = <0.001<br>Stressed tdTomato p = <0.001<br>Stressed Chrimson p = <0.001<br>**Sample Size (# animals)**<br>Female ♀ Unstressed mCherry n = 6<br>Female ♀ Unstressed eNPHR n = 6<br>Female ♀ Stressed tdTomato n = 6<br>Female ♀ Stressed Chrimson n = 6	**Sample Size (# cells)**<br>N= 484<br>N= 609<br>N= 672<br>N= 633	**Wilcoxon signed rank test (One Sample)**<br>Against 1.0<br>p = 0.235 , z = -1.188 r = -0.054<br>p = 0.008 , z = -2.640 r = -0.107<br>p = 0.492 , z = 0.687 r = 0.027<br>p = 0.006 , z = 2.742 r = 0.109
**Figure S3D**	**Shapiro-Wilk test**<br>Total interaction time<br>mCherry OFF p = 0.177<br>eNPHR OFF p = 0.521<br>mCherry ON p = 0.970<br>eNPHR ON p = 0.540<br><br>Number of visits<br>mCherry OFF p = 0.490<br>eNPHR OFF p = 0.065<br>mCherry ON p = 0.603<br>eNPHR ON p = 0.772	**Levene's test**<br>OFF p = 0.976<br>ON p = 0.591<br>Box's M test<br>p = 0.827<br><br>OFF p = 0.456<br>ON p = 0.856<br>Box's M test<br>p = 0.327	**Two-Way mixed ANOVA**<br>Interaction F(1,14) = 0.0909 , p = 0.768 , partial η^2 = 0.006<br>Light (W) F(1,14) = 2.4759 , p = 0.138 , partial η^2 = 0.150<br>Opsin (B) F(1,14) = 4.3559 , p = 0.056 , partial η^2 = 0.237<br>post hoc (Bonferroni)<br>mCherry ON x OFF p = 0.384<br>eNPHR ON x OFF p = 0.206<br><br>**Two-Way mixed ANOVA**<br>Interaction F(1,14) = 0.0020 , p = 0.965 , partial η^2 = 1E-04<br>Light (W) F(1,14) = 1.1265 , p = 0.306 , partial η^2 = 0.074<br>Opsin (B) F(1,14) = 0.2207 , p = 0.646 , partial η^2 = 0.016<br>post hoc (Bonferroni)<br>mCherry ON x OFF p = 0.484<br>eNPHR ON x OFF p = 0.447

	Interaction time per visit		**Two-Way mixed ANOVA**
	mCherry OFF p = 0.328	OFF p = 0.573	Interaction F(1,14) = 0.2059 , p = 0.657 , partial η^2 = 0.014
	eNPHR OFF p = 0.675	ON p = 0.100	Light (W) F(1,14) = 0.0000 , p = 0.999 , partial η^2 = <0.001
	mCherry ON p = 0.201		Opsin (B) F(1,14) = 5.5826 , p = 0.033 , partial η^2 = 0.285
	eNPHR ON p = 0.583		
		Box's M test	post hoc (Bonferroni)
		p = 0.357	mCherry ON x OFF p = 0.754
			eNPHR ON x OFF p = 0.752
	**Sample Size**		
	Female ♀ mCherry n = 8		
	Female ♀ eNPHR n = 8		
**Figure S3E**	**Shapiro-Wilk test**	**Levene's test**	**Two-Way mixed ANOVA**
	Total interaction time		
	tdTomato OFF p = 0.310	OFF p = 0.791	Interaction F(1,14) = 0.7861 , p = 0.390 , partial η^2 = 0.053
	Chrimson OFF p = 0.755	ON p = 0.734	Light (W) F(1,14) = 0.0000 , p = 0.998 , partial η^2 = <0.001
	tdTomato ON p = 0.116		Opsin (B) F(1,14) = 0.5529 , p = 0.469 , partial η^2 = 0.038
	Chrimson ON p = 0.894		
		Box's M test	post hoc (Bonferroni)
		p = 0.735	tdTomato ON x OFF p = 0.540
			Chrimson ON x OFF p = 0.542
	Number of visits		**Wilcoxon signed-rank test, two-sided**
	tdTomato OFF p = 0.285	OFF p = 0.916	tdTomato ON x OFF , p = 0.499 , z = 0.676
	Chrimson OFF p = 0.314	ON p = 0.380	r = 0.169
	tdTomato ON p = 0.008		Chrimson ON x OFF , p = 0.091 , z = 0.091
	Chrimson ON p = 0.198		r = 0.023
		Box's M test	
		p = 0.217	
	Interaction time per visit		**Two-Way mixed ANOVA**
	tdTomato OFF p = 0.336	OFF p = 0.885	Interaction F(1,14) = 2.8794 , p = 0.112 , partial η^2 = 0.171
	Chrimson OFF p = 0.719	ON p = 0.863	Light (W) F(1,14) = 0.6306 , p = 0.440 , partial η^2 = 0.043
	tdTomato ON p = 0.802		Opsin (B) F(1,14) = 0.1131 , p = 0.742 , partial η^2 = 0.008
	Chrimson ON p = 0.528		
		Box's M test	post hoc (Bonferroni)
		p = 0.870	tdTomato ON x OFF p = 0.534
			Chrimson ON x OFF p = 0.100
	**Sample Size**		
	Female ♀ tdTomato n = 8		
	Female ♀ Chrimson n = 8		
**Figure S3G**	**Shapiro-Wilk test**	**Levene's test**	**Two-Way ANOVA**
	Total interaction time		
	Unstressed p = 0.809	p = 0.662	Interaction F(3,32) = 37.3786 , p = <0.001 , partial η^2 = 0.572
	Unstressed + Opsin p = 0.749		Stress (B) F(3,32) = 0.0986 , p = 0.756 , partial η^2 = 0.004
	Stressed p = 0.753		Opsin+ (B) F(3,32) = 0.0246 , p = 0.876 , partial η^2 = <0.001
	Stressed + Opsin p = 0.622		
			post hoc (Bonferroni)
	**Sample Size**		Unstressed No Opsin x Opsin p = <0.001
	Female ♀ Unstressed n = 8		Stressed No Opsin x Opsin p = <0.001
	Female ♀ Unstressed + Opsin n = 8		
	Female ♀ Stressed n = 8		
	Female ♀ Stressed + Opsin n = 8		
**Figure S4A**	**Sample Size (# cells)**	**Expected Count <5**	**Chi-square test of homogeneity**
	Unstressed mCherry = 1165	0%	tdtomato x mCherry p = 0.758 $\chi^2(2)$ = 0.555
	Stressed tdTomato = 1196		Phi = 0.015
	**Sample Size (# animals)**		
	Female ♀ tdTomato n = 8		
	Female ♀ mCherry n = 8		
**Figure S4B**	**Sample Size (# cells)**	**Expected Count <5**	**Chi-square test of homogeneity**
	Unstressed mCherry = 1165	0%	tdtomato x mCherry p = 0.102 $\chi^2(2)$ = 4.568
	Stressed tdTomato = 1196		Phi = 0.044
	**Sample Size (# animals)**		
	Female ♀ tdTomato n = 8		
	Female ♀ mCherry n = 8		
**Figure S4C**	**Sample Size (# cells)**	**Expected Count <5**	**Chi-square test of homogeneity**
	Unstressed mCherry = 1165	0%	Unstressed mCherry ON x OFF p = 0.489
	Unstressed eNPHR = 1175		Unstressed eNPHR ON x OFF p = 0.206
	Stressed tdTomato = 1196		Stressed tdTomato ON x OFF p = 0.509
	Stressed Chrimson = 1087		Stressed Chrimson ON x OFF p = 0.451
	**Sample Size (# animals)**		
	Female ♀ Unstressed mCherry n = 8		
	Female ♀ Unstressed eNPHR n = 8		
	Female ♀ Stressed tdTomato n = 8		

	Female ♀ Stressed Chrimson n = 8		
**Figure S4D**	**Sample Size (# cells)**	**Expected Count <5**	**Chi-square test of homogeneity**
	Unstressed mCherry = 1165	0%	Unstressed mCherry ON x OFF p = 0.471
	Unstressed eNPHR = 1175		Unstressed eNPHR ON x OFF p = 0.916
	Stressed tdTomato = 1196		Stressed tdTomato ON x OFF p = 0.347
	Stressed Chrimson = 1087		Stressed Chrimson ON x OFF p = 0.517
	**Sample Size (# animals)**		
	Female ♀ Unstressed mCherry n = 8		
	Female ♀ Unstressed eNPHR n = 8		
	Female ♀ Stressed tdTomato n = 8		
	Female ♀ Stressed Chrimson n = 8		
**Figure S4E**	**Sample Size (# cells)**	**Expected Count <5**	**Chi-square test of homogeneity**
	Unstressed mCherry = 972	0%	Unstressed mCherry ON x OFF p = 0.306
	Unstressed eNPHR = 972		Unstressed eNPHR ON x OFF p = 0.697
	Stressed tdTomato = 945		Stressed tdTomato ON x OFF p = 0.606
	Stressed Chrimson = 1042		Stressed Chrimson ON x OFF p = 0.239
	**Sample Size (# animals)**		
	Female ♀ Unstressed mCherry n = 8		
	Female ♀ Unstressed eNPHR n = 8		
	Female ♀ Stressed tdTomato n = 8		
	Female ♀ Stressed Chrimson n = 8		
**Figure S5B**	**Shapiro-Wilk test**		**Paired Student t-Test, 2-tailed**
	Total interaction time p = 0.696		t (7) = 5.559 , p = 0.001 Cohen's d = 1.965
	Number of visits p = 0.462		t (7) = 2.457 , p = 0.044 Cohen's d = 0.869
	Interaction time per visit p = 0.914		t (7) = 2.811 , p = 0.026 Cohen's d = 0.994
	**Sample Size**		
	Female ♀ Virgin with SILA n = 8		
	Female ♀ Virgin without SILA n = 8		
**Figure S5C**	**Shapiro-Wilk test**		**Paired Student t-Test, 2-tailed**
	Total interaction time p = 0.733		t (7) = 3.570 , p = 0.009 Cohen's d = 1.262
	Number of visits p = 0.658		t (7) = 1.014 , p = 0.344 Cohen's d = 0.359
			**Wilcoxon signed-rank test, two-sided**
	Interaction time per visit p = 0.041		Novel x Familiar p = 0.017 z = 2.38
	**Sample Size**		r = 0.841
	Female ♀ Virgin with SILA n = 8		
	Female ♀ Virgin without SILA n = 8		
**Figure S5D**	**Sample Size (# cells)**	**Expected Count <5**	**Chi-square test of homogeneity**
	Virgin with SILA = 1085	0%	Empty cage Unstressed x Stressed p = 0.403
	Virgin without SILA = 1088		
	**Sample Size (# animals)**		Mouse cage Unstressed x Stressed p = 0.330
	Female ♀ Virgin with SILA n = 8		
	Female ♀ Virgin without SILA n = 8		
**Figure S5E**	**Sample Size (# cells)**	**Expected Count <5**	**Chi-square test of homogeneity**
	Virgin with SILA = 1086	0%	Familiar Unstressed x Stressed p = 0.708
	Virgin without SILA = 1076		
	**Sample Size (# animals)**		Novel Unstressed x Stressed p = 0.954
	Female ♀ Virgin with SILA n = 8		
	Female ♀ Virgin without SILA n = 8		
**Figure S6B**	**Shapiro-Wilk test**	**Levene's test**	**Student t-Test Independent samples, 2-tailed**
	Unstressed Vglut p = 0.941	Vglut p = 0.094	Vglut t (14) = -4.083 , p = 0.001
	Stressed Vglut p = 0.425		Cohen's d = -2.042
			**Welch t-Test Independent samples, 2-tailed**
	Unstressed Vgat p = 0.645	Vgat p = 0.046	Vgat t (14) = 0.978 , p = 0.349
	Stressed Vgat p = 0.336		Cohen's d = 0.489
	**Sample Size**		
	Female ♀ Unstressed Vglut n = 8		
	Female ♀ Stressed Vglut n = 8		
	Female ♀ Unstressed Vgat n = 8		
	Female ♀ Stressed Vgat n = 8		
**Figure S6D**	**Shapiro-Wilk test**	**Levene's test**	**Two-Way mixed ANOVA**
	Male ♂ Vglut p = 0.716	Vglut p = 0.811	Interaction F(1,14) = 1.4555 , p = 0.255 , partial η^2 = 0.127
	Female ♀ Vglut p = 0.140	Vgat p = 0.880	Marker (W) F(1,14) = 559.76 , p = <0.001 , partial η^2 = 0.982
	Male ♂ Vgat p = 0.396		Sex (B) F(1,14) = 1.3831 , p = 0.267 , partial η^2 = 0.122
	Female ♀ Vgat p = 0.936		
		Box's M test	post hoc (Bonferroni)
	**Sample Size**	p = 0.950	Male ♂ Vglut x Vgat p = <0.001
	Male ♂ Vglut n = 6		Female ♀ Vglut x Vgat p = <0.001

	Female ♀ Vglut *n* = 6		
	Male ♂ Vgat *n* = 6		
	Female ♀ Vgat *n* = 6		
**Figure**	**Shapiro-Wilk test**	**Levene's test**	**Two-Way mixed ANOVA**
**S6G**	Total interaction time		
	eNPHR OFF *p* = 0.562	OFF *p* = 0.981	Interaction F(1,14) = 0.0407 , *p* = 0.843 , partial η^2 = 0.003
	Chrimson OFF *p* = 0.782	ON *p* = 0.214	Light (W) F(1,14) = 0.4551 , *p* = 0.511 , partial η^2 = 0.031
	eNPHR ON *p* = 0.975		Opsin (B) F(1,14) = 0.1751 , *p* = 0.682 , partial η^2 = 0.012
	Chrimson ON *p* = 0.583		
		Box's M test	post hoc (Bonferroni)
		p = 0.465	eNPHR ON x OFF *p* = 0.743
			Chrimson ON x OFF *p* = 0.545
	Number of visits		**Wilcoxon Signed-rank test, two-sided**
	eNPHR OFF *p* = 0.499	OFF *p* = 0.244	eNPHR ON x OFF *p* = 0.779 z = -0.280
	Chrimson OFF *p* = 0.253	ON *p* = 0.030	*r* = -0.07
	eNPHR ON *p* = 0.714		Chrimson ON x OFF *p* = 0.735 z = -0.018
	Chrimson ON *p* = 0.304		*r* = -0.004
	Interaction time per visit		**Wilcoxon Signed-rank test, two-sided**
	eNPHR OFF *p* = 0.104	OFF *p* = 0.773	eNPHR ON x OFF *p* = 0.779 z = -0.280
	Chrimson OFF *p* = 0.815	ON *p* = 0.007	*r* = -0.07
	eNPHR ON *p* = 0.415		Chrimson ON x OFF *p* = 0.208 z = -1.26
	Chrimson ON *p* = 0.546		*r* = -0.315
	**Sample Size**		
	Female ♀ Unstressed eNPHR *n* = 8		
	Female ♀ Stressed Chrimson *n* = 8		
**Figure**	**Shapiro-Wilk test**	**Levene's test**	**Two-Way mixed ANOVA**
**S6H**	Total interaction time		
	eNPHR OFF *p* = 0.985	OFF *p* = 0.490	Interaction F(1,14) = 0.0024 , *p* = 0.962 , partial η^2 = <0.001
	Chrimson OFF *p* = 0.370	ON *p* = 0.849	Light (W) F(1,14) = 0.2363 , *p* = 0.634 , partial η^2 = 0.017
	eNPHR ON *p* = 0.865		Opsin (B) F(1,14) = 26.033 , *p* = <0.001 , partial η^2 = 0.650
	Chrimson ON *p* = 0.302		
		Box's M test	post hoc (Bonferroni)
		p = 0.428	eNPHR ON x OFF *p* = 0.711
			Chrimson ON x OFF *p* = 0.762
	Number of visits		**Two-Way mixed ANOVA**
	eNPHR OFF *p* = 0.661	OFF *p* = 0.097	Interaction F(1,14) = 0.015 , *p* = 0.904 , partial η^2 = 0.001
	Chrimson OFF *p* = 0.656	ON *p* = 0.543	Light (W) F(1,14) = 0.1738 , *p* = 0.683 , partial η^2 = 0.012
	eNPHR ON *p* = 0.132		Opsin (B) F(1,14) = 0.9663 , *p* = 0.342 , partial η^2 = 0.065
	Chrimson ON *p* = 0.566		
		Box's M test	post hoc (Bonferroni)
		p = 0.742	eNPHR ON x OFF *p* = 0.838
			Chrimson ON x OFF *p* = 0.709
	Interaction time per visit		**Two-Way mixed ANOVA**
	eNPHR OFF *p* = 0.526	OFF *p* = 0.577	Interaction F(1,14) = 0.0007 , *p* = 0.980 , partial η^2 = <0.001
	Chrimson OFF *p* = 0.237	ON *p* = 0.888	Light (W) F(1,14) = 0.3774 , *p* = 0.549 , partial η^2 = 0.026
	eNPHR ON *p* = 0.947		Opsin (B) F(1,14) = 20.889 , *p* = <0.001 , partial η^2 = 0.599
	Chrimson ON *p* = 0.922		
		Box's M test	post hoc (Bonferroni)
		p = 0.293	eNPHR ON x OFF *p* = 0.684
	**Sample Size**		Chrimson ON x OFF *p* = 0.658
	Female ♀ Unstressed eNPHR *n* = 8		
	Female ♀ Stressed Chrimson *n* = 8		
**Figure**	**Sample Size (# cells)**	**Expected Count <5**	**Chi-square test of homogeneity**
**S6I**	Unstressed eNPHR = 1032	0%	eNPHR Empty ON x OFF *p* = 0.965
	Stressed Chrimson = 1217		Chrimson Empty ON x OFF *p* = 0.175
	**Sample Size (# animals)**		eNPHR Mouse ON x OFF *p* = 0.692
	Female ♀ Unstressed eNPHR *n* = 8		Chrimson Mouse ON x OFF *p* = 0.509
	Female ♀ Stressed Chrimson *n* = 8		
**Figure**	**Sample Size (# cells)**	**Expected Count <5**	**Chi-square test of homogeneity**
**S6J**	Unstressed eNPHR = 1082	0%	eNPHR Familiar ON x OFF *p* = 0.865
	Stressed Chrimson = 1152		Chrimson Familiar ON x OFF *p* = 0.416
	**Sample Size (# animals)**		eNPHR Novel ON x OFF *p* = 0.936
	Female ♀ Unstressed eNPHR *n* = 8		Chrimson Novel ON x OFF *p* = 0.847
	Female ♀ Stressed Chrimson *n* = 8		
**Figure**	**Sample Size (# cells)**	**Expected Count <5**	**Chi-square test of homogeneity**
**S7**	Unstressed eNPHR = 972	0%	Chrimson x eNPHR *p* = 0.846

	Stressed Chrimson = 1042<br>**Sample Size (# animals)**<br>Female ♀ Unstressed eNPHR *n* = 8<br>Female ♀ Stressed Chrimson *n* = 8		
**Figure**<br>**S8A**	**Sample Size (# cells)**<br>Unstressed mCherry = 1165<br>Stressed tdTomato = 1196<br>Unstressed eNPHR = 1175<br>Stressed Chrimson = 1087<br>**Sample Size (# animals)**<br>Female ♀ Unstressed mCherry *n* = 8<br>Female ♀ Stressed tdTomato *n* = 8<br>Female ♀ Unstressed eNPHR *n* = 8<br>Female ♀ Stressed Chrimson *n* = 8	**Expected Count <5**<br>0%	**Chi-square test of homogeneity**<br>eNPHR x mCherry p = 0.658 $\chi^2(8) = 5.9$<br>Phi = 0.05<br><br>Chrimson x tdTomato p = 0.609 $\chi^2(8) = 6.342$<br>Phi = 0.053
**Figure**<br>**S8B**	**Sample Size (# cells)**<br>Unstressed mCherry = 1165<br>Stressed tdTomato = 1196<br>Unstressed eNPHR = 1175<br>Stressed Chrimson = 1087<br>**Sample Size (# animals)**<br>Female ♀ Unstressed mCherry *n* = 8<br>Female ♀ Stressed tdTomato *n* = 8<br>Female ♀ Unstressed eNPHR *n* = 8<br>Female ♀ Stressed Chrimson *n* = 8	**Expected Count <5**<br>0%	**Chi-square test of homogeneity**<br>eNPHR x mCherry p = 0.318 $\chi^2(8) = 9.291$<br>Phi = 0.063<br><br>Chrimson x tdTomato p = 0.304 $\chi^2(8) = 9.467$<br>Phi = 0.064
**Figure**<br>**S8C**	**Sample Size (# cells)**<br>Unstressed mCherry = 972<br>Stressed tdTomato = 945<br>Unstressed eNPHR = 972<br>Stressed Chrimson = 1042<br>**Sample Size (# animals)**<br>Female ♀ Unstressed mCherry *n* = 8<br>Female ♀ Stressed tdTomato *n* = 8<br>Female ♀ Unstressed eNPHR *n* = 8<br>Female ♀ Stressed Chrimson *n* = 8	**Expected Count <5**<br>0%	**Chi-square test of homogeneity**<br>eNPHR x mCherry p = 0.745 $\chi^2(8) = 5.12$<br>Phi = 0.051<br><br>Chrimson x tdTomato p = 0.165 $\chi^2(8) = 11.71$<br>Phi = 0.077
**Figure**<br>**S9B**	**Shapiro-Wilk test**<br>Total interaction time<br>mCherry OFF p = 0.877<br>eNPHR OFF p = 0.387<br>mCherry ON p = 0.729<br>eNPHR ON p = 0.700<br><br>Number of visits<br>mCherry OFF p = 0.633<br>eNPHR OFF p = 0.872<br>mCherry ON p = 0.889<br>eNPHR ON p = 0.506<br><br>Interaction time per visit<br>mCherry OFF p = 0.686<br>eNPHR OFF p = 0.706<br>mCherry ON p = 0.466<br>eNPHR ON p = 0.725<br>**Sample Size**<br>Male ♂ mCherry *n* = 8<br>Male ♂ eNPHR *n* = 8	**Levene's test**<br>OFF p = 0.433<br>ON p = 0.603<br>Box's M test<br>p = 0.702<br><br>OFF p = 0.0905<br>ON p = 0.9041<br>Box's M test<br>p = 0.3328<br><br>OFF p = 0.0182<br>ON p = 0.0853	**Two-Way mixed ANOVA**<br>Interaction $F(1,14)$ = 2.125 , p = 0.167 , partial η^2 = 0.132<br>Light (W) $F(1,14)$ = 0.066 , p = 0.801 , partial η^2 = 0.005<br>Opsin (B) $F(1,14)$ = 0.010 , p = 0.922 , partial η^2 = <0.001<br>post hoc (Bonferroni)<br>mCherry ON x OFF p = 0.410<br>eNPHR ON x OFF p = 0.245<br><br>**Two-Way mixed ANOVA**<br>Interaction $F(1,14)$ = 0.5086 , p = 0.487 , partial η^2 = 0.035<br>Light (W) $F(1,14)$ = 0.5361 , p = 0.476 , partial η^2 = 0.037<br>Opsin (B) $F(1,14)$ = 0.0255 , p = 0.875 , partial η^2 = 0.002<br>post hoc (Bonferroni)<br>mCherry ON x OFF p = 0.989<br>eNPHR ON x OFF p = 0.324<br><br>**Related Sampes Sign Test**<br>mCherry ON x OFF p = 0.289 z = -1.061<br>r = -0.265<br>eNPHR ON x OFF p = 1.000 z = 0.00<br>r = 0.00
**Figure**<br>**S9C**	**Shapiro-Wilk test**<br>Total interaction time<br>mCherry OFF p = 0.470<br>eNPHR OFF p = 0.879<br>mCherry ON p = 0.715<br>eNPHR ON p = 0.924	**Levene's test**<br>OFF p = 0.568<br>ON p = 0.110<br>Box's M test<br>p = 0.391	**Two-Way mixed ANOVA**<br>Interaction $F(1,14)$ = 1.794 , p = 0.202 , partial η^2 = 0.114<br>Light (W) $F(1,14)$ = 0.651 , p = 0.433 , partial η^2 = 0.044<br>Opsin (B) $F(1,14)$ = 0.015 , p = 0.903 , partial η^2 = 0.001<br>post hoc (Bonferroni)<br>mCherry ON x OFF p = 0.712<br>eNPHR ON x OFF p = 0.151

Figure	Shapiro-Wilk test	Levene's test	Statistical test
	Number of visits		**Two-Way mixed ANOVA**
	mCherry OFF *p* = 0.051	OFF *p* = 0.7447	Interaction F(1,14) = 1.2134 , *p* = 0.289 , partial η^2 = 0.080
	eNPHR OFF *p* = 0.209	ON *p* = 0.3767	Light (W) F(1,14) = 0.1482 , *p* = 0.706 , partial η^2 = 0.010
	mCherry ON *p* = 0.910		Opsin (B) F(1,14) = 0.241 , *p* = 0.631 , partial η^2 = 0.017
	eNPHR ON *p* = 0.677		
		Box's M test	post hoc (Bonferroni)
		p = 0.950	mCherry ON x OFF *p* = 0.311
			eNPHR ON x OFF *p* = 0.620
	Interaction time per visit		**Two-Way mixed ANOVA**
	mCherry OFF *p* = 0.756	OFF *p* = 0.1665	Interaction F(1,14) = 0.5804 , *p* = 0.459 , partial η^2 = 0.040
	eNPHR OFF *p* = 0.973	ON *p* = 0.3728	Light (W) F(1,14) = 2.9871 , *p* = 0.106 , partial η^2 = 0.176
	mCherry ON *p* = 0.302		Opsin (B) F(1,14) = 0.7484 , *p* = 0.402 , partial η^2 = 0.051
	eNPHR ON *p* = 0.190		
		Box's M test	post hoc (Bonferroni)
	**Sample Size**	*p* = 0.0446	mCherry ON x OFF *p* = 0.506
	Female ♀ mCherry *n* = 8		eNPHR ON x OFF *p* = 0.100
	Female ♀ eNPHR *n* = 8		
**Figure S9D**	**Shapiro-Wilk test**	**Levene's test**	**Two-Way mixed ANOVA**
	Total interaction time		
	mCherry OFF *p* = 0.723	OFF *p* = 0.606	Interaction F(1,14) = 11.390 , *p* = 0.005 , partial η^2 = 0.449
	eNPHR OFF *p* = 0.487	ON *p* = 0.642	Light (W) F(1,14) = 2.045 , *p* = 0.175 , partial η^2 = 0.127
	mCherry ON *p* = 0.707		Opsin (B) F(1,14) = 10.686 , *p* = 0.006 , partial η^2 = 0.433
	eNPHR ON *p* = 0.274		
		Box's M test	post hoc (Bonferroni)
		p = 0.431	mCherry ON x OFF *p* = 0.191
			eNPHR ON x OFF *p* = 0.004
	Number of visits		**Wilcoxon signed-rank test, two-sided**
	mCherry OFF *p* = 0.014	OFF *p* = 0.514	mCherry ON x OFF *p* = 0.161 *z* = 1.400
	eNPHR OFF *p* = 0.883	ON *p* = 0.7806	*r* = 0.495
	mCherry ON *p* = 0.155		eNPHR ON x OFF *p* = 0.674 *z* = 0.420
	eNPHR ON *p* = 0.689		*r* = 0.148
	Interaction time per visit		**Two-Way mixed ANOVA**
	mCherry Light OFF p = 0.051	Empty p = 0.249	Interaction F(1,14) = 4.518 , p = 0.052 , partial η^2 = 0.244
	eNPHR Light OFF p = 0.817	Mouse p = 0.725	Light (W) F(1,14) = 2.983 , p = 0.106 , partial η^2 = 0.176
	mCherry Light ON p = 0.952		Opsin (B) F(1,14) = 4.782 , p = 0.046 , partial η^2 = 0.255
	eNPHR Light ON p = 0.121		
	**Sample Size**	Box's M test	post hoc (Bonferroni)
	Male ♂ mCherry *n* = 8	p = 0.907	mCherry ON x OFF p = 0.782
	Male ♂ eNPHR *n* = 8		eNPHR ON x OFF p = 0.016
**Figure S9E**	**Shapiro-Wilk test**	**Levene's test**	**Wilcoxon signed-rank test, two-sided**
	Total interaction time		
	mCherry OFF *p* = 0.024	OFF *p* = 0.200	mCherry ON x OFF *p* = 0.263 *z* = 1.120
	eNPHR OFF *p* = 0.335	ON *p* = 0.745	*r* = 0.396
	mCherry ON *p* = 0.007		eNPHR ON x OFF *p* = 0.017 *z* = -2.380
	eNPHR ON *p* = 0.476		*r* = -0.841
	Number of visits		**Wilcoxon signed-rank test, two-sided**
	mCherry OFF *p* = 0.195	OFF *p* = 0.351	mCherry ON x OFF *p* = 0.484 *z* = 0.700
	eNPHR OFF *p* = 0.491	ON *p* = 0.253	*r* = 0.247
	mCherry ON *p* = 0.042		eNPHR ON x OFF *p* = 0.674 *z* = -0.420
	eNPHR ON *p* = 0.622		*r* = -0.148
	Interaction time per visit		**Two-Way mixed ANOVA**
	mCherry OFF *p* = 0.863	Empty *p* = 0.280	Interaction F(1,14) = 21.425 , *p* = <0.001 , partial η^2 = 0.605
	eNPHR OFF *p* = 0.329	Mouse *p* = 0.928	Light (W) F(1,14) = 13.94 , *p* = 0.002 , partial η^2 = 0.499
	mCherry ON *p* = 0.560		Opsin (B) F(1,14) = 3.11 , *p* = 0.100 , partial η^2 = 0.182
	eNPHR ON *p* = 0.668		
	**Sample Size**	Box's M test	post hoc (Bonferroni)
	Female ♀ mCherry *n* = 8	*p* = 0.7559	mCherry ON x OFF *p* = 0.537
	Female ♀ eNPHR *n* = 8		eNPHR ON x OFF *p* = <0.001
**Figure S10A**	**Sample Size (# cells)**	**Expected Count <5**	**Chi-square test of homogeneity**
	Virgin ♂ mCherry = 1060	0%	Virgin Male ♂ mCherry ON x OFF *p* = 0.532
	Virgin ♂ eNPHR = 1052		Virgin Male ♂ eNPHR ON x OFF *p* = 0.623
	Virgin ♀ mCherry = 1088		Virgin Female ♀ mCherry ON x OFF *p* = 0.248
	Virgin ♀ eNPHR = 1113		Virgin Female ♀ eNPHR ON x OFF *p* = 0.150

	**Sample Size (# animals)**<br>Virgin ♂ mCherry *n* = 8<br>Virgin ♂ eNPHR *n* = 8<br>Virgin ♀ mCherry *n* = 8<br>Virgin ♀ eNPHR *n* = 8		
**Figure S10B**	**Sample Size (# cells)**<br>Virgin ♂ mCherry = 1060<br>Virgin ♂ eNPHR = 1052<br>Virgin ♀ mCherry = 1088<br>Virgin ♀ eNPHR = 1113<br>**Sample Size (# animals)**<br>Virgin ♂ mCherry *n* = 8<br>Virgin ♂ eNPHR *n* = 8<br>Virgin ♀ mCherry *n* = 8<br>Virgin ♀ eNPHR *n* = 8	**Expected Count <5**<br>0%	**Chi-square test of homogeneity**<br>Virgin Male ♂ mCherry ON x OFF *p* = 0.632<br>Virgin Male ♂ eNPHR ON x OFF *p* = 0.923<br>Virgin Female ♀ mCherry ON x OFF *p* = 0.400<br>Virgin Female ♀ eNPHR ON x OFF *p* = 0.587
**Figure S10C**	**Sample Size (# cells)**<br>Virgin ♂ mCherry = 1167<br>Virgin ♂ eNPHR = 1095<br>Virgin ♀ mCherry = 1076<br>Virgin ♀ eNPHR = 1067<br>**Sample Size (# animals)**<br>Virgin ♂ mCherry *n* = 8<br>Virgin ♂ eNPHR *n* = 8<br>Virgin ♀ mCherry *n* = 8<br>Virgin ♀ eNPHR *n* = 8	**Expected Count <5**<br>0%	**Chi-square test of homogeneity**<br>Virgin Male ♂ mCherry ON x OFF *p* = 0.448<br>Virgin Male ♂ eNPHR ON x OFF *p* = 0.136<br>Virgin Female ♀ mCherry ON x OFF *p* = 0.170<br>Virgin Female ♀ eNPHR ON x OFF *p* = 0.452
**Figure S10D**	**Sample Size (# cells)**<br>Virgin ♂ mCherry = 1167<br>Virgin ♂ eNPHR = 1095<br>Virgin ♀ mCherry = 1076<br>Virgin ♀ eNPHR = 1067<br>**Sample Size (# animals)**<br>Virgin ♂ mCherry *n* = 8<br>Virgin ♂ eNPHR *n* = 8<br>Virgin ♀ mCherry *n* = 8<br>Virgin ♀ eNPHR *n* = 8	**Expected Count <5**<br>0%	**Chi-square test of homogeneity**<br>Virgin Male ♂ mCherry ON x OFF *p* = 0.625<br>Virgin Male ♂ eNPHR ON x OFF *p* = <0.001<br>Virgin Female ♀ mCherry ON x OFF *p* = 0.267<br>Virgin Female ♀ eNPHR ON x OFF *p* = <0.001
**Figure S11A**	**Sample Size (# cells)**<br>Unstressed mCherry = 1167<br>Unstressed eNPHR = 1095<br>**Sample Size (# animals)**<br>Male ♂ Unstressed mCherry *n* = 8<br>Male ♂ Unstressed eNPHR *n* = 8	**Expected Count <5**<br>0%	**Chi-square test of homogeneity**<br>eNPHR x mCherry *p* = <0.001 $\chi^2(8)$ = 32.28<br>Phi = 0.119
**Figure S11B**	**Sample Size (# cells)**<br>Unstressed mCherry = 1076<br>Unstressed eNPHR = 1067<br>**Sample Size (# animals)**<br>Female ♀ Unstressed mCherry *n* = 8<br>Female ♀ Unstressed eNPHR *n* = 8	**Expected Count <5**<br>0%	**Chi-square test of homogeneity**<br>eNPHR x mCherry *p* = <0.001 $\chi^2(8)$ = 26.87<br>Phi = 0.112
**Figure S12B**	**Shapiro-Wilk test**<br>Discrimination Index<br>Unstressed Opsin+ OFF *p* = 0.092<br>Unstressed Opsin+ ON *p* = 0.591<br>Unstressed Opsin- OFF *p* = 0.756<br>Unstressed Opsin- ON *p* = 0.461<br>Stressed Opsin+ OFF *p* = 0.807<br>Stressed Opsin+ ON *p* = 0.485<br>Stressed Opsin- OFF *p* = 0.275<br>Stressed Opsin- ON *p* = 0.307<br>**Sample Size**<br>Female ♀ Unstressed Opsin+ *n* = 8<br>Female ♀ Unstressed Opsin- *n* = 8<br>Female ♀ Stressed Opsin+ *n* = 8<br>Female ♀ Stressed Opsin- *n* = 8<br>Unstressed Opsin+ = eNPHR<br>Stressed Opsin+ = Chrimson	**Levene's test**<br>OFF *p* = 0.543<br>ON *p* = 0.184<br>Box's M test<br>*p* = 0.264	**Three-Way mixed ANOVA**<br>Interaction F(1,28) = 0.102 , *p* = 0.752 , partial η^2 = 0.004<br>Light (W) F(1,28) = 0.002 , *p* = 0.969 , partial η^2 = <0.001<br>Stress (B) F(1,28) = 0.069 , *p* = 0.795 , partial η^2 = 0.002<br>Opsin (B) F(1,28) = 2.683 , *p* = 0.113 , partial η^2 = 0.087<br>post hoc (Bonferroni)<br>Unstressed Opsin+ OFF x ON *p* = 0.579<br>Unstressed Opsin- OFF x ON *p* = 0.752<br>Stressed Opsin+ OFF x ON *p* = 0.504<br>Stressed Opsin- OFF x ON *p* = 0.408
**Figure S12C**	**Sample Size (# cells)**<br>Unstressed mCherry = 976<br>Unstressed eNPHR = 1013	**Expected Count <5**<br>0%	**Chi-square test of homogeneity**<br>Unstressed mCherry ON x OFF *p* = 0.755<br>Unstressed eNPHR ON x OFF *p* = 0.408

	Stressed tdTomato = 1007<br>Stressed Chrimson = 1030<br><br>**Sample Size (# animals)**<br>Female ♀ Unstressed mCherry *n* = 8<br>Female ♀ Unstressed eNPHR *n* = 8<br>Female ♀ Stressed tdTomato *n* = 8<br>Female ♀ Stressed Chrimson *n* = 8		Stressed tdTomato ON x OFF *p* = 0.824<br>Stressed Chrimson ON x OFF *p* = 0.475
**Figure S12D**	**Sample Size (# cells)**<br>Unstressed mCherry = 976<br>Unstressed eNPHR = 1013<br>Stressed tdTomato = 1007<br>Stressed Chrimson = 1030<br>**Sample Size (# animals)**<br>Female ♀ Unstressed mCherry *n* = 8<br>Female ♀ Unstressed eNPHR *n* = 8<br>Female ♀ Stressed tdTomato *n* = 8<br>Female ♀ Stressed Chrimson *n* = 8	**Expected Count <5**<br>0%	**Chi-square test of homogeneity**<br>Unstressed mCherry ON x OFF *p* = 0.795<br>Unstressed eNPHR ON x OFF *p* = 0.296<br>Stressed tdTomato ON x OFF *p* = 0.563<br>Stressed Chrimson ON x OFF *p* = 0.704
**Figure S12F**	**Shapiro-Wilk test**<br>Total exploration time<br>Unstressed *p* = 0.449<br>Stressed *p* = 0.797<br><br>Number of visits<br>Unstressed *p* = 0.674<br>Stressed *p* = 0.843<br><br>Exploration time per visit<br>Unstressed *p* = 0.253<br>Stressed *p* = 0.768<br>**Sample Size**<br>Female ♀ Unstressed *n* = 8<br>Female ♀ Stressed *n* = 8	**Levene's test**<br><br>*p* = 0.331<br><br><br><br>*p* = 0.163<br><br><br><br>*p* = 0.494	**Student t-Test Independent samples, 2-tailed**<br><br>*t* (14) = -1.057 , *p* = 0.308<br>Cohen's d = -0.529<br><br>**Student t-Test Independent samples, 2-tailed**<br>*t* (14) = -0.045 , *p* = 0.965<br>Cohen's d = -0.023<br><br>**Student t-Test Independent samples, 2-tailed**<br>*t* (14) = -0.843 , *p* = 0.414<br>Cohen's d = -0.421
**Figure S12H**	**Shapiro-Wilk test**<br>Total exploration time<br>Unstressed *p* = 0.085<br>Stressed *p* = 0.665<br><br>Number of visits<br>Unstressed *p* = 0.301<br>Stressed *p* = 0.962<br><br>Exploration time per visit<br>Unstressed *p* = 0.135<br>Stressed *p* = 0.024<br>**Sample Size**<br>Female ♀ Unstressed *n* = 8<br>Female ♀ Stressed *n* = 8	**Levene's test**<br><br>*p* = 0.006<br><br><br><br>*p* = 0.285<br><br><br><br>*p* = 0.328	**Welch t-Test Independent samples, 2-tailed**<br><br>*t* (14) = 0.719 , *p* = 0.487<br>Cohen's d = 0.360<br><br>**Student t-Test Independent samples, 2-tailed**<br>*t* (14) = -0.006 , *p* = 0.995<br>Cohen's d = -0.003<br><br>**Mann Whitney U test**<br>Unstressed x Stressed *p* = 0.130 *U* = 17.00<br>*r* = -0.394 *z* = -1.58
**Figure S12I**	**Shapiro-Wilk test**<br>Total exploration time<br>Unstressed *p* = 0.800<br>Stressed *p* = 0.966<br><br>Number of visits<br>Unstressed *p* = 0.409<br>Stressed *p* = 0.901<br><br>Exploration time per visit<br>Unstressed *p* = 0.512<br>Stressed *p* = 0.589<br>**Sample Size**<br>Female ♀ Unstressed *n* = 8<br>Female ♀ Stressed *n* = 8	**Levene's test**<br><br>*p* = 0.704<br><br><br><br>*p* = 0.118<br><br><br><br>*p* = 0.349	**Student t-Test Independent samples, 2-tailed**<br><br>*t* (14) = 0.221 , *p* = 0.828<br>Cohen's d = 0.111<br><br>**Student t-Test Independent samples, 2-tailed**<br>*t* (14) = 1.715 , *p* = 0.108<br>Cohen's d = 0.857<br><br>**Student t-Test Independent samples, 2-tailed**<br>*t* (14) = -1.284 , *p* = 0.220<br>Cohen's d = -0.642
**Figure S12K**	**Shapiro-Wilk test**<br>Time<br>Unstressed OFF *p* = 0.268<br>Stressed OFF *p* = 0.372<br>Unstressed ON *p* = 0.090<br>Stressed ON *p* = 0.482	**Levene's test**<br><br>OFF *p* = 0.858<br>ON *p* = 0.642<br><br><br>Box's M test	**Two-Way mixed ANOVA**<br><br>Interaction F(1,14) = 0.554 , *p* = 0.469 , partial η^2 = 0.038<br>Time (W) F(1,14) = 0.378 , *p* = 0.549 , partial η^2 = 0.026<br>Stress (B) F(1,14) = 0.785 , *p* = 0.391 , partial η^2 = 0.053<br><br><br>post hoc (Bonferroni)

		p = 0.097	Unstressed Empty x Mouse p = 0.353
	**Sample Size**		Stressed Empty x Mouse p = 0.928
	Female ♀ Unstressed n = 8		
	Female ♀ Stressed n = 8		
**Figure**	**Shapiro-Wilk test**	**Levene's test**	**Wilcoxon signed-rank test, two-sided**
**S13B**	Total interaction time		
	Exploration OFF p = 0.633	OFF p = 0.931	Exploration ON x OFF p = 0.779 z = 0.280
	Interaction OFF p = 0.047	ON p = 0.045	r = 0.07
	Exploration ON p = 0.173		Interaction ON x OFF p = 0.674 z = 0.420
	Interaction ON p = 0.334		r = 0.105
	Number of visits		**Wilcoxon signed-rank test, two-sided**
	Exploration OFF p = 0.092	OFF p = 0.029	Exploration ON x OFF p = 0.735 z = -0.338
	Interaction OFF p = 0.138	ON p = 0.349	r = -0.085
	Exploration ON p = 0.498		Interaction ON x OFF p = 0.779 z = -0.280
	Interaction ON p = 0.242		r = -0.07
	Interaction time per visit		**Two-Way mixed ANOVA**
	Exploration OFF p = 0.199	OFF p = 0.4668	Interaction $F(1,14)$ = 0.1306 , p = 0.723 , partial η^2 = 0.009
	Interaction OFF p = 0.653	ON p = 0.4076	Light (W) $F(1,14)$ = 0.3935 , p = 0.541 , partial η^2 = 0.027
	Exploration ON p = 0.839		Condition (B) $F(1,14)$ = 1.4447 , p = 0.249 , partial η^2 = 0.094
	Interaction ON p = 0.594		
		Box's M test	post hoc (Bonferroni)
	**Sample Size**	p = 0.851	Exploration ON x OFF p = 0.496
	Female ♀ Exploration n = 8		Interaction ON x OFF p = 0.854
	Female ♀ Interaction n = 8		
**Figure**	**Shapiro-Wilk test**	**Levene's test**	**Two-Way mixed ANOVA**
**S13C**	Total interaction time		
	Exploration OFF p = 0.854	OFF p = 0.529	Interaction $F(1,14)$ = 0.004 , p = 0.952 , partial η^2 = <0.001
	Interaction OFF p = 0.442	ON p = 0.866	Light (W) $F(1,14)$ = 0.010 , p = 0.921 , partial η^2 = <0.001
	Exploration ON p = 0.407		Condition (B) $F(1,14)$ = 0.037 , p = 0.851 , partial η^2 = 0.003
	Interaction ON p = 0.648		
		Box's M test	post hoc (Bonferroni)
		p = 0.8334	Exploration ON x OFF p = 0.978
			Interaction ON x OFF p = 0.910
	Number of visits		**Two-Way mixed ANOVA**
	Exploration OFF p = 0.141	OFF p = 0.9248	Interaction $F(1,14)$ = 0.7609 , p = 0.398 , partial η^2 = 0.052
	Interaction OFF p = 0.453	ON p = 0.5774	Light (W) $F(1,14)$ = 0.0016 , p = 0.968 , partial η^2 = <0.001
	Exploration ON p = 0.191		Condition (B) $F(1,14)$ = 0.1818 , p = 0.676 , partial η^2 = 0.013
	Interaction ON p = 0.910		
		Box's M test	post hoc (Bonferroni)
		p = 0.6557	Exploration ON x OFF p = 0.566
			Interaction ON x OFF p = 0.529
	Interaction time per visit		**Two-Way mixed ANOVA**
	Exploration OFF p = 0.240	OFF p = 0.4273	Interaction $F(1,14)$ = 1.4946 , p = 0.242 , partial η^2 = 0.096
	Interaction OFF p = 0.509	ON p = 0.1974	Light (W) $F(1,14)$ = 0.0029 , p = 0.958 , partial η^2 = <0.001
	Exploration ON p = 0.578		Condition (B) $F(1,14)$ = 0.2148 , p = 0.650 , partial η^2 = 0.015
	Interaction ON p = 0.068		
		Box's M test	post hoc (Bonferroni)
		p = 0.8284	Exploration ON x OFF p = 0.423
	**Sample Size**		Interaction ON x OFF p = 0.382
	Female ♀ Exploration n = 8		
	Female ♀ Interaction n = 8		
**Figure**	**Sample Size (# cells)**	**Expected Count <5**	**Chi-square test of homogeneity**
**S13D**	Exploration eNPHR = 978	0%	Exploration eNPHR ON x OFF p = 0.836
	Interaction eNPHR = 1076		Interaction eNPHR ON x OFF p = 0.479
	Exploration Chrimson = 981		Exploration Chrimson ON x OFF p = 0.402
	Interaction Chrimson = 1007		Interaction Chrimson ON x OFF p = 0.884
	**Sample Size (# animals)**		
	Female ♀ Exploration eNPHR n = 8		
	Female ♀ Interaction eNPHR n = 8		
	Female ♀ Exploration Chrimson n = 8		
	Female ♀ Interaction Chrimson n = 8		
**Figure**	**Sample Size (# cells)**	**Expected Count <5**	**Chi-square test of homogeneity**
**S13E**	Exploration eNPHR = 978	0%	Exploration eNPHR ON x OFF p = 0.525
	Interaction eNPHR = 1076		Interaction eNPHR ON x OFF p = 0.829
	Exploration Chrimson = 981		Exploration Chrimson ON x OFF p = 0.507
	Interaction Chrimson = 1007		Interaction Chrimson ON x OFF p = 0.976
	**Sample Size (# animals)**		
	Female ♀ Exploration eNPHR n = 8		

	Female ♀ Interaction eNPHR n = 8<br>Female ♀ Exploration Chrimson n = 8<br>Female ♀ Interaction Chrimson n = 8		
**Figure S13F**	**Sample Size (# cells)**<br>Exploration eNPHR = 1021<br>Interaction eNPHR = 1111<br>Exploration Chrimson = 997<br>Interaction Chrimson = 998<br>**Sample Size (# animals)**<br>Female ♀ Exploration eNPHR n = 8<br>Female ♀ Interaction eNPHR n = 8<br>Female ♀ Exploration Chrimson n = 8<br>Female ♀ Interaction Chrimson n = 8	**Expected Count <5**<br>0%	**Chi-square test of homogeneity**<br>Exploration eNPHR ON x OFF p = 0.339<br>Interaction eNPHR ON x OFF p = 0.127<br>Exploration Chrimson ON x OFF p = 0.752<br>Interaction Chrimson ON x OFF p = 0.162
**Figure S13G**	**Sample Size (# cells)**<br>Interaction eNPHR = 1111<br>Interaction Chrimson = 998<br>**Sample Size (# animals)**<br>Female ♀ Interaction eNPHR n = 8<br>Female ♀ Interaction Chrimson n = 8	**Expected Count <5**<br>0%	**Chi-square test of homogeneity**<br>eNPHR x Chrimson p = 0.485
**Figure S14B**	**Shapiro-Wilk test**<br>Unstressed AI p = 0.337<br>Stressed AI p = 0.219<br>Unstressed PrL p = 0.878<br>Stressed PrL p = 0.839<br>**Sample Size**<br>Female ♀ Unstressed n = 6<br>Female ♀ Stressed n = 6	**Levene's test**<br>Empty p = 0.8232<br>Mouse p = 0.2037<br>Box's M test<br>p = 0.355	**Two-Way mixed ANOVA**<br>Interaction F(1,14) = 0.0038 , p = 0.952 , partial η^2 = <0.001<br>Region (W) F(1,14) = 27.355 , p = <0.001 , partial η^2 = 0.732<br>Stress (B) F(1,14) = 0.0445 , p = 0.837 , partial η^2 = 0.004<br>post hoc (Bonferroni)<br>AI Unstressed x Stressed p = 0.916<br>PrL Unstressed x Stressed p = 0.811
**Figure S15C**	**Sample Size (# cells)**<br>n = 503<br>**Sample Size (# animals)**<br>Female ♀ B6J mice n = 4	**NA**	**NA**
**Figure S15F**	**Sample Size (# cells)**<br>n = 2007<br>**Sample Size (# animals)**<br>Female ♀ B6J mice n = 4	**NA**	**NA**
**Figure S16C**	**Sample Size (# cells)**<br>n = 658<br>**Sample Size (# animals)**<br>Female ♀ B6J mice n = 4	**NA**	**NA**
**Figure S16D**	**Shapiro-Wilk test**<br>Total interaction time<br>Unstressed Control p = 0.838<br>Unstressed GR-KO p = 0.638<br>Stressed Control p = 0.808<br>Stressed GR-KO p = 0.638<br>Number of visits<br>Unstressed Control p = 0.022<br>Unstressed GR-KO p = 0.939<br>Stressed Control p = 0.087<br>Stressed GR-KO p = 0.491<br>Interaction time per visit<br>Unstressed Control p = 0.029<br>Unstressed GR-KO p = 0.557<br>Stressed Control p = 0.418<br>Stressed GR-KO p = 0.106<br>**Sample Size**<br>Female ♀ Unstressed Control n = 6<br>Female ♀ Unstressed GR-KO n = 6<br>Female ♀ Stressed Control n = 6<br>Female ♀ Stressed GR-KO n = 6	**Levene's test**<br>p = 0.713<br>p = 0.6773<br>p = 0.4075	**Two-Way ANOVA**<br>Interaction F(3,20) = 0.792 , p = 0.384 , partial η^2 = 0.038<br>Stress (B) F(3,20) = 0.009 , p = 0.927 , partial η^2 = <0.001<br>KO (B) F(3,20) = 0.741 , p = 0.400 , partial η^2 = 0.036<br>post hoc (Bonferroni)<br>Unstressed Control x GR-KO p = 0.230<br>Stressed Control x GR-KO p = 0.984<br>**Mann Whitney U test**<br>Unstressed Control x GR-KO p = 0.699 U = 15.5<br>r = -0.1 z = -0.402<br>Stressed Control x GR-KO p = 1.000 U = 18.5<br>r = 0.02 z = 0.08<br>**Mann Whitney U test**<br>Unstressed Control x GR-KO p = 0.065 U = 6<br>r = -0.48 z = -1.922<br>Stressed Control x GR-KO p = 0.699 U = 21<br>r = 0.12 z = 0.48
