# Supplementary material for: Adolescent stress impairs postpartum social behavior via anterior insula-prelimbic pathway in mice
Source: Nat Commun. 2023 May 23;14:2975. doi: 10.1038/s41467-023-38799-6 (PMC10205810; doi:10.1038/s41467-023-38799-6)
Supplement: Supplementary file 2 — Reporting Summary [file 41467_2023_38799_MOESM2_ESM.pdf]

nature portfolio

Corresponding author(s): Minae Niwa

Last updated by author(s): May 12, 2023

Reporting Summary

Nature Portfolio wishes to improve the reproducibility of the work that we publish. This form provides structure for consistency and transparency in reporting. For further information on Nature Portfolio policies, see our Editorial Policies and the Editorial Policy Checklist.

Statistics

For all statistical analyses, confirm that the following items are present in the figure legend, table legend, main text, or Methods section.

n/a	Confirmed	
☐	☒	The exact sample size (*n*) for each experimental group/condition, given as a discrete number and unit of measurement
☐	☒	A statement on whether measurements were taken from distinct samples or whether the same sample was measured repeatedly
☐	☒	The statistical test(s) used AND whether they are one- or two-sided<br>*Only common tests should be described solely by name; describe more complex techniques in the Methods section.*
☒	☐	A description of all covariates tested
☐	☒	A description of any assumptions or corrections, such as tests of normality and adjustment for multiple comparisons
☐	☒	A full description of the statistical parameters including central tendency (e.g. means) or other basic estimates (e.g. regression coefficient) AND variation (e.g. standard deviation) or associated estimates of uncertainty (e.g. confidence intervals)
☐	☒	For null hypothesis testing, the test statistic (e.g. *F*, *t*, *r*) with confidence intervals, effect sizes, degrees of freedom and *P* value noted<br>*Give P values as exact values whenever suitable.*
☒	☐	For Bayesian analysis, information on the choice of priors and Markov chain Monte Carlo settings
☒	☐	For hierarchical and complex designs, identification of the appropriate level for tests and full reporting of outcomes
☐	☒	Estimates of effect sizes (e.g. Cohen's *d*, Pearson's *r*), indicating how they were calculated

Our web collection on statistics for biologists contains articles on many of the points above.

Software and code

Policy information about availability of computer code

Data collection	The Ethovision XT15 (Noldus) was used to record behavioral expereiments. Inscopix's nVOKE 2.0 was used for in vivo microendoscopic calcium imaging. Confocal imaging were acquired with a Zeiss LSM-800 Airyscan confocal microscope.
Data analysis	The following software packages were used for data analysis: Ethovision XT15 (Noldus), Inscopix Data Processing software (Inscopix), MATLAB 2020a, 2020b, and 2021a (MathWOrks), Image J (NIH), and SPSS 28 (IBM).

For manuscripts utilizing custom algorithms or software that are central to the research but not yet described in published literature, software must be made available to editors and reviewers. We strongly encourage code deposition in a community repository (e.g. GitHub). See the Nature Portfolio guidelines for submitting code & software for further information.

Data

Policy information about availability of data

All manuscripts must include a data availability statement. This statement should provide the following information, where applicable:

- Accession codes, unique identifiers, or web links for publicly available datasets
- A description of any restrictions on data availability
- For clinical datasets or third party data, please ensure that the statement adheres to our policy

The data generated in this study are provided in the Source Data file.

Human research participants

Policy information about studies involving human research participants and Sex and Gender in Research.

Reporting on sex and gender	N/A
Population characteristics	N/A
Recruitment	N/A
Ethics oversight	N/A

Note that full information on the approval of the study protocol must also be provided in the manuscript.

Field-specific reporting

Please select the one below that is the best fit for your research. If you are not sure, read the appropriate sections before making your selection.

☒ Life sciences ☐ Behavioural & social sciences ☐ Ecological, evolutionary & environmental sciences

For a reference copy of the document with all sections, see nature.com/documents/nr-reporting-summary-flat.pdf

Life sciences study design

All studies must disclose on these points even when the disclosure is negative.

Sample size	Our study did not use statistical methods to pre-determine the sample size. However, we chose the sample size that is comparable to previous studies (References 26, 27, and 38).
Data exclusions	To ensure data quality, we excluded data from mice that did not accurately receive viral injections or lens implantations, as described the Methods section.
Replication	All behavioral, imaging, optogenetics, and histology experiments were replicated in multiple subject animals independently at least three times, and similar results were obtained in each experiment. Data evaluation was not conducted independently across each cohort.
Randomization	To minimize bias, animals were assigned to groups randomly, with an effort to acheive comparable sample sizes across all groups.
Blinding	Blinding of the experimenters to group allocation was not implemented during data acquisition and analysis. To minimize potential bias, control and experimental groups were tested under the same experimental conditions when necessary, and data from all groups were analyzed using identical criteria and methods.

Reporting for specific materials, systems and methods

We require information from authors about some types of materials, experimental systems and methods used in many studies. Here, indicate whether each material, system or method listed is relevant to your study. If you are not sure if a list item applies to your research, read the appropriate section before selecting a response.

Materials & experimental systems

n/a	Involved in the study	
☐	☒	Antibodies
☒	☐	Eukaryotic cell lines
☒	☐	Palaeontology and archaeology
☐	☒	Animals and other organisms
☒	☐	Clinical data
☒	☐	Dual use research of concern

Methods

n/a	Involved in the study	
☒	☐	ChIP-seq
☒	☐	Flow cytometry
☒	☐	MRI-based neuroimaging

Antibodies

Antibodies used	Rabbit anti-Vglut1 (1:200, Abcam, ab227805), rabbit anti-Vgat (1:200, Millipore, AB5062P), rabbit anti-cFos (1:1000, Cell Signaling Technology, 2250), mouse anti-cFos (1:1000, Abcam, ab208942), goat anti-mouse F(ab) fragment (1:1000, Abcam, ab6668), Alexa

	Fluor 488 goat anti-rabbit (1:400, Invitrogen, A11008), Alexa Fluor 568 goat anti-rabbit (1:400, goat, Invitrogen, A11011), Alexa Fluor 488 goat anti-mouse (1:400, Invitrogen, A11001).
Validation	All primary antibodies used in this study were purchased from commercial companies and have been validated by the companies. The antibodies have also been used in previous publications (Jin et al, PLoS Biol 2021; Rossi et al, Nat Neurosci 2016; Gulmez Karaca et al, Nat Commun 2020; Grabrucker et al, EMBO J 2021; Xue et al, Sci Adv 2022).

Animals and other research organisms

Policy information about studies involving animals; ARRIVE guidelines recommended for reporting animal research, and Sex and Gender in Research

Laboratory animals	This study used several stains of mice, including C57BL/6J, Slc17a7 (Vglu1)-Cre, Ai14, and GR-flox, all of which were purchased from Jackson Laboratories (stock No: 000664, 023527, 007914, and 021021) and backcrossed to B6. Virus injections were administered to mice either 5 weeks or 7-10 weeks of age. To conduct the behavioral, imaging, optogenetic, and histology experiments, mice were used at postpartum days 7-17 (equivalent to 11-14 weeks of age).
Wild animals	No wild animals were used in this study.
Reporting on sex	In the present study, we did not examined sex differences as we focused on the effects of pregnancy and delivery, as well as adolescent psychosocial stress, on postpartum behaviors.
Field-collected samples	No field-collected samples were used in this study.
Ethics oversight	All experimental procedures were performed in accordance with the National Institutes of Health Guidelines for the Care and Use of Laboratory Animals, and under animal protocol (IACUC-21547) approved by the Institutional Animal Care and Use Committees at the University of Alabama at Birmingham.

Note that full information on the approval of the study protocol must also be provided in the manuscript.
