# Supplementary material for: Adolescent stress impairs postpartum social behavior via anterior insula-prelimbic pathway in mice
Source: Nat Commun. 2023 May 23;14:2975. doi: 10.1038/s41467-023-38799-6 (PMC10205810; doi:10.1038/s41467-023-38799-6)
Supplement: Supplementary file 3 — Source data [file 41467_2023_38799_MOESM3_ESM.pdf]

Fig. 1c, e			sociability						social novelty			
		empty			mouse			familiar			novel	
	Total interaction itme	Number of visit	Interaction time per visit	Total interaction itme	Number of visit	Interaction time per visit	Total interaction itme	Number of visit	Interaction time per visit	Total interaction itme	Number of visit	Interaction time per visit
Unstressed dam_1	59.8596	7	8.551371429	116.998	13	8.999846154	109.8629	18	6.103494444	177.5454	13	13.65733846
Unstressed dam_2	70.0231	10	7.00231	113.077	14	8.076928571	48.2542	11	4.386745455	111.0669	12	9.255575
Unstressed dam_3	51.2969	5	10.25938	110.677	10	11.0677	56.0965	10	5.60965	106.5055	13	8.192730769
Unstressed dam_4	84.8278	4	21.20695	176.0249	4	44.006225	57.4588	11	5.223527273	124.9878	12	10.41565
Unstressed dam_5	55.7393	4	13.934825	155.669	5	31.1338	79.7982	16	4.9873875	156.8595	7	22.4085
Unstressed dam_6	75.8648	5	15.17296	130.836	5	26.1672	82.0971	10	8.20971	174.5844	7	24.94062857
Unstressed dam_7	118.182	10	11.8182	168.469	13	12.95915385	57.4902	10	5.74902	145.326	11	13.21145455
Unstressed dam_8	94.7767	11	8.616063636	177.081	12	14.75675	69.379	7	9.911285714	145.928	15	9.728533333
Stressed dam_1	90.5838	10	9.05838	140.0383	14	10.00273571	68.7334	14	4.909528571	82.8973	13	6.376715385
Stressed dam_2	36.4898	5	7.29796	185.7832	7	26.54045714	79.4589	10	7.94589	80.1791	5	16.03582
Stressed dam_3	49.7733	7	7.110471429	104.3479	14	7.453421429	69.2137	5	13.84274	90.5796	11	8.234509091
Stressed dam_4	63.8579	8	7.9822375	175.4611	8	21.9326375	116.507	9	12.94522222	102.5831	10	10.25831
Stressed dam_5	88.0238	9	9.780422222	86.9835	8	10.8729375	83.6173	16	5.22608125	91.5395	11	8.321772727
Stressed dam_6	52.6545	5	10.5309	110.2703	5	22.05406	75.2146	7	10.74494286	65.7721	4	16.443025
Stressed dam_7	95.7769	6	15.96281667	195.756	10	19.5756	158.735	11	14.43045455	129.923	18	7.217944444
Stressed dam_8	88.9738	6	14.82896667	171.611	11	15.601	141.592	14	10.11371429	115.381	12	9.615083333

Fig. 1g

	PrL	
	Vglut1$^+$c-Fos$^+$/mm^2	Vgat$^+$c-Fos$^+$/mm^2
Unstressed dam_1	212.2719735	58.96226415
Unstressed dam_2	168.9533516	41.87396352
Unstressed dam_3	215.3508772	33.33333333
Unstressed dam_4	154.5081967	61.40350877
Unstressed dam_5	134.3891403	50.87719298
Unstressed dam_6	185.9728507	63.15789474
Unstressed dam_7	149.009901	51.92307692
Unstressed dam_8	202.764977	62.08530806
Stressed dam_1	87.18152866	61.5720524
Stressed dam_2	133.3333333	63.17991632
Stressed dam_3	86.03603604	59.4095941
Stressed dam_4	93.7254902	55.76923077
Stressed dam_5	56.73469388	40.72580645
Stressed dam_6	92.88888889	43.60189573
Stressed dam_7	103.4632035	38.64541833
Stressed dam_8	95.67307692	44.27860697

Fig. 1i	AI	
	Vglut1^{+}c-Fos^{+}/mm^{2}	Vgat^{+}c-Fos^{+}/mm^{2}
Unstressed dam_1	351.0602491	104.4776119
Unstressed dam_2	605.2114061	131.1030741
Unstressed dam_3	439.4833948	188.3458647
Unstressed dam_4	560.6837607	196.0159363
Unstressed dam_5	518.1818182	123.0215827
Unstressed dam_6	674.3455497	133.1877729
Unstressed dam_7	553.038674	174.8603352
Unstressed dam_8	657.0707071	152.0408163
Stressed dam_1	160.4144337	114.1365162
Stressed dam_2	211.0526316	157.591623
Stressed dam_3	170.4861111	174.7330961
Stressed dam_4	229.5774648	177.4907749
Stressed dam_5	130.7692308	129.8507463
Stressed dam_6	211.0047847	84.76190476
Stressed dam_7	231.7535545	94.04255319
Stressed dam_8	227.6190476	93.89671362

Fig. 1k	EGFP/Vglut/cFOS	area (mm2)	EGFP/Vglut/cFOS / mm2	converted	EGFP/Vgat/cFOS	area (mm2)	EGFP/Vgat/cFOS / mm2	converted
Unstressed dam_1	15	0.652	23.00613497	0.888326041	2	0.621	3.220611916	0.713735789
Unstressed dam_2	18	0.601	29.95008319	1.156449742	1	0.649	1.540832049	0.341471437
Unstressed dam_3	13	0.633	20.5371248	0.792991209	4	0.602	6.644518272	1.472524667
Unstressed dam_4	12	0.644	18.63354037	0.719488918	3	0.631	4.754358162	1.053636905
Unstressed dam_5	19	0.581	32.70223752	1.262717499	2	0.633	3.159557662	0.700205252
Unstressed dam_6	22	0.655	33.58778626	1.296910813	4	0.551	7.259528131	1.608820054
Unstressed dam_7	18	0.605	29.75206612	1.148803793	3	0.659	4.552352049	1.008869328
Unstressed dam_8	12	0.631	19.01743265	0.734311986	3	0.604	4.966887417	1.100736568
			25.89830074	1			4.512330707	1
Stressed dam_1	10	0.601	16.63893511	0.642472079	2	0.581	3.442340792	0.762874225
Stressed dam_2	4	0.581	6.884681583	0.265835263	3	0.656	4.573170732	1.01348306
Stressed dam_3	4	0.601	6.655574043	0.256988831	1	0.65	1.538461538	0.340946096
Stressed dam_4	6	0.631	9.508716323	0.367155993	3	0.619	4.846526656	1.074062823
Stressed dam_5	3	0.652	4.601226994	0.177665208	3	0.624	4.807692308	1.06545655
Stressed dam_6	6	0.633	9.478672986	0.365995942	5	0.661	7.56429652	1.676361289
Stressed dam_7	8	0.644	12.42236025	0.479659279	2	0.601	3.327787022	0.737487396
Stressed dam_8	3	0.652	4.601226994	0.177665208	3	0.623	4.81540931	1.067166753
			8.848924285	0.341679725			4.36446061	0.967229774

Fig. 2e, g	social novelty (w/o opto)						social novelty (w opto)					
	novel mouse (s)	visit to novel	time per visit (s)	familiar mouse (s)	visit to familiar	time per visit (s)	novel mouse (s)	visit to novel	time per visit (s)	familiar mouse (s)	visit to familiar	time per visit (s)
Stressed dams / tdTomato1	137.396	12	11.44966667	124.753	10	12.4753	106.108	8	13.2635	192.531	6	32.0885
Stressed dams / tdTomato2	77.7004	8	9.71255	88.9834	7	12.71191429	74.1795	13	5.706115385	91.384	10	9.1384
Stressed dams / tdTomato3	110.991	11	10.09009091	133.317	14	9.522642857	91.4693	12	7.622441667	114.677	13	8.821307692
Stressed dams / tdTomato4	72.3395	5	14.4679	109.39	6	18.23166667	138.198	7	19.74257143	80.9821	6	13.49701667
Stressed dams / tdTomato5	92.585	3	30.86166667	96.346	8	12.04325	91.4649	4	22.866225	137.637	8	17.204625
Stressed dams / tdTomato6	76.9009	4	19.225225	86.5836	5	17.31672	116.432	7	16.63314286	109.31	6	18.21833333
Stressed dams / tdTomato7	83.1422	6	13.85703333	70.4989	6	11.74981667	58.7363	7	8.3909	67.6188	5	13.52376
Stressed dams / tdTomato8	141.802	6	23.63366667	138.121	8	17.265125	205.672	5	41.1344	175.261	7	25.03728571
Stressed dams / Chrimson1	71.9396	7	10.27708571	101.5477	5	20.30954	211.942	7	30.27742857	59.4773	5	11.89546
Stressed dams / Chrimson2	84.7431	11	7.703918182	131.076	14	9.362571429	129.635	12	10.80291667	63.2971	10	6.32971
Stressed dams / Chrimson3	45.3721	10	4.53721	80.6616	16	5.04135	92.2656	6	15.3776	62.4973	8	7.8121625
Stressed dams / Chrimson4	66.4181	11	6.038009091	131.796	15	8.7864	104.509	10	10.4509	71.6197	14	5.115692857
Stressed dams / Chrimson5	124.033	3	41.34433333	98.1863	9	10.90958889	155.163	8	19.395375	74.981	7	10.71157143
Stressed dams / Chrimson6	112.351	7	16.05014286	105.789	5	21.1578	132.917	5	26.5834	74.981	8	9.372625
Stressed dams / Chrimson7	104.829	8	13.103625	73.54	4	18.385	181.89	6	30.315	58.3359	6	9.72265
Stressed dams / Chrimson8	107.233	4	26.80825	127.399	7	18.19985714	137.721	9	15.30233333	61.2984	8	7.6623
Unstressed dams / mCherry1	134.709	6	22.4515	76.6334	11	6.966672727	125.23	9	13.91444444	66.6203	7	9.517185714
Unstressed dams / mCherry2	209.336	9	23.25955556	106.188	7	15.16971429	182.0505	10	18.20505	64.1778	7	9.168257143
Unstressed dams / mCherry3	169.326	9	18.814	55.615	5	11.123	157.5637	7	22.5091	90.6652	6	15.11086667
Unstressed dams / mCherry4	111.711	6	18.6185	60.7368	5	12.14736	94.1078	6	15.68463333	97.9489	4	24.487225
Unstressed dams / mCherry5	200.134	11	18.194	57.6956	7	8.242228571	150.441	6	25.0735	61.0566	11	5.5506
Unstressed dams / mCherry6	184.2897	8	23.0362125	94.2654	8	11.783175	189.2511	8	23.6563875	111.23	8	13.90375
Unstressed dams / mCherry7	254.789	9	28.30988889	68.3386	4	17.08465	153.889	8	19.236125	80.5058	5	16.10116
Unstressed dams / mCherry8	118.995	7	16.99928571	57.5371	7	8.219585714	179.7344	5	35.94688	46.414	4	11.6035
Unstressed dams / eNPHR1	150.205	7	21.45785714	115.154	12	9.596166667	138.037	8	17.254625	132.276	9	14.69733333
Unstressed dams / eNPHR2	66.0178	5	13.20356	63.8572	7	9.122457143	80.668	7	11.524	115.4001	9	12.82223333
Unstressed dams / eNPHR3	299.4401	9	33.27112222	114.5106	5	22.90212	134.676	5	26.9352	100.347	5	20.0694
Unstressed dams / eNPHR4	239.6647	10	23.96647	98.9067	7	14.12952857	187.492	9	20.83244444	148.921	12	12.41008333
Unstressed dams / eNPHR5	102.2755	7	14.61078571	100.5949	11	9.144990909	84.9059	5	16.98118	92.1881	5	18.43762
Unstressed dams / eNPHR6	166.218	10	16.6218	90.8318	7	12.97597143	99.6296	9	11.06995556	105.7114	7	15.10162857
Unstressed dams / eNPHR7	160.2076	9	17.80084444	82.5846	8	10.323075	89.7912	11	8.162836364	122.2024	12	10.18353333
Unstressed dams / eNPHR8	198.229	10	19.8229	129.485	8	16.185625	170.131	9	18.90344444	199.339	7	28.477

Fig. 2m-o, Suppl Fig. 4e	social novelty (w/o opto)						social novelty (w opto)					
	Familiar-excited	Familiar-suppressed	Familiar-unresponsible	Novel-excited	Novel-suppressed	Novel-unresponsible	Familiar-excited	Familiar-suppressed	Familiar-unresponsible	Novel-excited	Novel-suppressed	Novel-unresponsible
Stressed dams / tdTomato1	40	53	9	57	32	13	30	57	15	37	59	6
Stressed dams / tdTomato2	61	57	22	58	55	27	70	38	32	80	43	17
Stressed dams / tdTomato3	68	50	33	59	74	18	71	55	25	59	71	21
Stressed dams / tdTomato4	56	58	18	62	55	15	66	49	17	45	70	17
Stressed dams / tdTomato5	51	52	14	24	82	11	31	73	13	37	71	9
Stressed dams / tdTomato6	13	56	16	26	54	5	26	56	3	18	61	6
Stressed dams / tdTomato7	77	29	12	63	48	7	58	46	14	52	56	10
Stressed dams / tdTomato8	50	39	11	32	56	12	54	40	6	40	56	4
Stressed dams / Chrimson1	43	82	24	59	67	23	60	72	17	62	67	20
Stressed dams / Chrimson2	77	74	24	43	112	20	57	84	34	99	57	19
Stressed dams / Chrimson3	50	20	10	38	31	11	32	41	7	36	33	11
Stressed dams / Chrimson4	68	72	16	61	76	19	66	69	21	85	55	16
Stressed dams / Chrimson5	81	45	7	59	71	3	55	66	12	77	45	11
Stressed dams / Chrimson6	36	53	25	69	34	11	61	42	11	55	46	13
Stressed dams / Chrimson7	54	55	16	54	56	15	50	57	18	53	60	12
Stressed dams / Chrimson8	61	37	12	49	42	19	51	39	20	50	45	15
Unstressed dams / mCherry1	62	70	9	65	64	12	62	67	12	56	71	14
Unstressed dams / mCherry2	38	10	8	18	33	5	42	10	4	24	30	2
Unstressed dams / mCherry3	33	45	58	84	37	15	44	58	34	80	35	21
Unstressed dams / mCherry4	60	66	13	65	57	17	66	55	18	66	57	16
Unstressed dams / mCherry5	65	91	10	77	53	36	53	92	21	83	64	19
Unstressed dams / mCherry6	50	41	19	52	38	20	45	54	11	76	29	5
Unstressed dams / mCherry7	74	68	21	96	56	11	85	67	11	63	76	24
Unstressed dams / mCherry8	33	24	4	33	24	4	19	33	9	23	31	7
Unstressed dams / eNPHR1	46	65	18	73	42	14	49	67	13	51	61	17
Unstressed dams / eNPHR2	45	47	12	57	40	7	41	49	14	37	58	9
Unstressed dams / eNPHR3	76	49	13	60	58	20	62	57	19	44	80	14
Unstressed dams / eNPHR4	54	69	10	64	49	20	71	52	10	52	67	14
Unstressed dams / eNPHR5	60	38	12	47	49	14	48	46	16	52	46	12
Unstressed dams / eNPHR6	28	61	11	46	47	7	41	47	12	53	39	8
Unstressed dams / eNPHR7	54	44	28	51	62	13	41	76	9	39	72	15
Unstressed dams / eNPHR8	59	54	19	70	51	11	58	51	23	47	69	16

Fig. 3, Suppl Fig. 7, 8c	Neuronal activity during interaction with familiar mice (off->on)									Neuronal activity during interaction with novel mice (off->on)								
	unresponsible->unresponsible	unresponsible->excited	unresponsible->suppressed	excited->unresponsible	excited->excited	excited->suppressed	suppressed->unresponsible	suppressed->excited	suppressed->suppressed	unresponsible->unresponsible	unresponsible->excited	unresponsible->suppressed	excited->unresponsible	excited->excited	excited->suppressed	suppressed->unresponsible	suppressed->excited	suppressed->suppressed
Stressed dams / Chrimson1	3	14	7	8	47	18	6	29	17	3	12	8	8	37	14	9	13	45
Stressed dams / Chrimson2	2	17	5	18	23	36	14	17	43	1	15	4	2	17	24	16	67	29
Stressed dams / Chrimson3	1	4	5	2	21	27	4	7	9	1	3	7	5	21	12	5	12	14
Stressed dams / Chrimson4	1	7	8	7	30	31	13	29	30	3	8	8	5	45	11	8	32	36
Stressed dams / Chrimson5	0	5	2	9	29	43	3	21	21	0	3	0	2	37	20	9	37	25
Stressed dams / Chrimson6	0	15	10	4	20	12	7	26	20	2	1	8	6	38	25	5	16	13
Stressed dams / Chrimson7	0	8	8	5	25	24	13	17	25	2	4	9	7	29	18	3	20	33
Stressed dams / Chrimson8	0	7	5	14	31	16	6	13	18	6	7	6	4	32	13	5	11	26
Stressed dams / tdTomato1	2	4	3	9	11	20	4	15	34	0	6	7	1	25	31	5	6	21
Stressed dams / tdTomato2	4	15	3	14	34	13	14	21	22	4	13	10	0	54	4	13	13	29
Stressed dams / tdTomato3	5	15	13	12	37	19	8	19	23	1	10	7	16	32	11	4	27	43
Stressed dams / tdTomato4	2	12	4	7	28	21	8	26	24	1	7	7	9	23	29	7	15	34
Stressed dams / tdTomato5	1	6	7	8	16	27	4	9	39	2	0	9	0	4	20	7	33	42
Stressed dams / tdTomato6	0	7	9	0	9	4	3	10	43	1	0	4	1	16	9	4	2	48
Stressed dams / tdTomato7	0	6	6	7	42	28	7	10	12	1	2	4	2	37	24	7	13	28
Stressed dams / tdTomato8	1	4	6	2	21	27	3	29	7	2	6	4	0	20	12	2	14	40
Unstressed dams / eNPHR1	1	10	7	5	26	15	7	13	45	1	5	8	14	36	23	2	10	30
Unstressed dams / eNPHR2	2	7	3	9	20	16	3	14	30	1	0	6	5	16	36	3	21	16
Unstressed dams / eNPHR3	3	3	7	13	32	31	3	27	19	4	4	12	3	29	28	7	11	40
Unstressed dams / eNPHR4	4	10	6	3	34	27	7	8	34	4	10	6	3	34	27	7	8	34
Unstressed dams / eNPHR5	2	5	5	5	31	24	9	12	17	0	7	7	7	28	12	5	17	27
Unstressed dams / eNPHR6	1	7	3	6	16	6	5	18	38	1	3	3	1	16	29	6	14	27
Unstressed dams / eNPHR7	2	5	21	3	16	35	4	20	20	1	5	7	7	25	19	7	29	26
Unstressed dams / eNPHR8	4	8	7	8	35	16	11	15	28	2	4	5	7	36	27	7	7	37
Unstressed dams / mCherry1	2	4	3	5	32	25	5	26	39	2	2	8	9	40	16	3	14	47
Unstressed dams / mCherry2	0	7	1	3	27	8	1	8	1	0	2	3	1	8	9	1	14	18
Unstressed dams / mCherry3	18	19	21	8	17	8	8	8	29	4	1	10	5	79	0	12	0	25
Unstressed dams / mCherry4	1	8	4	10	31	19	7	27	32	3	7	7	4	34	27	9	25	23
Unstressed dams / mCherry5	2	2	6	11	37	17	8	14	69	2	15	19	7	47	23	10	21	22
Unstressed dams / mCherry6	3	7	9	5	18	27	3	20	18	1	12	7	2	40	10	2	24	12
Unstressed dams / mCherry7	1	8	12	4	50	20	6	27	35	3	3	5	13	51	32	8	9	39
Unstressed dams / mCherry8	0	0	4	3	12	18	6	7	11	1	1	2	2	8	8	4	14	21

Fig. 4c, e	optical stimulation	social novelty (without opto)						social novelty (with opto)					
		novel mouse (s)	visit to novel	time per visit (s)	familiar mouse (s)	visit to familiar	time per visit (s)	novel mouse (s)	visit to novel	time per visit (s)	familiar mouse (s)	visit to familiar	time per visit (s)
closed-loop_Unstressed dams / eNPHR2	exploration	197.414	12	16.45116667	72.66	5	14.532	188.129	12	15.67741667	72.8989	7	10.41412857
closed-loop_Unstressed dams / eNPHR4	exploration	115.872	9	12.87466667	68.7389	5	13.74778	173.328	6	28.888	61.777	3	20.59233333
closed-loop_Unstressed dams / eNPHR6	exploration	182.4561	8	22.8070125	104.9923	9	11.66581111	172.295	10	17.2295	124.52	11	11.32
closed-loop_Unstressed dams / eNPHR8	exploration	131.475	11	11.95227273	61.6967	10	6.16967	162.525	7	23.21785714	70.4996	8	8.81245
closed-loop_Unstressed dams / eNPHR10	exploration	89.2245	7	12.74635714	76.1009	9	8.455655556	123.394	12	10.28283333	113.231	9	12.58122222
closed-loop_Unstressed dams / eNPHR12	exploration	89.0354	9	9.892822222	67.8174	7	9.6882	65.0574	5	13.01148	55.3748	6	9.229133333
closed-loop_Unstressed dams / eNPHR14	exploration	181.81	11	16.52818182	96.9869	12	8.082241667	105.794	6	17.63233333	87.8679	9	9.7631
closed-loop_Unstressed dams / eNPHR16	exploration	154.84	9	17.20444444	62.7361	5	12.54722	126.514	9	14.05711111	49.2931	7	7.041871429
closed-loop_Unstressed dams / eNPHR1	interaction	173.96	6	28.99333333	111.612	6	18.602	130.6301	7	18.66144286	174.218	9	19.35755556
closed-loop_Unstressed dams / eNPHR3	interaction	177.008	15	11.80053333	93.1451	12	7.762091667	110.751	12	9.22925	50.0939	7	7.156271429
closed-loop_Unstressed dams / eNPHR5	interaction	228.944	11	20.81309091	44.8124	5	8.96248	95.6256	9	10.62506667	72.3393	6	12.05655
closed-loop_Unstressed dams / eNPHR7	interaction	146.359	8	18.294875	80.2615	10	8.02615	115.551	12	9.62925	112.75	14	8.053571429
closed-loop_Unstressed dams / eNPHR9	interaction	100.186	10	10.0186	86.9824	8	10.8728	119.792	8	14.974	92.3447	7	13.1921
closed-loop_Unstressed dams / eNPHR11	interaction	110.03	6	18.33833333	81.0621	7	11.5803	90.344	12	7.528666667	110.189	12	9.182416667
closed-loop_Unstressed dams / eNPHR13	interaction	209.656	9	23.29511111	62.0166	5	12.40332	111.476	11	10.13418182	96.3508	8	12.04385
closed-loop_Unstressed dams / eNPHR15	interaction	118.752	10	11.8752	84.9827	13	6.537130769	113.951	11	10.35918182	110.91	12	9.2425
closed-loop_Stressed dams / Chrimson3	exploration	97.1915	8	12.1489375	127.697	9	14.18855556	89.048	6	14.84133333	74.4291	5	14.88582
closed-loop_Stressed dams / Chrimson4	exploration	98.4619	4	24.615475	74.4305	8	9.3038125	78.0991	8	9.7623875	53.2676	8	6.65845
closed-loop_Stressed dams / Chrimson6	exploration	101.526	9	11.28066667	131.63	8	16.45375	134.442	14	9.603	122.56	10	12.256
closed-loop_Stressed dams / Chrimson7	exploration	136.978	9	15.21977778	147.325	6	24.55416667	167.885	7	23.98357143	126.765	10	12.6765
closed-loop_Stressed dams / Chrimson8	exploration	117.151	6	19.52516667	101.197	6	16.86616667	76.7618	5	15.35236	91.1129	8	11.3891125
closed-loop_Stressed dams / Chrimson10	exploration	119.632	8	14.954	142.918	9	15.87977778	106.274	6	17.71233333	99.5518	8	12.443975
closed-loop_Stressed dams / Chrimson12	exploration	106.109	7	15.15842857	87.8639	7	12.55198571	105.309	10	10.5309	126.194	10	12.6194
closed-loop_Stressed dams / Chrimson14	exploration	92.1844	9	10.24271111	86.2629	6	14.37715	82.0219	6	13.67031667	127.954	9	14.21711111
closed-loop_Stressed dams / Chrimson1	interaction	119.482	10	11.9482	106.8	9	11.86666667	175.886	12	14.65716667	79.299	8	9.912375
closed-loop_Stressed dams / Chrimson2	interaction	129.362	5	25.8724	96.5211	5	19.30422	193.462	9	21.49577778	124.859	10	12.4859
closed-loop_Stressed dams / Chrimson5	interaction	88.3818	8	11.047725	88.1147	9	9.790522222	190.278	11	17.298	124.29	9	13.81
closed-loop_Stressed dams / Chrimson9	interaction	88.5072	11	8.046109091	129.16	8	16.145	128.915	6	21.48583333	68.9788	6	11.49646667
closed-loop_Stressed dams / Chrimson11	interaction	118.432	5	23.6864	138.997	6	23.16616667	184.289	8	23.036125	69.6986	10	6.96986
closed-loop_Stressed dams / Chrimson13	interaction	147.801	11	13.43645455	134.357	12	11.19641667	140.438	10	14.0438	53.6145	7	7.659214286
closed-loop_Stressed dams / Chrimson15	interaction	138.837	7	19.83385714	129.475	10	12.9475	151.243	12	12.60358333	117.073	11	10.643
closed-loop_Stressed dams / Chrimson16	interaction	68.1782	6	11.36303333	93.1449	8	11.6431125	170.054	9	18.89488889	93.9498	7	13.4214

Fig. 4f, Suppl Fig. 13f	optical stimulation	social novelty (w/o opto)						social novelty (w opto)					
		Familiar-excited	Familiar-suppressed	Familiar-unresponsible	Novel-excited	Novel-suppressed	Novel-unresponsible	Familiar-excited	Familiar-suppressed	Familiar-unresponsible	Novel-excited	Novel-suppressed	Novel-unresponsible
closed-loop_Unstressed dams / eNPHR2	exploration	53	49	18	53	46	21	55	57	8	51	42	27
closed-loop_Unstressed dams / eNPHR4	exploration	101	47	22	112	40	18	66	85	19	82	66	22
closed-loop_Unstressed dams / eNPHR6	exploration	80	94	15	107	61	21	94	75	20	91	83	15
closed-loop_Unstressed dams / eNPHR8	exploration	57	39	6	45	41	16	57	34	11	42	49	11
closed-loop_Unstressed dams / eNPHR10	exploration	40	53	10	47	43	13	55	39	9	45	48	10
closed-loop_Unstressed dams / eNPHR12	exploration	60	40	24	60	52	12	37	55	32	66	43	15
closed-loop_Unstressed dams / eNPHR14	exploration	46	40	11	47	39	11	26	61	10	57	33	7
closed-loop_Unstressed dams / eNPHR16	exploration	19	67	30	20	75	21	49	54	13	48	54	14
closed-loop_Unstressed dams / eNPHR1	interaction	34	39	9	33	36	13	45	28	9	18	48	16
closed-loop_Unstressed dams / eNPHR3	interaction	53	48	16	57	50	10	52	49	16	57	43	17
closed-loop_Unstressed dams / eNPHR5	interaction	67	60	29	69	65	22	65	70	21	69	67	20
closed-loop_Unstressed dams / eNPHR7	interaction	82	89	26	99	83	15	87	80	30	76	103	18
closed-loop_Unstressed dams / eNPHR9	interaction	34	60	7	45	43	13	36	59	6	30	59	12
closed-loop_Unstressed dams / eNPHR11	interaction	64	65	21	70	56	24	66	69	15	74	60	16
closed-loop_Unstressed dams / eNPHR13	interaction	94	69	30	111	65	17	91	85	17	76	104	13
closed-loop_Unstressed dams / eNPHR15	interaction	65	42	8	54	49	12	32	74	9	58	41	16
closed-loop_Stressed dams / Chrimson3	exploration	21	22	10	24	24	5	26	20	7	27	21	5
closed-loop_Stressed dams / Chrimson4	exploration	75	74	17	77	65	24	74	71	21	82	67	17
closed-loop_Stressed dams / Chrimson6	exploration	67	90	14	61	94	16	90	62	19	36	105	30
closed-loop_Stressed dams / Chrimson7	exploration	75	52	16	42	88	13	71	58	14	43	83	17
closed-loop_Stressed dams / Chrimson8	exploration	24	30	10	37	20	7	23	31	10	20	39	5
closed-loop_Stressed dams / Chrimson10	exploration	52	61	9	57	53	12	39	68	15	64	46	12
closed-loop_Stressed dams / Chrimson12	exploration	56	46	13	58	44	13	53	53	9	53	48	14
closed-loop_Stressed dams / Chrimson14	exploration	85	61	17	55	94	14	90	57	16	55	92	16
closed-loop_Stressed dams / Chrimson1	interaction	52	54	19	60	52	13	40	66	19	39	68	18
closed-loop_Stressed dams / Chrimson2	interaction	45	37	6	37	40	11	33	44	11	36	40	12
closed-loop_Stressed dams / Chrimson5	interaction	82	53	15	66	68	16	63	70	17	99	35	16
closed-loop_Stressed dams / Chrimson9	interaction	28	29	2	27	28	4	18	29	12	28	24	7
closed-loop_Stressed dams / Chrimson11	interaction	70	71	12	44	95	14	89	49	15	82	50	21
closed-loop_Stressed dams / Chrimson13	interaction	37	46	20	48	39	16	31	55	17	53	40	10
closed-loop_Stressed dams / Chrimson15	interaction	69	71	13	64	73	16	64	75	14	78	54	21
closed-loop_Stressed dams / Chrimson16	interaction	69	80	18	69	82	16	76	72	19	67	89	11

Fig. 5c	GR and GFAP /GFAP (%)	GFP / mm2
group/GR/CRE-DOG 1wks 1	91.48418491	62.7480916
group/GR/CRE-DOG 1wks 2	94.67849224	84.66303736
group/GR/CRE-DOG 1wks 3	88.70967742	80.58489033
group/GR/CRE-DOG 1wks 4	91.39344262	79.28513404
group/GR/CRE-DOG 2wks 1	74.52991453	110.7535025
group/GR/CRE-DOG 2wks 2	84.2883549	87.65392093
group/GR/CRE-DOG 2wks 3	80.73089701	96.69129457
group/GR/CRE-DOG 2wks 4	75.71214393	111.4639037
group/GR/CRE-DOG 3wks 1	16.13949716	178.6956522
group/GR/CRE-DOG 3wks 2	18.85397412	167.751938
group/GR/CRE-DOG 3wks 3	35.02183406	192.7933996
group/GR/CRE-DOG 3wks 4	32.00692042	194.9409781

Fig. 5d, Suppl Fig. 16d	virus (to PrL)	Sociability						Social novelty					
		mouse (s)	visit to mouse	stay per visit	empty (s)	visit to empty	stay per visit	novel mouse (s)	visit to novel	stay per visit	familiar mouse (s)	visit to familiar	stay per visit
Unstressed dams / control1	AAVrg-mCherry	145.806	8	18.22575	40.973	5	8.1946	120.993	8	15.124125	67.9387	7	9.705528571
Unstressed dams / control2	AAVrg-mCherry	194.374	11	17.67036364	78.662	7	11.23742857	143.966	6	23.99433333	123.319	9	13.70211111
Unstressed dams / control3	AAVrg-mCherry	156.922	11	14.26563636	56.6552	7	8.0936	98.8274	4	24.70685	97.307	9	10.81188889
Unstressed dams / control4	AAVrg-mCherry	106.193	9	11.79922222	63.3796	8	7.92245	137.878	8	17.23475	27.3676	5	5.47352
Unstressed dams / control5	AAVrg-mCherry	144.761	6	24.12683333	14.404	4	3.601	142.119	12	11.84325	60.2567	7	8.6081
Unstressed dams / control6	AAVrg-mCherry	112.88	4	28.22	78.5688	5	15.71376	160.675	10	16.0675	99.5293	6	16.58821667
Unstressed dams / GR-KO1	AAVrg-GFP	124.887	9	13.87633333	64.88	6	10.81333333	122.157	4	30.53925	86.1776	8	10.7722
Unstressed dams / GR-KO2	AAVrg-GFP	133.482	9	14.83133333	52.1766	5	10.43532	114.032	6	19.00533333	47.6132	5	9.52264
Unstressed dams / GR-KO3	AAVrg-GFP	109.808	11	9.982545455	52.5343	9	5.837144444	98.8556	8	12.35695	34.3091	9	3.812122222
Unstressed dams / GR-KO4	AAVrg-GFP	158.738	7	22.67685714	94.6552	6	15.77586667	132.171	7	18.88157143	71.6925	5	14.3385
Unstressed dams / GR-KO5	AAVrg-GFP	174.224	8	21.778	65.7513	6	10.95855	212.807	10	21.2807	81.1712	8	10.1464
Unstressed dams / GR-KO6	AAVrg-GFP	121.891	6	20.31516667	96.2575	7	13.75107143	134.306	8	16.78825	70.6242	6	11.7707
Stressed dams / control1	AAVrg-mCherry	163.802	10	16.3802	37.8497	6	6.308283333	129.475	10	12.9475	117.712	6	19.61866667
Stressed dams / control2	AAVrg-mCherry	124.995	7	17.85642857	82.3428	5	16.46856	75.3003	5	15.06006	74.8202	4	18.70505
Stressed dams / control3	AAVrg-mCherry	104.193	10	10.4193	39.7724	4	9.9431	62.4969	6	10.41615	65.6177	6	10.93628333
Stressed dams / control4	AAVrg-mCherry	113.31	13	8.716153846	63.2969	10	6.32969	79.0613	10	7.90613	100.187	9	11.13188889
Stressed dams / control5	AAVrg-mCherry	132.997	6	22.16616667	66.7387	4	16.684675	69.8594	7	9.979914286	73.3003	7	10.47147143
Stressed dams / control6	AAVrg-mCherry	140.758	8	17.59475	63.6972	6	10.6162	154.602	6	25.767	78.5814	5	15.71628
Stressed dams / GR-KO1	AAVrg-GFP	245.426	9	27.26955556	43.3717	4	10.842925	151.162	7	21.59457143	70.8997	8	8.8624625
Stressed dams / GR-KO2	AAVrg-GFP	181.811	12	15.15091667	82.023	7	11.71757143	227.182	8	28.39775	50.0938	5	10.01876
Stressed dams / GR-KO3	AAVrg-GFP	111.31	10	11.131	59.1361	8	7.3920125	92.4252	11	8.402290909	59.2962	9	6.588466667
Stressed dams / GR-KO4	AAVrg-GFP	53.6176	3	17.87253333	46.8954	4	11.72385	145.568	5	29.1136	21.1269	6	3.52115
Stressed dams / GR-KO5	AAVrg-GFP	211.334	13	16.25646154	88.1827	7	12.59752857	120.114	12	10.0095	87.4645	11	7.951318182
Stressed dams / GR-KO6	AAVrg-GFP	84.3434	9	9.371488889	34.4095	6	5.734916667	110.911	8	13.863875	60.8969	7	8.699557143

Fig. 5f	AI_c-Fos/mm2	PrL_c-Fos/mm2
group/GR_mCherry/CRE-DOG 1	465.667	258.665
group/GR_mCherry/CRE-DOG 2	481.121	234.310
group/GR_mCherry/CRE-DOG 3	410.608	266.792
group/GR_mCherry/CRE-DOG 4	520.610	186.950
group/GR_mCherry/CRE-DOG 5	594.219	196.042
group/GR_mCherry/CRE-DOG 6	402.114	212.586
group/GR/CRE-DOG 1	505.200	261.512
group/GR/CRE-DOG 2	557.045	239.825
group/GR/CRE-DOG 3	560.099	213.974
group/GR/CRE-DOG 4	415.130	186.596
group/GR/CRE-DOG 5	396.529	231.330
group/GR/CRE-DOG 6	365.925	240.821
isolated/GR_mCherry/CRE-DOG 1	184.219	164.435
isolated/GR_mCherry/CRE-DOG 2	253.512	120.235
isolated/GR_mCherry/CRE-DOG 3	319.035	91.355
isolated/GR_mCherry/CRE-DOG 4	192.997	129.456
isolated/GR_mCherry/CRE-DOG 5	219.640	106.984
isolated/GR_mCherry/CRE-DOG 6	302.144	90.051
isolated/GR/CRE-DOG 1	320.496	152.697
isolated/GR/CRE-DOG 2	420.161	143.012
isolated/GR/CRE-DOG 3	330.137	240.713
isolated/GR/CRE-DOG 4	478.370	189.536
isolated/GR/CRE-DOG 5	538.340	220.508
isolated/GR/CRE-DOG 6	523.968	244.220

Suppl Fig. 1b, c	SIT_S						SIT_SN					
		empty			mouse			familiar			novel	
	total	N of visit	time per visit	total	N of visit	time per visit	total	N of visit	time per visit	total	N of visit	time per visit
unstressed mouse_1	82.991	8	10.373875	163.643	9	18.18255556	92.1819	11	8.380172727	163.879	14	11.70564286
unstressed mouse_2	55.6987	7	7.956957143	136.509	10	13.6509	90.8229	15	6.05486	114.109	14	8.150642857
unstressed mouse_3	71.228	8	8.9035	110.92	8	13.865	63.376	8	7.922	124.992	10	12.4992
unstressed mouse_4	55.6997	7	7.9571	142.122	12	11.8435	52.3332	10	5.23332	54.4137	6	9.06895
unstressed mouse_5	81.066	9	9.007333333	114.7444	5	22.94888	82.8205	17	4.871794118	134.033	17	7.884294118
unstressed mouse_6	54.8189	5	10.96378	156.835	9	17.42611111	53.8529	14	3.846635714	120.189	12	10.01575
unstressed mouse_7	87.467	7	12.49528571	149.632	10	14.9632	52.7328	13	4.056369231	129.952	15	8.663466667
unstressed mouse_8	66.4214	8	8.302675	155.238	14	11.08842857	64.1757	13	4.936592308	126.191	13	9.707
	69.4238375	7.375	9.495063274	141.205425	9.625	15.4960719	69.0369875	12.625	5.662718012	120.9698375	12.625	9.711868312
stressed mouse_1	81.6194	8	10.202425	151.476	7	21.63942857	102.344	12	8.528666667	161.958	11	14.72345455
stressed mouse_2	83.7009	6	13.95015	138.995	8	17.374375	53.1334	10	5.31334	117.23	12	9.769166667
stressed mouse_3	90.7422	7	12.96317143	167.641	13	12.89546154	95.7046	8	11.963075	114.429	7	16.347
stressed mouse_4	59.6177	5	11.92354	168.443	13	12.95715385	105.546	13	8.118923077	140.995	11	12.81772727
stressed mouse_5	70.106	8	8.76325	132.114	10	13.2114	66.8966	10	6.68966	114.188	8	14.2735
stressed mouse_6	101.385	8	12.673125	142.835	8	17.854375	68.5777	10	6.85777	163.322	10	16.3322
stressed mouse_7	90.5824	10	9.05824	134.033	10	13.4033	75.6985	14	5.407035714	126.191	18	7.010611111
stressed mouse_8	80.986	11	7.362363636	165.563	11	15.05118182	66.1838	18	3.676877778	91.5427	17	5.384864706
	82.34245	7.875	10.86203313	150.1375	10	15.54833447	79.260575	11.875	7.069418529	128.7319625	11.75	12.08231554

Suppl Fig. 1d	PrL_c-Fos/mm2
Unstressed dam_1	256.7353407
Unstressed dam_2	207.4363992
Unstressed dam_3	155.1459293
Unstressed dam_4	257.7487765
Unstressed dam_5	384.0155945
Unstressed dam_6	284.9740933
Unstressed dam_7	364.7912886
Unstressed dam_8	385.2295409
Stressed dam_1	181.372549
Stressed dam_2	163.0615641
Stressed dam_3	132.6053042
Stressed dam_4	118.4738956
Stressed dam_5	156.1822126
Stressed dam_6	201.3888889
Stressed dam_7	255.3191489
Stressed dam_8	187.0967742

Suppl Fig. 1g	ipsilateral								contralateral							
	M_1	M_2	M_3	M_4	F_1	F_2	F_3	F_4	M_1	M_2	M_3	M_4	F_1	F_2	F_3	F_4
MO	0.87161	4.723564	0.833248	4.774017	0.819896	4.808317	3.055657	1.243131	0.290537	1.359814	0.329184	1.232635	0.346178	1.256227	0.509276	0.558508
VO	0.387382	0.679907	0.329184	0.782626	0.346178	0.693091	0.400146	0.684623	0.05534	0.214707	0.061722	0.254353	0	0.216591	0.127319	0.333303
LO	0.193691	0.161031	0.20574	0.195656	0.227749	0.151614	0.127319	0.765697	0.110681	0.071569	0.10287	0.078263	0.109319	0.064977	0.127319	0.468426
AI	5.008301	8.767221	5.462401	9.782821	5.393095	8.706953	16.16952	7.837132	2.379635	2.809089	3.590166	3.345725	3.589323	2.880658	4.52892	3.19791
M1	6.889873	2.880658	6.264788	3.05224	6.185661	2.794022	3.237541	5.19773	1.120642	0.286277	1.10071	0.293485	1.084085	0.281568	0.418334	1.162057
M2	11.49696	6.137055	10.29729	6.476228	10.87729	6.17284	4.092397	9.19737	5.423354	1.234568	5.184652	1.389161	5.156236	1.234568	1.946162	3.324025
Cg	14.95573	11.86259	17.19988	13.71552	17.15405	11.95582	9.294289	14.11585	7.346431	3.846842	6.923156	4.324007	6.859798	3.876976	4.965442	6.134582
PrL	NA	NA	NA	NA	NA	NA	NA	NA	3.20974	3.381643	3.055241	3.893563	3.024506	3.422136	4.929065	2.188992
IL	0.705589	0.178923	0.668655	0.626101	0.674137	0.173273	1.109494	0.729664	0.096846	0.071569	0.10287	0.117394	0.10021	0	0.23645	0.369336
AM	0.73326	2.308105	0.699517	3.013109	0.692357	2.339181	2.346308	0.981894	0.041505	0.322061	0.10287	0.410878	0	0.324886	0.527465	0.126115
VM	0.344448	0.373799	0.102722	0.408719	0.190874	0.215936	0.326146	0.045017	0.06889	0.142399	0.041089	0.116777	0.036357	0.086374	0.054358	0.009003
RSA	2.324294	0.644122	2.40716	0.802191	2.541678	0.649773	1.109494	2.981713	0.484228	0.161031	0.462915	0.176091	0.455498	0.129955	0.163696	0.909828
V2	1.023796	0.697799	0.987553	0.76306	0.965655	0.71475	1.109494	0.954869	0.179856	0.071569	0.164592	0.097828	0.163979	0.043318	0.054565	0.171156
V1	0.138351	0.2326	0.133731	0.254353	0.136649	0.216591	0	0.279254	0.02767	0.071569	0.030861	0.078263	0.02733	0.064977	0	0.144131
S2	0.442723	0.554661	0.432054	0.704363	0.428168	0.584795	0.854856	0.882803	0.02767	0.143138	0.030861	0.195656	0.02733	0.129955	0.23645	0.144131
MD	0.428888	0.339953	0.41148	0.371747	0.409948	0.324886	0.527465	0.5495	0.041505	0	0.041148	0	0.03644	0	0.036377	0.081074
RE	0.87161	0.214707	0.833248	0.234788	0.819896	0.064977	0.181884	0.972885	0.041505	0.035785	0.041148	0.176091	0.03644	0.216591	0.254638	0.117107
DEN	0.124516	0.286277	0.113157	0.410878	0.118429	0.108295	0.181884	0.369336	0.096846	0.089461	0.082296	0.273919	0.0911	0	0	0.090082
BMA	2.711677	2.003936	2.592326	1.97613	2.550788	1.927659	1.291379	2.801549	1.079137	0.912507	1.110997	1.036979	1.020315	0.953	0.436522	0.80173
BLA	5.035971	12.90034	4.804033	10.97633	4.773618	13.01711	10.56748	6.161607	3.002214	2.057613	2.870075	3.600078	2.833197	2.014295	4.201528	2.891631
ECT	3.486442	6.638039	3.33299	4.930542	3.297805	6.714317	4.019644	3.071795	2.158273	1.932367	2.119124	1.408726	2.06796	1.949318	1.418698	1.990812
PRh	3.112894	6.387547	2.92151	4.147916	2.988066	6.476067	3.255729	2.414197	1.826231	1.735552	1.759078	1.193504	1.758222	1.754386	1.418698	1.270156
LEnt	2.255119	2.934335	2.479169	2.034827	2.441469	2.988954	1.964351	2.891631	1.355838	1.09143	1.296163	0.684797	1.302724	1.082954	1.127683	1.720566
Pir	1.646375	1.216676	1.573912	0.782626	1.566913	1.234568	1.109494	1.144041	0.677919	0.733584	0.648081	0.410878	0.637697	0.736409	1.073117	0.387352
S	1.258993	1.968152	1.20358	1.252201	1.211624	1.992636	2.055293	1.738582	0.857775	0.107354	0.750952	0.039131	0.783456	0.064977	0.34558	0.621566
CA1	0.954621	0.143138	0.915544	0.293485	0.901886	0.108295	0.745726	0.981894	0.05534	0	0.041148	0.078263	0.04555	0	0.23645	0.171156
DR	0.152186	0.644122	0.154305	0.958716	0.154869	0.649773	0.745726	0.35132	0.110681	0.465199	0.10287	0.234788	0.109319	0.4765	0.563841	0.153139
VTA	0.345877	1.145106	0.329184	0.958716	0.327958	1.147932	0.418334	0.80173	0.345877	0.143138	0.30861	0.704363	0.327958	0.108295	0.145507	0.369336

Suppl Fig. 1h	Unstressed dam_1	Unstressed dam_2	Unstressed dam_3	Unstressed dam_4	Unstressed dam_5	Unstressed dam_6	Unstressed dam_7	Unstressed dam_8	Stressed dam_1	Stressed dam_2	Stressed dam_3	Stressed dam_4	Stressed dam_5	Stressed dam_6	Stressed dam_7	Stressed dam_8
MO	1.297396909	0.608606043	0.612890515	1.156089875	0.708417871	1.704122095	0.847266842	1.06520985	0.892091381	0.966946623	0.76150077	0.612269715	1.120914624	0.972399486	0.934050473	1.199559061
VO	0.907329773	0.911749481	0.974550375	1.059211817	0.772641791	1.259809599	1.026992446	1.087714717	0.5090699	0.911438198	0.979550813	1.230835074	0.960153767	1.72828574	0.735939231	1.2389863
LO	1.044303981	0.537630503	0.670912706	1.699346341	1.012195844	0.746009455	0.968021759	1.321579411	0.891273534	0.865953263	0.799545235	1.433043582	0.696100555	0.645896551	1.058027448	1.763597767
AI	1.016487755	1.12592535	0.857287772	0.712391683	0.958147148	1.107615099	1.373920041	0.848225152	0.554894767	0.481652772	0.365133802	0.4469322	0.467210418	0.499618506	0.316565391	0.335879384
M1	1.4920172	1.118799981	0.815552713	0.894081389	1.031580621	0.847586301	1.056745057	0.743636738	1.309146661	1.022284377	1.038653043	0.920409446	0.925367948	0.515202037	0.851602985	0.760112604
M2	0.930986267	0.901743418	0.937514201	0.771940091	1.140432565	1.26345712	1.082724087	0.971202251	0.923934176	1.408345596	0.98790075	0.668171716	0.828213838	1.202793088	0.733055319	0.827367643
Cg	0.796820594	0.527215655	0.841671694	1.528457405	1.161415477	1.170356452	1.192851161	0.781211562	0.711981552	0.549002197	1.007995248	1.3801426	0.608711501	0.537091685	1.103601909	0.659326208
IL	1.015885252	0.788010445	0.95769935	1.186521502	1.184639932	0.960880263	1.225860172	0.680503083	1.128907496	0.696776443	0.685783106	0.736682115	0.997900108	0.501939197	0.633553658	0.910265333
AM	1.188146784	0.96816096	0.414790529	1.105636591	1.844210443	0.541031125	1.224908751	0.713114816	0.96816096	0.654017545	0.775335943	1.109387054	0.590215773	1.439378756	0.430153141	0.299553956
VM	0.767702965	0.771946221	0.476647262	0.759926968	1.026468516	1.048562906	1.643258005	1.505487157	1.555959063	1.281072962	1.077555429	0.972552998	1.673849718	1.890453391	0.592874057	0.723930427
RSA	0.493521206	0.903028785	0.842745621	1.285122839	1.560471963	0.971188967	1.095606996	0.848313623	1.443565163	1.036670741	1.420082926	1.06066055	0.832949988	1.346650852	1.008844112	0.680718905
V2	1.504559372	1.615390668	0.792110904	0.81914808	0.475559307	0.654979443	1.547504063	0.590748164	1.147545284	1.202564578	0.891035161	0.509122203	0.291748801	0.826907631	0.69548199	0.576114571
V1	1.52460015	0.963278144	0.811528847	1.519015535	0.739595579	0.861696085	0.499026764	1.081258896	0.591233591	0.77302214	1.437296344	0.403004121	0.999114384	0.680937998	0.849879578	1.638016752
S2	1.254995529	1.259487553	0.889634238	1.422701982	1.005509286	0.662816964	0.766403392	0.738451056	0.844265406	0.966656147	1.19356511	1.272708759	1.043610733	0.802147857	1.021271175	0.618799776
MD	0.881283016	1.029666482	1.139447099	1.123229349	0.649584195	1.477168329	1.115818012	0.583803517	1.566355851	0.800438045	1.424308874	1.431325174	1.048192678	1.627781571	0.790636763	1.814576177
RE	1.240007068	1.082171155	1.076445382	0.739751776	0.357344574	0.957653554	1.193154724	1.353471767	1.399197165	1.116602309	0.952612655	1.262690124	1.400675919	1.349722538	0.57514562	0.364167382
DEN	0.942489398	0.724228695	1.011790089	0.499597596	0.380120033	1.478162083	1.951822016	1.011790089	0.967524273	1.411975721	0.720976171	0.860021576	0.747844848	0.654364242	0.882073411	1.037957074
BMA	0.895730882	1.371036464	1.597441126	0.578531023	0.977438044	0.59723686	1.089392714	0.893192887	0.773122148	1.126519175	1.139201357	0.721736386	1.230007262	1.282156693	1.928875025	1.11538293
BLA	0.990102773	1.08199753	0.83248028	0.937277168	1.102928872	0.842211848	1.164495875	1.048505655	0.432473548	0.856640644	1.09980166	0.980082827	0.695086685	0.746110102	0.464110675	0.432655998
ECT	1.580022598	1.247295714	0.355627938	0.837763838	1.127029465	0.805776491	1.383139802	0.663344154	1.42570124	0.588589706	0.602142759	0.824724712	0.857847218	0.976160948	1.270905567	1.11757696
PRh	1.587975589	0.9791783	1.735930641	1.566571554	0.44702548	0.434808944	0.673856591	0.574652902	0.885923224	0.840657073	0.890872515	0.398574865	0.708738579	1.366583696	1.007404334	1.87923108
LEnt	0.721710097	0.932300277	0.501187567	0.608182666	1.169437657	1.376142134	1.850538711	0.84050089	1.843076861	0.855475331	1.069200144	1.603800216	1.600566748	0.888805538	1.919441748	1.533044324
Pir	1.552822144	1.612273707	1.474735716	1.12552453	0.898152475	0.398446506	0.584607321	0.353437601	1.718652831	1.030582929	0.926902554	1.649593067	1.15456283	1.127181894	0.511477316	0.792892546
S	0.702837901	1.023071018	1.165360981	1.872227656	0.702259017	0.680403175	1.020207238	0.833633015	0.621026251	1.302575956	1.155680301	1.438145989	1.690069947	1.531098053	0.887751914	1.219017022
CA1	1.382699434	1.040245149	1.282876055	0.830991783	0.802537138	1.014188912	1.219408636	0.427052895	1.402390884	1.151932519	0.926901246	0.842369666	0.867656878	0.82991717	0.831815425	0.526100995
DR	0.636574651	1.054623676	1.183040562	0.580129608	1.486370909	1.29413528	0.836917942	0.928207373	1.948014158	1.245106366	0.578501174	0.33361561	0.641301691	1.624362903	0.808695694	1.115680836
VTA	1.504681535	1.129046816	1.107519583	1.361691527	0.789917533	1.051807621	0.564824672	0.601508754	1.470957813	0.926489578	1.600008466	1.108476582	0.910476523	1.157948269	0.800095487	1.006794823

Suppl Fig. 1j	Vglut1+/EGFP+_Labeled cell (%)	Vgat+/EGFP+_Labeled cell (%)
male_1	78.26086957	14.90514905
male_2	87.97250859	22.97734628
male_3	82.4933687	25.31969309
male_4	70.08547009	20.15915119
male_5	76.73611111	16.27906977
male_6	75.09433962	9.502262443
female_1	82.9787234	16.89750693
female_2	75.42857143	25.24916944
female_3	65.71428571	17.77188329
female_4	78.57142857	16.80327869
female_5	85.97402597	19.34673367
female_6	75.07331378	20.51282051

Suppl Fig. 2b		normalized		
	Layer 1	Layer 2/3	Layer 5	Layer 6
Layer study 1	0	0.458475	0.668216	1
Layer study 2	0	0.35466	0.904739	1
Layer study 3	0	0.526506	0.885481	1
Layer study 4	0	0.466314	0.252366	1

Suppl Fig. 3a, b	Cell ID	Number of Ca transient w/o opto	Number of Ca transient w opto	modulation index	average peak amplitude w opto	average peak amplitude w opto	Relative peak amplitude
Unstressed dams / mCherry	1	4	0	0	0.003809101	0	0
	2	4	0	0	0.010995846	0	0
	3	4	0	0	0.01107608	0	0
	4	5	0	0	0.008941425	0	0
	5	7	0	0	0.007736983	0	0
	6	13	0	0	0.010466582	0	0
	7	14	0	0	0.006868269	0	0
	8	93	5	0.051020408	0.004368539	0.003419298	0.782709724
	9	16	1	0.058823529	0.009017657	0.007550981	0.837355097
	10	25	3	0.107142857	0.008502547	0.006153917	0.723773347
	11	8	1	0.111111111	0.004296416	0.01722489	4.009130285
	12	116	16	0.121212121	0.004112354	0.00352308	0.856706329
	13	7	1	0.125	0.007552167	0.005745491	0.760773857
	14	7	1	0.125	0.007728252	0.006438754	0.833144918
	15	37	7	0.159090909	0.005793681	0.005336708	0.921125497
	16	5	1	0.166666667	0.011662246	0.005207301	0.44650929
	17	5	1	0.166666667	0.01470612	0.01647176	1.12006158
	18	5	1	0.166666667	0.009213059	0.009215496	1.000264516
	19	10	2	0.166666667	0.010391421	0.005248182	0.505049507
	20	28	6	0.176470588	0.004218413	0.003569931	0.846273325
	21	18	4	0.181818182	0.004476487	0.003778206	0.844011418
	22	4	1	0.2	0.006875334	0.009615733	1.398584076
	23	4	1	0.2	0.010841258	0.0120262	1.109299359
	24	4	1	0.2	0.01445815	0.01607646	1.111930641
	25	8	2	0.2	0.005372159	0.004959241	0.923137306
	26	16	4	0.2	0.007560581	0.00712373	0.942219973
	27	7	2	0.222222222	0.006455654	0.006452901	0.999573519
	28	10	3	0.230769231	0.004228315	0.004229031	1.000169311
	29	40	13	0.245283019	0.010821086	0.008521716	0.787510194
	30	3	1	0.25	0.01963149	0.0114837	0.58496324
	31	3	1	0.25	0.011644303	0.008554955	0.734690174
	32	3	1	0.25	0.008909418	0.02295502	2.576489187
	33	3	1	0.25	0.033854197	0.03387684	1.000668849
	34	3	1	0.25	0.016370767	0.01152604	0.704062322
	35	3	1	0.25	0.01390168	0.01574312	1.132461688
	36	3	1	0.25	0.013720267	0.01255497	0.915067491
	37	12	4	0.25	0.013805959	0.012960093	0.938731754
	38	12	4	0.25	0.004869036	0.00552872	1.135485494
	39	23	8	0.258064516	0.008700559	0.007326897	0.84211789
	40	17	6	0.260869565	0.012328682	0.013039777	1.057678146
	41	14	5	0.263157895	0.008759931	0.009346616	1.066973736
	42	8	3	0.272727273	0.017091779	0.01313939	0.768754978
	43	8	3	0.272727273	0.005438307	0.003994035	0.73442618
	44	34	13	0.276595745	0.008652067	0.008440859	0.975588782
	45	5	2	0.285714286	0.004200406	0.003491599	0.83125278
	46	5	2	0.285714286	0.007887224	0.005262965	0.667277169
	47	5	2	0.285714286	0.01479392	0.012007262	0.81163487
	48	10	4	0.285714286	0.002955005	0.003966096	1.342162163
	49	12	5	0.294117647	0.018633978	0.017206484	0.923392938
	50	7	3	0.3	0.003166193	0.004145214	1.309210778
	51	9	4	0.307692308	0.010644374	0.008920578	0.838055706
	52	18	8	0.307692308	0.018689321	0.015645695	0.837146258
	53	11	5	0.3125	0.004828052	0.004017959	0.832211206
	54	15	7	0.318181818	0.004193399	0.003914295	0.933442066
	55	4	2	0.333333333	0.003278311	0.003442076	1.049953844
	56	4	2	0.333333333	0.008079609	0.008960541	1.109031452
	57	6	3	0.333333333	0.019601705	0.019195487	0.979276378
	58	8	4	0.333333333	0.013838021	0.013147314	0.950086243
	59	12	6	0.333333333	0.002980441	0.003254321	1.091892233
	60	16	8	0.333333333	0.004259263	0.003727381	0.875123573
	61	17	9	0.346153846	0.022582231	0.024826872	1.099398579
	62	15	8	0.347826087	0.013040632	0.009495695	0.728162184
	63	7	4	0.363636364	0.00341948	0.003341593	0.977222683
	64	7	4	0.363636364	0.006964343	0.008198435	1.177201449
	65	12	7	0.368421053	0.022252283	0.024341529	1.093889068
	66	5	3	0.375	0.011610562	0.008544373	0.735913788
	67	5	3	0.375	0.007456665	0.006589095	0.883651671
	68	15	9	0.375	0.018615396	0.012585079	0.676057571
	69	25	15	0.375	0.008546189	0.007889753	0.923189529
	70	16	10	0.384615385	0.004027417	0.00346069	0.859282645
	71	11	7	0.388888889	0.032225137	0.043664976	1.354997353
	72	22	14	0.388888889	0.011540611	0.011557941	1.001501587
	73	3	2	0.4	0.03268505	0.02255257	0.689996497
	74	3	2	0.4	0.010976998	0.010760152	0.980245448
	75	3	2	0.4	0.016881347	0.015697405	0.929866871
	76	3	2	0.4	0.008294816	0.007710936	0.929608987
	77	6	4	0.4	0.007576691	0.006042339	0.797490439
	78	6	4	0.4	0.005875618	0.005389056	0.917189562
	79	9	6	0.4	0.021850662	0.021376158	0.978284233
	80	9	6	0.4	0.003728951	0.003312616	0.888350496
	81	9	6	0.4	0.00245595	0.0024804	1.009955596
	82	12	8	0.4	0.006566632	0.008989841	1.36901858
	83	21	14	0.4	0.019793916	0.014654042	0.740330625
	84	16	11	0.407407407	0.009389225	0.009396686	1.000794693
	85	27	19	0.413043478	0.016562862	0.014443314	0.872030105
	86	14	10	0.416666667	0.00987724	0.008192276	0.82940941
	87	14	10	0.416666667	0.009039724	0.007006105	0.775035252
	88	47	35	0.426829268	0.004515995	0.004504186	0.99738511
	89	4	3	0.428571429	0.011885013	0.010471307	0.881051325
	90	8	6	0.428571429	0.02341909	0.016636707	0.710390825
	91	9	7	0.4375	0.029689939	0.029292879	0.986626435
	92	5	4	0.444444444	0.00917764	0.007754981	0.844986366
	93	10	8	0.444444444	0.007594096	0.003653914	0.48115197
	94	10	8	0.444444444	0.006385964	0.005588622	0.875141472
	95	10	8	0.444444444	0.003818346	0.003956402	1.036155952
	96	15	12	0.444444444	0.005050716	0.004862099	0.962655305
	97	6	5	0.454545455	0.00475145	0.004130916	0.869401058
	98	6	5	0.454545455	0.015412098	0.013132195	0.852070569
	99	18	15	0.454545455	0.007489285	0.007242658	0.967069371
	100	24	20	0.454545455	0.006054523	0.005956513	0.98381204
	101	7	6	0.461538462	0.004657341	0.003893515	0.835995383
	102	12	11	0.47826087	0.031007823	0.023181493	0.747601419
	103	17	16	0.484848485	0.007064383	0.005978245	0.846251465
	104	23	22	0.488888889	0.008962495	0.008126916	0.90676945
	105	2	2	0.5	0.004534852	0.005240037	1.155503438
	106	2	2	0.5	0.007794146	0.008886557	1.140157818
	107	2	2	0.5	0.013296455	0.01383053	1.040166721
	108	2	2	0.5	0.006428889	0.003640872	0.566329812
	109	2	2	0.5	0.012687215	0.01539682	1.213569724
	110	3	3	0.5	0.014604313	0.014733613	1.008853549
	111	3	3	0.5	0.004370806	0.003266692	0.747388849
	112	3	3	0.5	0.003887378	0.004442824	1.142884484
	113	4	4	0.5	0.009064924	0.007657277	0.844714988
	114	4	4	0.5	0.018105013	0.019808485	1.094088447
	115	5	5	0.5	0.004320928	0.003742991	0.866247013
	116	6	6	0.5	0.009126749	0.014842324	1.626244291
	117	6	6	0.5	0.028465954	0.039858158	1.400204567
	118	8	8	0.5	0.017475459	0.019017551	1.08824326
	119	8	8	0.5	0.011041197	0.015475678	1.401630444
	120	9	9	0.5	0.010511104	0.006070421	0.577524593
	121	20	20	0.5	0.009224623	0.00875346	0.948923343
	122	7	8	0.533333333	0.006824938	0.007375375	1.080650769

123	7	8	0.533333333	0.005332958	0.005503258	1.03193347
124	20	23	0.534883721	0.011303674	0.00924584	0.81794998
125	6	7	0.538461538	0.009505137	0.013153118	1.383790452
126	5	6	0.545454545	0.002778497	0.003169916	1.140874103
127	19	23	0.547619048	0.003765629	0.004062589	1.078860658
128	4	5	0.555555556	0.008645532	0.008420505	0.973971822
129	22	28	0.56	0.005345117	0.005281964	0.988184768
130	7	9	0.5625	0.010794133	0.008061691	0.746858573
131	14	18	0.5625	0.028654566	0.025296347	0.882803352
132	3	4	0.571428571	0.005544824	0.007188214	1.296382794
133	21	28	0.571428571	0.012785161	0.013606062	1.064207292
134	29	41	0.585714286	0.003106651	0.002989661	0.962342016
135	9	13	0.590909091	0.019590674	0.015466112	0.789462962
136	2	3	0.6	0.013009185	0.016181827	1.24387705
137	2	3	0.6	0.015342232	0.021648047	1.411010254
138	2	3	0.6	0.016953285	0.017848227	1.052788688
139	4	6	0.6	0.009307885	0.007761366	0.833848533
140	4	6	0.6	0.009139342	0.008085442	0.884685437
141	4	6	0.6	0.005878968	0.007062413	1.201301459
142	4	6	0.6	0.0360226	0.040818225	1.133128231
143	8	12	0.6	0.002403792	0.003590859	1.493831213
144	9	14	0.608695652	0.010068455	0.009854102	0.978710384
145	8	13	0.619047619	0.016313515	0.015449466	0.947034818
146	22	36	0.620689655	0.004706495	0.004653256	0.988688121
147	3	5	0.625	0.014117393	0.01597895	1.131862634
148	9	15	0.625	0.009060289	0.011473736	1.266376322
149	7	12	0.631578947	0.002841352	0.002865495	1.008496865
150	7	12	0.631578947	0.009087863	0.008002255	0.880543057
151	6	11	0.647058824	0.004228673	0.004926025	1.164910522
152	2	4	0.666666667	0.02506541	0.01321555	0.527242523
153	2	4	0.666666667	0.01532488	0.01409661	0.919851248
154	5	10	0.666666667	0.00867849	0.006303533	0.726339756
155	7	14	0.666666667	0.036843264	0.035172514	0.954652479
156	7	14	0.666666667	0.041125497	0.033799927	0.821872792
157	8	16	0.666666667	0.001857645	0.004312924	2.321716181
158	4	9	0.692307692	0.002262768	0.003468742	1.532964417
159	3	7	0.7	0.015876507	0.018246891	1.149301406
160	3	7	0.7	0.01168246	0.009810888	0.839796425
161	2	5	0.714285714	0.008913158	0.017397878	1.951931986
162	2	5	0.714285714	0.006604308	0.008843106	1.33899061
163	2	5	0.714285714	0.005400915	0.004948031	0.916146727
164	5	13	0.722222222	0.012339696	0.013378947	1.08422011
165	3	8	0.727272727	0.00641867	0.005770117	0.898958324
166	9	24	0.727272727	0.003905737	0.003391607	0.868365603
167	1	3	0.75	0.0117547	0.013059383	1.110992482
168	1	3	0.75	0.007722464	0.014438795	1.869713475
169	1	3	0.75	0.009627259	0.008680449	0.901653212
170	1	3	0.75	0.02832719	0.020867267	0.736651488
171	1	3	0.75	0.01461007	0.017959123	1.229229109
172	2	6	0.75	0.007494587	0.006150031	0.82059643
173	3	9	0.75	0.014003452	0.009019607	0.644098811
174	3	9	0.75	0.009278772	0.012755208	1.3746656
175	2	7	0.777777778	0.01310837	0.017281757	1.318375751
176	4	14	0.777777778	0.02137614	0.02505028	1.171880424
177	1	4	0.8	0.01582221	0.017468045	1.104020551
178	2	8	0.8	0.005192506	0.004800883	0.924579119
179	8	36	0.818181818	0.003597739	0.005107941	1.419764282
180	1	5	0.833333333	0.004473178	0.005224926	1.168056715
181	1	5	0.833333333	0.004578167	0.005158545	1.126770867
182	2	11	0.846153846	0.004074829	0.004986009	1.223611949
183	2	13	0.866666667	0.006512243	0.005361376	0.823276408
184	1	7	0.875	0.02340506	0.02048855	0.875389766
185	3	22	0.88	0.01470123	0.014590181	0.992446254
186	1	8	0.888888889	0.02298892	0.017985844	0.782370105
187	1	9	0.9	0.005325091	0.005793226	1.087911174
188	1	10	0.909090909	0.003305629	0.004043869	1.223328268
189	2	31	0.939393939	0.003613536	0.00453615	1.255321911
190	0	4	1	0	0.012647425	Inf
191	0	4	1	0	0.008660361	Inf
192	0	4	1	0	0.015135284	Inf
193	0	4	1	0	0.006795816	Inf
194	0	5	1	0	0.010256166	Inf
195	0	6	1	0	0.004176204	Inf
196	0	10	1	0	0.009016175	Inf
197	0	12	1	0	0.005544864	Inf
198	4	0	0	0.01282107	0	0
199	4	0	0	0.008598796	0	0
200	5	0	0	0.023134724	0	0
201	8	0	0	0.006462693	0	0
202	9	1	0.1	0.010716697	0.01892282	1.765732501
203	9	1	0.1	0.011380562	0.01059068	0.93059378
204	7	1	0.125	0.005437137	0.003643446	0.670103717
205	6	1	0.142857143	0.027212457	0.02251616	0.82742107
206	11	2	0.153846154	0.013696709	0.01191169	0.869675307
207	5	1	0.166666667	0.011872127	0.009031593	0.760739276
208	5	1	0.166666667	0.02935826	0.01793397	0.610866243
209	10	2	0.166666667	0.033242851	0.034868415	1.048899657
210	4	1	0.2	0.017055425	0.01419256	0.832143438
211	4	1	0.2	0.02594995	0.01822074	0.70214933
212	4	1	0.2	0.017095945	0.01724229	1.008560217
213	7	2	0.222222222	0.072338003	0.02940152	0.406446388
214	7	2	0.222222222	0.007647706	0.010808004	1.41323478
215	10	3	0.230769231	0.059760721	0.064799347	1.084313335
216	3	1	0.25	0.010715879	0.009522501	0.888634586
217	3	1	0.25	0.010037486	0.007319268	0.729193346
218	3	1	0.25	0.01301551	0.01141683	0.877171137
219	3	1	0.25	0.011797932	0.01046929	0.887383459
220	3	1	0.25	0.015019897	0.01203092	0.800998853
221	3	1	0.25	0.007836454	0.008009923	1.022136204
222	6	2	0.25	0.020528063	0.013956086	0.679853977
223	6	2	0.25	0.022047567	0.01312712	0.595399946
224	6	2	0.25	0.009831083	0.009102869	0.925927372
225	11	4	0.266666667	0.012765615	0.017086535	1.338481118
226	7	3	0.3	0.004933747	0.003612538	0.732209819
227	11	5	0.3125	0.008839458	0.009385129	1.06173124
228	17	8	0.32	0.031685183	0.023489101	0.7413276
229	4	2	0.333333333	0.012552003	0.007571566	0.603215718
230	13	7	0.35	0.003549105	0.003270581	0.921522771
231	16	9	0.36	0.03042064	0.018635449	0.612592269
232	7	4	0.363636364	0.018896911	0.010474308	0.554286757
233	5	3	0.375	0.041438306	0.02504133	0.604303902
234	5	3	0.375	0.010018295	0.010156519	1.013797145
235	8	5	0.384615385	0.026665624	0.029109851	1.091662091
236	3	2	0.4	0.011817323	0.010753029	0.909937741
237	3	2	0.4	0.006693328	0.006425002	0.95991147
238	6	4	0.4	0.010028391	0.014365929	1.432525838
239	6	4	0.4	0.064313612	0.043662055	0.67889291
240	6	4	0.4	0.031580965	0.041170613	1.303652784
241	6	4	0.4	0.031505793	0.019064963	0.605125613
242	6	4	0.4	0.013299989	0.01958379	1.472466613
243	6	4	0.4	0.016525122	0.01820112	1.101421211
244	9	6	0.4	0.006048902	0.009600372	1.587126329
245	7	5	0.416666667	0.038449199	0.036254976	0.942931904

246	4	3	0.428571429	0.012778768	0.009761062	0.763849964
247	4	3	0.428571429	0.02299591	0.010777533	0.468671748
248	4	3	0.428571429	0.012765272	0.016652407	1.304508617
249	4	3	0.428571429	0.010587182	0.01116817	1.054876579
250	4	3	0.428571429	0.01854608	0.014810463	0.798576483
251	4	3	0.428571429	0.02448061	0.016309927	0.666238573
252	4	3	0.428571429	0.009431788	0.009316152	0.987739811
253	9	7	0.4375	0.018053664	0.004494897	0.24897422
254	9	7	0.4375	0.014609576	0.020290591	1.388855641
255	10	8	0.444444444	0.013675893	0.013354929	0.976530655
256	16	13	0.448275862	0.009492695	0.007925647	0.834920617
257	6	5	0.454545455	0.011863441	0.013233499	1.115485685
258	6	5	0.454545455	0.010056008	0.014618175	1.453675793
259	6	5	0.454545455	0.039278673	0.015907346	0.404986845
260	7	6	0.461538462	0.014248676	0.016874377	1.184276794
261	7	6	0.461538462	0.017633977	0.013529279	0.767227904
262	14	12	0.461538462	0.048750361	0.040947022	0.839932691
263	22	19	0.463414634	0.008775473	0.007689361	0.876233212
264	8	7	0.466666667	0.015778847	0.024623256	1.56052312
265	11	10	0.476190476	0.020643242	0.025169057	1.219239535
266	12	11	0.47826087	0.009620739	0.008255991	0.858145233
267	35	34	0.492753623	0.004253386	0.003682928	0.865881589
268	2	2	0.5	0.00943249	0.010785767	1.143469752
269	2	2	0.5	0.021846005	0.018095135	0.828304077
270	2	2	0.5	0.026698895	0.030073475	1.126393995
271	2	2	0.5	0.021934775	0.02677104	1.220483912
272	2	2	0.5	0.013134857	0.01093573	0.832573206
273	3	3	0.5	0.008142415	0.009889471	1.214562437
274	3	3	0.5	0.01456693	0.014281723	0.980420949
275	4	4	0.5	0.010101894	0.011729426	1.161111574
276	4	4	0.5	0.012556986	0.007898213	0.628989557
277	4	4	0.5	0.01551083	0.009034547	0.58246706
278	5	5	0.5	0.015232158	0.016666746	1.094181534
279	5	5	0.5	0.016679092	0.01516723	0.909355857
280	5	5	0.5	0.005377878	0.006752166	1.255544491
281	6	6	0.5	0.017900505	0.01383717	0.773004421
282	6	6	0.5	0.03002128	0.027974467	0.93182125
283	10	10	0.5	0.012149581	0.011386283	0.937174953
284	14	14	0.5	0.00873263	0.009042485	1.035482463
285	25	26	0.509803922	0.008741741	0.008646971	0.989158893
286	7	8	0.533333333	0.02546267	0.019976396	0.784536588
287	6	7	0.538461538	0.03498333	0.039689953	1.134539018
288	9	11	0.55	0.007851971	0.007997591	1.018545705
289	4	5	0.555555556	0.020587625	0.021915595	1.064503293
290	4	5	0.555555556	0.013688055	0.011724263	0.85653249
291	8	10	0.555555556	0.024531538	0.021996302	0.896654032
292	11	14	0.56	0.012004625	0.01413789	1.177703602
293	7	9	0.5625	0.013538754	0.011356932	0.838846165
294	7	9	0.5625	0.029295456	0.025057062	0.855322493
295	3	4	0.571428571	0.020821153	0.011709343	0.562377228
296	6	8	0.571428571	0.013847119	0.022596758	1.631874291
297	6	8	0.571428571	0.016164751	0.009769682	0.604381863
298	8	11	0.578947368	0.061302079	0.036694233	0.598580562
299	7	10	0.588235294	0.02135417	0.023708472	1.110250223
300	9	13	0.590909091	0.005967074	0.005878722	0.985193459
301	2	3	0.6	0.005969234	0.008896247	1.490349906
302	4	6	0.6	0.005509737	0.007167916	1.30095438
303	6	9	0.6	0.00793767	0.007460613	0.939899618
304	3	5	0.625	0.030097303	0.016852662	0.559939268
305	3	5	0.625	0.019174033	0.02068787	1.078952437
306	3	5	0.625	0.02123122	0.018368942	0.86518542
307	7	12	0.631578947	0.011922909	0.031031704	2.602695774
308	11	19	0.633333333	0.011006517	0.007032405	0.638930992
309	11	19	0.633333333	0.044484496	0.031836753	0.715681984
310	4	7	0.636363636	0.006465359	0.011170829	1.727797284
311	6	11	0.647058824	0.010546336	0.008220015	0.779419112
312	6	11	0.647058824	0.040093542	0.024113032	0.601419351
313	7	13	0.65	0.010439046	0.018850444	1.805763069
314	7	13	0.65	0.019099363	0.017621456	0.922620104
315	2	4	0.666666667	0.006921677	0.011830781	1.709236143
316	2	4	0.666666667	0.010353875	0.011102803	1.072333136
317	2	4	0.666666667	0.026517245	0.014533525	0.548078251
318	2	4	0.666666667	0.008339305	0.012603018	1.511279208
319	4	8	0.666666667	0.01526078	0.012561544	0.823125931
320	4	8	0.666666667	0.013368632	0.012282871	0.918782911
321	5	10	0.666666667	0.00820888	0.005526215	0.673199653
322	8	17	0.68	0.012291272	0.01812658	1.474752116
323	4	9	0.692307692	0.019488977	0.010067876	0.51659335
324	4	9	0.692307692	0.03060057	0.019921421	0.651014707
325	4	9	0.692307692	0.013807858	0.015098491	1.093470954
326	3	7	0.7	0.012402804	0.013161605	1.061179812
327	4	10	0.714285714	0.01466322	0.018079406	1.232976522
328	3	8	0.727272727	0.016931853	0.017523596	1.034948502
329	1	3	0.75	0.01156376	0.012024989	1.0398857
330	1	3	0.75	0.01073753	0.010077874	0.938565418
331	2	6	0.75	0.019925495	0.014572592	0.73135406
332	2	6	0.75	0.028623265	0.02595084	0.906634516
333	7	21	0.75	0.005451217	0.005413279	0.993040323
334	3	11	0.785714286	0.004814584	0.005284926	1.097691155
335	1	4	0.8	0.01901192	0.0159776	0.84039905
336	2	8	0.8	0.009320744	0.014302102	1.534437891
337	3	12	0.8	0.01017973	0.006659691	0.654210945
338	2	9	0.818181818	0.02663337	0.021586672	0.810512213
339	4	18	0.818181818	0.016227085	0.021944804	1.352356535
340	1	5	0.833333333	0.01227385	0.012330639	1.004626829
341	2	12	0.857142857	0.015487425	0.010616576	0.685496502
342	2	12	0.857142857	0.013394068	0.014518038	1.0839155
343	1	7	0.875	0.01502702	0.011732895	0.780786515
344	0	4	1	0	0.008040635	Inf
345	0	5	1	0	0.012536805	Inf
346	0	22	1	0	0.016381572	Inf
347	4	0	0	0.022762788	0	0
348	5	0	0	0.022871234	0	0
349	7	1	0.125	0.029487331	0.0226264	0.767326133
350	7	1	0.125	0.010178124	0.007817377	0.768056744
351	7	1	0.125	0.012091146	0.01470857	1.216474435
352	11	2	0.153846154	0.0294529	0.026949335	0.914997674
353	40	9	0.183673469	0.0060197	0.006965754	1.15715957
354	17	4	0.19047619	0.016506064	0.01115787	0.675986094
355	11	3	0.214285714	0.03819701	0.032766327	0.857824386
356	3	1	0.25	0.037910519	0.04878661	1.286888475
357	3	1	0.25	0.016603957	0.02507056	1.509914805
358	14	5	0.263157895	0.018322925	0.018223516	0.994574626
359	21	9	0.3	0.023104734	0.023890482	1.034008114
360	4	2	0.333333333	0.01115492	0.011748918	1.053249866
361	12	7	0.368421053	0.03913758	0.045629017	1.165861996
362	12	7	0.368421053	0.096566496	0.134316224	1.390919523
363	22	13	0.371428571	0.050514516	0.046158948	0.913775916
364	5	3	0.375	0.066051562	0.07516628	1.137993981
365	5	3	0.375	0.02983768	0.023316973	0.781460668
366	14	9	0.391304348	0.01049474	0.008984622	0.856107088
367	3	2	0.4	0.02910451	0.023282365	0.799957292
368	3	2	0.4	0.014916953	0.011078755	0.742695559

369	3	2	0.4	0.03491658	0.035357885	1.012638838
370	7	5	0.416666667	0.040822886	0.04763401	1.166845733
371	11	8	0.421052632	0.021296013	0.020637921	0.969097902
372	19	14	0.424242424	0.084783732	0.098699064	1.164127376
373	5	4	0.444444444	0.027814856	0.034977373	1.25750687
374	12	10	0.454545455	0.027082267	0.022307829	0.823706128
375	18	15	0.454545455	0.01803606	0.019037396	1.055518556
376	24	20	0.454545455	0.017256777	0.016973778	0.983600675
377	13	11	0.458333333	0.011934798	0.017298679	1.449432043
378	15	13	0.464285714	0.125303441	0.103135785	0.823088211
379	26	24	0.48	0.028919695	0.031116031	1.075946023
380	24	23	0.489361702	0.028951974	0.029634487	1.023573994
381	2	2	0.5	0.034865535	0.058935725	1.690372025
382	2	2	0.5	0.03814299	0.03144543	0.824409151
383	2	2	0.5	0.02653891	0.0246016	0.927001147
384	3	3	0.5	0.053744397	0.06348187	1.181181182
385	6	6	0.5	0.01689987	0.016159993	0.956219979
386	7	8	0.533333333	0.028076576	0.027739018	0.98797723
387	11	13	0.541666667	0.048307837	0.043439968	0.899232317
388	5	6	0.545454545	0.06290471	0.069393217	1.103148185
389	5	6	0.545454545	0.064675152	0.097459457	1.506907269
390	5	6	0.545454545	0.024537418	0.026591015	1.083692465
391	17	21	0.552631579	0.023671748	0.024730989	1.044747051
392	4	5	0.555555556	0.013818755	0.01246199	0.901817132
393	7	9	0.5625	0.127921603	0.108453744	0.847814146
394	7	9	0.5625	0.021564793	0.020922628	0.970221598
395	13	17	0.566666667	0.012472563	0.013039921	1.045488471
396	3	4	0.571428571	0.031210303	0.02269809	0.727262717
397	5	7	0.583333333	0.028571918	0.030952417	1.083316043
398	2	3	0.6	0.024014125	0.016980953	0.707123551
399	3	5	0.625	0.063835173	0.071210036	1.115529766
400	6	10	0.625	0.012892662	0.011567602	0.897223673
401	24	40	0.625	0.062320533	0.062212198	0.99826166
402	11	19	0.633333333	0.01190828	0.012773649	1.072669508
403	4	7	0.636363636	0.018487718	0.020906597	1.130837116
404	5	9	0.642857143	0.02922025	0.027287392	0.933852114
405	10	18	0.642857143	0.035842508	0.04838898	1.350044478
406	2	4	0.666666667	0.006832254	0.00625049	0.914850349
407	3	6	0.666666667	0.0284006	0.026830607	0.944719712
408	5	10	0.666666667	0.010046776	0.013804172	1.373990201
409	11	24	0.685714286	0.016775155	0.022890342	1.364538326
410	10	23	0.696969697	0.119810717	0.111911301	0.934067533
411	10	23	0.696969697	0.017264403	0.01779488	1.03072666
412	3	7	0.7	0.027126137	0.033638864	1.240090496
413	7	17	0.708333333	0.032910654	0.031814118	0.966681427
414	2	5	0.714285714	0.051189515	0.057716264	1.127501677
415	8	20	0.714285714	0.032215678	0.031765051	0.986012183
416	5	13	0.722222222	0.02553113	0.035811225	1.402649417
417	1	3	0.75	0.007518194	0.008930503	1.187852162
418	2	6	0.75	0.07966504	0.05005253	0.628287264
419	2	6	0.75	0.02994484	0.03059982	1.021872884
420	4	12	0.75	0.07506685	0.034996203	0.466200493
421	4	13	0.764705882	0.028796625	0.03001572	1.042334649
422	7	23	0.766666667	0.007896693	0.008609342	1.090246626
423	3	10	0.769230769	0.011432007	0.012051621	1.054199936
424	5	18	0.782608696	0.011905096	0.011227485	0.943082263
425	1	4	0.8	0.0205902	0.02432027	1.181157541
426	2	8	0.8	0.072975925	0.053946531	0.739237375
427	1	5	0.833333333	0.009862138	0.010161784	1.030383432
428	2	13	0.866666667	0.01832434	0.019793855	1.080194724
429	5	38	0.88372093	0.0122077	0.01547377	1.267541797
430	0	4	1	0	0.048165503	Inf
431	0	4	1	0	0.025634625	Inf
432	0	4	1	0	0.08189848	Inf
433	0	5	1	0	0.02783127	Inf
434	0	5	1	0	0.01816804	Inf
435	0	5	1	0	0.018774992	Inf
436	0	7	1	0	0.019319029	Inf
437	0	9	1	0	0.013846857	Inf
438	0	9	1	0	0.031950853	Inf
439	0	16	1	0	0.020767275	Inf
440	0	17	1	0	0.016850817	Inf
441	0	21	1	0	0.064028035	Inf
442	0	21	1	0	0.028712092	Inf
443	5	0	0	0.008392264	0	0
444	8	1	0.111111111	0.008594773	0.004031296	0.469040441
445	8	1	0.111111111	0.018889338	0.02924049	1.547989176
446	21	4	0.16	0.037924703	0.030993478	0.817237188
447	10	2	0.166666667	0.011661402	0.01380134	1.183506055
448	9	2	0.181818182	0.014371089	0.006782932	0.471984566
449	13	3	0.1875	0.013732254	0.005518149	0.401838559
450	34	8	0.19047619	0.008473858	0.009518923	1.123328098
451	10	3	0.230769231	0.015710787	0.016358833	1.041248496
452	6	2	0.25	0.005008059	0.004518423	0.902230315
453	6	2	0.25	0.008410158	0.01035707	1.231495367
454	24	8	0.25	0.008624987	0.011527881	1.336567863
455	28	10	0.263157895	0.036554959	0.033071137	0.904696312
456	26	12	0.315789474	0.035489262	0.037195454	1.048076307
457	28	13	0.317073171	0.021471181	0.029761966	1.386135493
458	4	2	0.333333333	0.011096305	0.013172155	1.187075788
459	15	8	0.347826087	0.008376168	0.010360089	1.236853096
460	30	17	0.361702128	0.015321017	0.017044534	1.112493653
461	14	8	0.363636364	0.010565698	0.012292203	1.163406573
462	12	7	0.368421053	0.012562838	0.011664319	0.928477984
463	8	5	0.384615385	0.019949631	0.017839236	0.894213822
464	32	20	0.384615385	0.005178289	0.006465622	1.248601917
465	9	6	0.4	0.0314985	0.080180538	2.545535131
466	10	7	0.411764706	0.00636069	0.006094783	0.958195243
467	22	16	0.421052632	0.02569339	0.030646428	1.1927748
468	4	3	0.428571429	0.00804075	0.009552637	1.188028181
469	29	23	0.442307692	0.019670531	0.022218637	1.129539235
470	14	12	0.461538462	0.006476958	0.005379333	0.830533809
471	17	15	0.46875	0.015055774	0.0212713	1.412833339
472	9	8	0.470588235	0.013848959	0.017019008	1.228901598
473	19	18	0.486486486	0.004730538	0.005237997	1.107273011
474	38	36	0.486486486	0.014165306	0.014974681	1.05713786
475	2	2	0.5	0.003519353	0.004291534	1.219409789
476	3	3	0.5	0.007206906	0.01560185	2.164847262
477	21	21	0.5	0.020891404	0.026285617	1.258202508
478	8	9	0.529411765	0.011198762	0.012525714	1.118490962
479	20	23	0.534883721	0.005415767	0.005606275	1.035176611
480	4	5	0.555555556	0.009026036	0.011417483	1.264949933
481	18	23	0.56097561	0.016027172	0.014529278	0.90654036
482	7	9	0.5625	0.004599594	0.006720861	1.461185614
483	14	18	0.5625	0.013371165	0.022587889	1.689298493
484	8	11	0.578947368	0.004623832	0.004517235	0.976946152
485	10	14	0.583333333	0.038130573	0.036999812	0.970345034
486	10	14	0.583333333	0.007869734	0.009386598	1.19274648
487	24	34	0.586206897	0.019414058	0.021092723	1.086466454
488	3	5	0.625	0.01092911	0.014772864	1.35169872
489	24	40	0.625	0.009842845	0.010295861	1.046024868
490	10	17	0.62962963	0.016464591	0.022041248	1.33870609
491	9	16	0.64	0.00813203	0.008264019	1.016230655

	492	14	25	0.641025641	0.009592908	0.010506674	1.095254335
	493	37	68	0.647619048	0.014380838	0.014245288	0.9905743
	494	2	4	0.666666667	0.01116846	0.01304577	1.168090319
	495	4	8	0.666666667	0.00521048	0.006134596	1.177357189
	496	6	12	0.666666667	0.005994901	0.007793964	1.300098981
	497	4	9	0.692307692	0.028732673	0.037036688	1.289009499
	498	3	7	0.7	0.007317767	0.009766065	1.334569023
	499	13	31	0.704545455	0.009045431	0.011623878	1.285055151
	500	6	16	0.727272727	0.022067108	0.018101408	0.820289058
	501	8	22	0.733333333	0.004925603	0.005284309	1.072824789
	502	1	3	0.75	0.01472886	0.015736297	1.068398822
	503	1	3	0.75	0.006595446	0.007517922	1.139865598
	504	15	52	0.776119403	0.020390143	0.020929235	1.026438866
	505	1	4	0.8	0.02755571	0.005657884	0.205325276
	506	5	20	0.8	0.01225657	0.015847008	1.292939827
	507	1	5	0.833333333	0.008644073	0.010878424	1.258483564
	508	1	10	0.909090909	0.008243762	0.010389629	1.260301959
	509	0	4	1	0	0.032657339	Inf
	510	0	4	1	0	0.0250868	Inf
	511	0	5	1	0	0.008124598	Inf
	512	0	5	1	0	0.00645108	Inf
Unstressed dams / eNPHR	1	5	0	0	0.009062365	0	0
	2	8	0	0	0.009979135	0	0
	3	22	1	0.043478261	0.003819628	0.003085869	0.807897737
	4	16	1	0.058823529	0.004266655	0.003340809	0.783004262
	5	24	2	0.076923077	0.005952625	0.012162816	2.043269158
	6	22	2	0.083333333	0.002032615	0.003301994	1.624505015
	7	7	1	0.125	0.010684266	0.01213617	1.135891787
	8	7	1	0.125	0.006204673	0.007389075	1.190888733
	9	14	2	0.125	0.001504085	0.001244001	0.827081542
	10	28	4	0.125	0.003523946	0.002663954	0.755957597
	11	19	3	0.136363636	0.015495992	0.012836697	0.828388178
	12	45	11	0.196428571	0.002704125	0.002453228	0.907216844
	13	16	4	0.2	0.005226037	0.006555498	1.254391832
	14	27	7	0.205882353	0.003132431	0.00273778	0.87401119
	15	30	8	0.210526316	0.007915295	0.009413754	1.189311724
	16	11	3	0.214285714	0.004684033	0.002801867	0.598173981
	17	14	4	0.222222222	0.003917409	0.003735969	0.953683788
	18	24	7	0.225806452	0.002593182	0.002715615	1.047213479
	19	13	4	0.235294118	0.003575806	0.004450245	1.244543092
	20	38	12	0.24	0.001350957	0.001570029	1.16216031
	21	43	14	0.245614035	0.001713804	0.001412888	0.82441679
	22	3	1	0.25	0.00695411	0.008055405	1.15836606
	23	3	1	0.25	0.005793439	0.005738262	0.990476007
	24	6	2	0.25	0.004908327	0.005527059	1.126057552
	25	9	3	0.25	0.004828912	0.004376133	0.906235898
	26	21	7	0.25	0.001855656	0.002135839	1.150988641
	27	68	23	0.252747253	0.002804192	0.002532194	0.903003273
	28	29	10	0.256410256	0.003794976	0.004013675	1.057628447
	29	31	11	0.261904762	0.003976764	0.003351248	0.842707225
	30	28	10	0.263157895	0.005639168	0.004262834	0.755933108
	31	46	17	0.26984127	0.001584919	0.00146044	0.921459889
	32	38	15	0.283018868	0.001859171	0.002037817	1.096088821
	33	5	2	0.285714286	0.006732085	0.005109372	0.758958377
	34	10	4	0.285714286	0.006076382	0.002738435	0.450668645
	35	42	18	0.3	0.00173678	0.00159873	0.920513886
	36	23	10	0.303030303	0.001895801	0.001673998	0.883003178
	37	16	7	0.304347826	0.003434311	0.003556111	1.035465678
	38	9	4	0.307692308	0.004634993	0.004112089	0.887183572
	39	27	12	0.307692308	0.004280684	0.003871898	0.904504474
	40	38	17	0.309090909	0.002946603	0.002746135	0.931966285
	41	60	27	0.310344828	0.0017705	0.001815623	1.025486278
	42	11	5	0.3125	0.004188457	0.004265903	1.018490227
	43	33	15	0.3125	0.003044316	0.003193509	1.049007224
	44	70	32	0.31372549	0.001248339	0.001185544	0.949697411
	45	21	10	0.322580645	0.005281803	0.004367285	0.826854906
	46	27	13	0.325	0.003727932	0.003180626	0.853187666
	47	43	21	0.328125	0.001313432	0.001233555	0.939184951
	48	4	2	0.333333333	0.006952463	0.006404683	0.921210689
	49	8	4	0.333333333	0.003540718	0.003995768	1.12851902
	50	16	8	0.333333333	0.002338487	0.002844713	1.216476083
	51	26	13	0.333333333	0.004033607	0.004544048	1.12654718
	52	28	14	0.333333333	0.003750606	0.003801562	1.013585889
	53	43	22	0.338461538	0.008538643	0.006592769	0.772109618
	54	44	23	0.343283582	0.001622764	0.001491876	0.919342653
	55	17	9	0.346153846	0.001402311	0.00160549	1.144888594
	56	13	7	0.35	0.016214118	0.016958681	1.045920696
	57	11	6	0.352941176	0.018091969	0.016882003	0.933121389
	58	27	15	0.357142857	0.003364649	0.002861502	0.850460733
	59	34	19	0.358490566	0.006881138	0.005336749	0.775561972
	60	7	4	0.363636364	0.008638589	0.008831421	1.022322118
	61	49	28	0.363636364	0.003284188	0.003040957	0.925938491
	62	39	23	0.370967742	0.002713128	0.002415817	0.890417539
	63	15	9	0.375	0.005839151	0.006299357	1.078813949
	64	23	14	0.378378378	0.012286477	0.01299429	1.057609085
	65	46	28	0.378378378	0.004396929	0.00501841	1.141344421
	66	26	16	0.380952381	0.002120192	0.002233525	1.0534538
	67	26	16	0.380952381	0.004264419	0.004039931	0.947357864
	68	17	11	0.392857143	0.00266347	0.002828877	1.062102059
	69	41	27	0.397058824	0.002304491	0.00207799	0.901713264
	70	24	16	0.4	0.018106395	0.013700333	0.756657135
	71	116	78	0.402061856	0.001415064	0.001430933	1.011213878
	72	46	31	0.402597403	0.004570454	0.005128795	1.122163204
	73	38	26	0.40625	0.001974248	0.001874978	0.94971719
	74	13	9	0.409090909	0.004581282	0.002453414	0.535529984
	75	28	20	0.416666667	0.001437789	0.001734518	1.206378335
	76	22	16	0.421052632	0.001401104	0.001419291	1.012979992
	77	34	25	0.423728814	0.008374509	0.008780234	1.048447674
	78	61	45	0.424528302	0.001141136	0.001488122	1.304069863
	79	89	66	0.425806452	0.001829198	0.002090762	1.142993892
	80	16	12	0.428571429	0.006949486	0.005197541	0.74790285
	81	24	18	0.428571429	0.002279947	0.002421272	1.061986162
	82	28	21	0.428571429	0.006573951	0.004833758	0.735289592
	83	32	24	0.428571429	0.005013283	0.004390867	0.875846631
	84	30	23	0.433962264	0.003028132	0.00401644	1.326375193
	85	18	14	0.4375	0.007668496	0.007575244	0.987839617
	86	41	32	0.438356164	0.001590561	0.001502526	0.944651839
	87	41	32	0.438356164	0.003541485	0.00281867	0.795900722
	88	23	18	0.43902439	0.005205269	0.003979873	0.764585483
	89	42	33	0.44	0.00362663	0.004495644	1.239620362
	90	38	30	0.441176471	0.003727875	0.003204736	0.859668487
	91	5	4	0.444444444	0.017875005	0.017113468	0.957396505
	92	5	4	0.444444444	0.010564391	0.008496522	0.804260479
	93	10	8	0.444444444	0.006578628	0.00644121	0.979111371
	94	32	26	0.448275862	0.003008603	0.003035026	1.008782496
	95	48	39	0.448275862	0.002497177	0.002912861	1.16646147
	96	54	44	0.448979592	0.003221684	0.003123504	0.969525253
	97	18	15	0.454545455	0.003870809	0.003367335	0.869930562
	98	18	15	0.454545455	0.003572384	0.00380634	1.065489998
	99	18	15	0.454545455	0.002339393	0.002530371	1.081635667
	100	34	29	0.46031746	0.00293439	0.002723195	0.928027505
	101	50	44	0.468085106	0.007555937	0.006113612	0.80911374

102	9	8	0.470588235	0.002610084	0.002622506	1.004758871
103	9	8	0.470588235	0.001954173	0.001908262	0.976506588
104	55	49	0.471153846	0.005510287	0.004639667	0.842001026
105	30	27	0.473684211	0.00155585	0.001846626	1.186892243
106	12	11	0.47826087	0.005971596	0.004846314	0.81156093
107	14	13	0.481481481	0.005876112	0.006101447	1.038347673
108	15	14	0.482758621	0.004945352	0.00442822	0.895430777
109	31	29	0.483333333	0.005192137	0.00630525	1.214384349
110	24	23	0.489361702	0.005833524	0.004972393	0.852382463
111	24	23	0.489361702	0.004307305	0.004227648	0.981506378
112	26	25	0.490196078	0.004922082	0.004696638	0.954197461
113	2	2	0.5	0.012266635	0.010513148	0.85705228
114	2	2	0.5	0.002532177	0.002566036	1.013371501
115	6	6	0.5	0.004808412	0.00515851	1.072809455
116	7	7	0.5	0.008189228	0.008293423	1.012723474
117	9	9	0.5	0.004818401	0.003424364	0.710684635
118	15	15	0.5	0.001345437	0.001404708	1.04405395
119	20	21	0.512195122	0.001730246	0.001650895	0.954138809
120	18	19	0.513513514	0.005384717	0.004038837	0.75005564
121	19	21	0.525	0.002266	0.003088354	1.362909678
122	9	10	0.526315789	0.003670036	0.004303195	1.172521193
123	34	38	0.527777778	0.007994861	0.007697347	0.962786849
124	7	8	0.533333333	0.004517947	0.006334595	1.402096124
125	29	34	0.53968254	0.002690082	0.003028784	1.125907696
126	20	24	0.545454545	0.007014959	0.006705043	0.955820709
127	25	30	0.545454545	0.002433	0.002533395	1.041263824
128	23	28	0.549019608	0.002516338	0.003392706	1.348271231
129	30	37	0.552238806	0.001348959	0.00156856	1.162793276
130	16	20	0.555555556	0.002299255	0.00251755	1.094941804
131	19	24	0.558139535	0.007311232	0.008143143	1.113785241
132	38	48	0.558139535	0.00126072	0.001795135	1.423897376
133	14	18	0.5625	0.002357317	0.002230667	0.946273855
134	12	16	0.571428571	0.001617493	0.002567615	1.587403717
135	21	28	0.571428571	0.008441604	0.009359091	1.1086864
136	8	11	0.578947368	0.00275473	0.00430106	1.561336226
137	29	40	0.579710145	0.002479129	0.003212234	1.295710344
138	20	28	0.583333333	0.004821466	0.004702409	0.975307011
139	31	45	0.592105263	0.004535746	0.005283557	1.164870649
140	15	22	0.594594595	0.002328782	0.002391877	1.027093475
141	8	12	0.6	0.004454995	0.005167186	1.159863269
142	9	14	0.608695652	0.00319689	0.002596643	0.812240435
143	31	52	0.626506024	0.002944666	0.002035288	0.691178084
144	15	26	0.634146341	0.003194473	0.006453322	2.020152186
145	6	11	0.647058824	0.012751482	0.013511755	1.059622388
146	25	48	0.657534247	0.004796778	0.005584315	1.164180535
147	5	10	0.666666667	0.009076206	0.010905242	1.201519972
148	6	12	0.666666667	0.0036955	0.004922967	1.332151686
149	14	29	0.674418605	0.00123947	0.001698451	1.370303538
150	14	31	0.688888889	0.00426956	0.004157552	0.973765827
151	2	5	0.714285714	0.003596439	0.003915675	1.088764581
152	10	32	0.761904762	0.006162517	0.007027802	1.140410958
153	4	13	0.764705882	0.002581885	0.003136867	1.214952282
154	7	23	0.766666667	0.002011534	0.003117944	1.550033312
155	4	14	0.777777778	0.005809121	0.004402346	0.757833508
156	1	4	0.8	0.002918414	0.002653121	0.909096687
157	8	33	0.804878049	0.003821249	0.003816091	0.998650058
158	4	21	0.84	0.00334601	0.006548258	1.957034886
159	1	8	0.888888889	0.007133045	0.007767746	1.088980425
160	4	51	0.927272727	0.004792221	0.010586341	2.209067807
161	1	17	0.944444444	0.003940308	0.004941212	1.254016646
162	0	4	1	0	0.004895045	Inf
163	4	0	0	0.003560424	0	0
164	4	0	0	0.005525281	0	0
165	4	0	0	0.002741461	0	0
166	5	0	0	0.005640864	0	0
167	6	0	0	0.003334464	0	0
168	8	0	0	0.003928905	0	0
169	10	0	0	0.002773678	0	0
170	14	1	0.066666667	0.003171001	0.002711102	0.854967191
171	12	1	0.076923077	0.008709144	0.004772039	0.547934311
172	9	1	0.1	0.00282147	0.001792633	0.635354216
173	7	1	0.125	0.005588619	0.005900667	1.055836281
174	6	1	0.142857143	0.004326376	0.003376	0.780329742
175	6	1	0.142857143	0.004985392	0.003564142	0.714917126
176	6	1	0.142857143	0.002452627	0.002358204	0.961501411
177	12	2	0.142857143	0.005494615	0.003853628	0.701346318
178	5	1	0.166666667	0.003531286	0.003596047	1.018339211
179	5	1	0.166666667	0.004765264	0.005057969	1.061424808
180	5	1	0.166666667	0.004712652	0.00377715	0.80149139
181	5	1	0.166666667	0.002333798	0.002899743	1.242499672
182	10	2	0.166666667	0.003075841	0.003407514	1.107831412
183	15	3	0.166666667	0.002185185	0.001902748	0.870749059
184	13	3	0.1875	0.003700567	0.004414557	1.192940852
185	17	4	0.19047619	0.003513048	0.003925041	1.117274863
186	4	1	0.2	0.001475145	0.001518216	1.029197982
187	4	1	0.2	0.003902572	0.003768262	0.965584235
188	4	1	0.2	0.005315809	0.003778554	0.710814512
189	4	1	0.2	0.01086547	0.008954983	0.824168988
190	39	12	0.235294118	0.006049393	0.005588441	0.9238019
191	16	5	0.238095238	0.01742777	0.014716357	0.844419976
192	3	1	0.25	0.00658614	0.007948868	1.206908508
193	3	1	0.25	0.002959595	0.003403905	1.150125141
194	3	1	0.25	0.004558961	0.004749104	1.041707529
195	3	1	0.25	0.010358177	0.006724736	0.649220011
196	3	1	0.25	0.005483281	0.004244408	0.774063605
197	12	4	0.25	0.00424332	0.004211782	0.99256773
198	56	19	0.253333333	0.002802575	0.002525052	0.900975814
199	20	7	0.259259259	0.003329008	0.00311417	0.935464917
200	11	4	0.266666667	0.002947121	0.00347685	1.179744559
201	16	6	0.272727273	0.005768889	0.00447488	0.775691849
202	16	6	0.272727273	0.004932792	0.005833643	1.182624878
203	15	6	0.285714286	0.00674781	0.006805984	1.008621167
204	12	5	0.294117647	0.002510434	0.002577493	1.026711955
205	7	3	0.3	0.002276804	0.002343019	1.02908248
206	53	24	0.311688312	0.001776433	0.002025321	1.140105429
207	24	11	0.314285714	0.003097842	0.003476557	1.122251189
208	26	12	0.315789474	0.006634555	0.005823185	0.877705446
209	15	7	0.318181818	0.002997024	0.002549914	0.850815332
210	19	9	0.321428571	0.006022717	0.006223728	1.03337554
211	25	12	0.324324324	0.010620621	0.007428694	0.699459421
212	4	2	0.333333333	0.008484252	0.008424846	0.992998027
213	4	2	0.333333333	0.009955998	0.006104118	0.613109636
214	4	2	0.333333333	0.002134797	0.002254534	1.056087879
215	4	2	0.333333333	0.003261984	0.00244345	0.749068569
216	6	3	0.333333333	0.005358978	0.006229866	1.162510028
217	8	4	0.333333333	0.005353642	0.004109931	0.767688652
218	8	4	0.333333333	0.002493083	0.003266799	1.310345225
219	10	5	0.333333333	0.005641136	0.004742506	0.840700621
220	10	5	0.333333333	0.002490178	0.00196729	0.790019925
221	14	7	0.333333333	0.004262889	0.003500728	0.821210148
222	18	9	0.333333333	0.004310226	0.003833229	0.889333654
223	24	12	0.333333333	0.002886474	0.002937992	1.017848189
224	46	24	0.342857143	0.003735432	0.004702079	1.258777822

225	19	10	0.344827586	0.002924184	0.002645303	0.90462934
226	55	29	0.345238095	0.002832281	0.003569457	1.26027641
227	13	7	0.35	0.002690607	0.002668287	0.99170423
228	13	7	0.35	0.002727539	0.003826251	1.40282166
229	22	12	0.352941176	0.004391163	0.003830334	0.872282456
230	9	5	0.357142857	0.005780717	0.008531307	1.475821633
231	9	5	0.357142857	0.005978887	0.005885969	0.984458974
232	16	9	0.36	0.007215032	0.006992652	0.969178141
233	14	8	0.363636364	0.002675121	0.003023115	1.130085237
234	12	7	0.368421053	0.002446681	0.002388129	0.976068522
235	22	13	0.371428571	0.007274095	0.007317981	1.006033237
236	5	3	0.375	0.003358929	0.004104399	1.221936986
237	10	6	0.375	0.00447932	0.003870288	0.864034662
238	20	12	0.375	0.002776074	0.00409653	1.475656124
239	28	17	0.377777778	0.004696209	0.00456369	0.9717817
240	44	27	0.38028169	0.006749625	0.005469154	0.810290075
241	13	8	0.380952381	0.005611731	0.004720086	0.841110421
242	29	18	0.382978723	0.020786518	0.018720781	0.900621295
243	37	23	0.383333333	0.002376334	0.002225562	0.936552699
244	11	7	0.388888889	0.003933462	0.004207099	1.069566575
245	11	7	0.388888889	0.003335778	0.003505246	1.050803155
246	14	9	0.391304348	0.002896759	0.002231101	0.77020604
247	17	11	0.392857143	0.00249161	0.00237659	0.95383699
248	17	11	0.392857143	0.006849489	0.006756315	0.986396986
249	17	11	0.392857143	0.009064693	0.007449253	0.821787647
250	17	11	0.392857143	0.00355692	0.002791403	0.784780884
251	34	22	0.392857143	0.001648539	0.001606631	0.974578851
252	3	2	0.4	0.008442904	0.0084827	1.004713525
253	3	2	0.4	0.003919957	0.003681528	0.939175561
254	3	2	0.4	0.001972987	0.001859078	0.942265299
255	3	2	0.4	0.003787275	0.004470773	1.180472211
256	6	4	0.4	0.007949561	0.010472102	1.317318391
257	6	4	0.4	0.002583047	0.002452232	0.949356287
258	6	4	0.4	0.009306399	0.011860325	1.274426971
259	6	4	0.4	0.002629414	0.002507219	0.953527513
260	9	6	0.4	0.011480996	0.0147963	1.288764486
261	12	8	0.4	0.012885233	0.009597464	0.744842066
262	15	10	0.4	0.003574427	0.00367848	1.029110278
263	33	22	0.4	0.003284122	0.003115424	0.94863226
264	31	21	0.403846154	0.002220394	0.002315217	1.04270563
265	28	19	0.404255319	0.002301193	0.002199255	0.955702079
266	13	9	0.409090909	0.01130025	0.009439357	0.835322862
267	13	9	0.409090909	0.002927373	0.003065487	1.047180409
268	13	9	0.409090909	0.003711237	0.005150019	1.387682623
269	33	23	0.410714286	0.002029091	0.001994292	0.982850131
270	10	7	0.411764706	0.011961653	0.010559197	0.882754031
271	34	24	0.413793103	0.004002004	0.00395016	0.98704553
272	7	5	0.416666667	0.002920643	0.00290729	0.995428199
273	7	5	0.416666667	0.00270396	0.002337049	0.864306072
274	7	5	0.416666667	0.002259629	0.001698958	0.751874701
275	22	16	0.421052632	0.005115285	0.005038548	0.984998366
276	15	11	0.423076923	0.003170548	0.003254533	1.026489061
277	63	47	0.427272727	0.002840948	0.002905951	1.022880876
278	4	3	0.428571429	0.002982634	0.002504857	0.839813694
279	4	3	0.428571429	0.003657717	0.004350862	1.189502076
280	4	3	0.428571429	0.008571162	0.009138125	1.066147764
281	8	6	0.428571429	0.00385346	0.004105421	1.065385766
282	8	6	0.428571429	0.004898504	0.005792595	1.182523205
283	8	6	0.428571429	0.006421091	0.007192174	1.120085979
284	20	15	0.428571429	0.002167313	0.001862923	0.859554122
285	21	16	0.432432432	0.013553145	0.009693607	0.715229376
286	13	10	0.434782609	0.005238602	0.004765687	0.909725022
287	9	7	0.4375	0.005072567	0.004057891	0.79996796
288	9	7	0.4375	0.001920473	0.001797246	0.935835247
289	18	14	0.4375	0.004308586	0.003755211	0.871564525
290	28	22	0.44	0.003362019	0.003617527	1.07599853
291	24	19	0.441860465	0.007045017	0.006002789	0.852061569
292	5	4	0.444444444	0.01702886	0.008473732	0.497610046
293	5	4	0.444444444	0.002530717	0.002720965	1.075175519
294	15	12	0.444444444	0.011676148	0.011486975	0.983798341
295	16	13	0.448275862	0.005238612	0.006662853	1.271873806
296	11	9	0.45	0.010245047	0.01023243	0.998768535
297	12	10	0.454545455	0.003200744	0.003697626	1.155239534
298	18	15	0.454545455	0.01303016	0.013569525	1.041393621
299	54	46	0.46	0.001677212	0.001708421	1.018608029
300	15	13	0.464285714	0.006362025	0.006780433	1.065766521
301	23	20	0.465116279	0.003888511	0.005080897	1.306643166
302	8	7	0.466666667	0.008312659	0.009659163	1.161982379
303	17	15	0.46875	0.004643682	0.008586567	1.849085975
304	36	32	0.470588235	0.004481296	0.004407289	0.983485422
305	20	18	0.473684211	0.002416556	0.002413612	0.998781691
306	20	18	0.473684211	0.002808434	0.003499841	1.246189452
307	21	19	0.475	0.006206678	0.006250195	1.007011415
308	35	32	0.47761194	0.007670301	0.007729836	1.007761681
309	13	12	0.48	0.005905952	0.006439251	1.090298666
310	13	12	0.48	0.013779703	0.024944508	1.810235547
311	15	14	0.482758621	0.003089539	0.003264462	1.056617626
312	30	28	0.482758621	0.002420456	0.002680245	1.107330479
313	21	20	0.487804878	0.00780309	0.007127244	0.913387477
314	24	23	0.489361702	0.002085025	0.001891858	0.907355056
315	2	2	0.5	0.017058179	0.007597991	0.445416301
316	2	2	0.5	0.00242719	0.002436296	1.003751664
317	2	2	0.5	0.002824301	0.002344738	0.830201142
318	3	3	0.5	0.003577075	0.00472831	1.321837192
319	3	3	0.5	0.002704236	0.003120588	1.153963159
320	3	3	0.5	0.005936715	0.00860693	1.449779912
321	4	4	0.5	0.006418072	0.005900347	0.919333323
322	4	4	0.5	0.00736668	0.006225817	0.845131971
323	4	4	0.5	0.003904948	0.003579389	0.91662923
324	4	4	0.5	0.002324882	0.00231774	0.996928124
325	5	5	0.5	0.007438057	0.008511934	1.144375976
326	5	5	0.5	0.004602865	0.004556118	0.989843978
327	5	5	0.5	0.004266935	0.002857495	0.66968329
328	5	5	0.5	0.009581541	0.008487433	0.885810868
329	5	5	0.5	0.001834459	0.001655964	0.902698637
330	8	8	0.5	0.003605919	0.004541107	1.259347946
331	9	9	0.5	0.003233043	0.002899085	0.896704646
332	11	11	0.5	0.00501654	0.005846618	1.165468192
333	12	12	0.5	0.00345961	0.00322999	0.933628444
334	14	14	0.5	0.009139027	0.006824482	0.746740533
335	14	14	0.5	0.004848968	0.005658671	1.166984626
336	15	15	0.5	0.005592229	0.007270297	1.300071293
337	18	18	0.5	0.003229826	0.003611088	1.118044055
338	24	24	0.5	0.005106278	0.004420254	0.865650875
339	35	37	0.513888889	0.002508588	0.002793847	1.113713169
340	13	14	0.518518519	0.002190875	0.002401802	1.096275271
341	9	10	0.526315789	0.00567306	0.007224349	1.273448425
342	7	8	0.533333333	0.004859121	0.006625862	1.363592716
343	11	13	0.541666667	0.00344507	0.004411086	1.280405411
344	26	31	0.543859649	0.001478786	0.001709401	1.155948372
345	5	6	0.545454545	0.002936341	0.002360209	0.803792482
346	15	18	0.545454545	0.00531794	0.004052691	0.762079146
347	9	11	0.55	0.002088265	0.002002592	0.958974084

348	18	22	0.55	0.003347255	0.002917214	0.871524445
349	4	5	0.555555556	0.004896631	0.004158238	0.849203833
350	4	5	0.555555556	0.002767184	0.002798341	1.011259316
351	4	5	0.555555556	0.002095103	0.002354488	1.123805464
352	8	10	0.555555556	0.001727642	0.00234456	1.357087081
353	8	10	0.555555556	0.009208401	0.010102396	1.097084688
354	16	20	0.555555556	0.00445873	0.005966781	1.338224312
355	17	22	0.564102564	0.009862378	0.010887746	1.103967648
356	3	4	0.571428571	0.001753673	0.001675134	0.955214466
357	6	8	0.571428571	0.001736147	0.002944504	1.695999028
358	6	8	0.571428571	0.002931225	0.006232318	2.126182334
359	9	12	0.571428571	0.001820453	0.001888954	1.037628385
360	12	16	0.571428571	0.005308372	0.004825079	0.908956506
361	11	15	0.576923077	0.004187532	0.004894723	1.168880174
362	8	11	0.578947368	0.0089716	0.008554797	0.953541951
363	5	7	0.583333333	0.004830503	0.006531734	1.352184947
364	5	7	0.583333333	0.006024022	0.004548359	0.75503682
365	5	7	0.583333333	0.004873361	0.003974584	0.815573327
366	15	21	0.583333333	0.004001991	0.006198556	1.548868111
367	7	10	0.588235294	0.002876238	0.002871042	0.998193578
368	7	10	0.588235294	0.002983878	0.002756704	0.923865957
369	16	23	0.58974359	0.010437416	0.01171469	1.122374584
370	2	3	0.6	0.003151481	0.004516548	1.433151075
371	2	3	0.6	0.002844633	0.002668892	0.938220267
372	2	3	0.6	0.002142595	0.002598369	1.212720712
373	2	3	0.6	0.001818018	0.001948339	1.07168312
374	4	6	0.6	0.009554535	0.006719061	0.7032326
375	18	27	0.6	0.003671202	0.004444118	1.210534966
376	5	8	0.615384615	0.001766334	0.001750557	0.991068165
377	21	34	0.618181818	0.003165677	0.003732798	1.179146755
378	8	13	0.619047619	0.003399111	0.003210938	0.94464056
379	3	5	0.625	0.004154452	0.004735193	1.139787635
380	6	10	0.625	0.00307365	0.00341242	1.110217577
381	19	32	0.62745098	0.003511531	0.003088308	0.879476191
382	4	7	0.636363636	0.00159	0.002896275	1.821556694
383	4	7	0.636363636	0.009074559	0.009183998	1.012059996
384	5	9	0.642857143	0.005415735	0.003349153	0.61841152
385	20	36	0.642857143	0.028903299	0.029134867	1.008011835
386	6	11	0.647058824	0.002366344	0.002027857	0.856957586
387	7	13	0.65	0.002445735	0.002737803	1.119419417
388	7	13	0.65	0.005989803	0.006125443	1.022645191
389	7	13	0.65	0.006995885	0.007397848	1.05745704
390	2	4	0.666666667	0.005076906	0.004913142	0.967743248
391	2	4	0.666666667	0.002425	0.002584873	1.065926907
392	2	4	0.666666667	0.002255361	0.002606362	1.15562952
393	3	6	0.666666667	0.009582514	0.005719476	0.596865955
394	7	14	0.666666667	0.01777238	0.027783885	1.563318231
395	9	18	0.666666667	0.002077873	0.002045412	0.984377692
396	2	5	0.714285714	0.003800184	0.003710497	0.976399482
397	2	5	0.714285714	0.022951215	0.02388452	1.040664732
398	5	13	0.722222222	0.004242654	0.004150822	0.978355002
399	3	8	0.727272727	0.003424863	0.003613915	1.055199836
400	16	45	0.737704918	0.007614953	0.00775717	1.018676047
401	1	3	0.75	0.002271287	0.002310084	1.017081505
402	1	3	0.75	0.00339511	0.00258982	0.762808863
403	1	3	0.75	0.0041544	0.004541648	1.093213942
404	2	8	0.8	0.003846746	0.003648124	0.948366223
405	1	5	0.833333333	0.004327682	0.00338853	0.782989554
406	1	5	0.833333333	0.004179311	0.00423106	1.01238228
407	1	6	0.857142857	0.001862617	0.003092809	1.660464193
408	1	6	0.857142857	0.008071089	0.008228771	1.019536604
409	1	8	0.888888889	0.005553266	0.004596254	0.827666773
410	1	8	0.888888889	0.00734391	0.006028915	0.820940769
411	1	8	0.888888889	0.008681859	0.007723141	0.88957221
412	0	4	1	0	0.002497624	Inf
413	0	4	1	0	0.002797996	Inf
414	0	5	1	0	0.004035867	Inf
415	4	0	0	0.00873469	0	0
416	4	0	0	0.016684926	0	0
417	6	0	0	0.016192907	0	0
418	6	0	0	0.014991952	0	0
419	7	0	0	0.022379487	0	0
420	8	0	0	0.030934819	0	0
421	9	0	0	0.036958159	0	0
422	9	0	0	0.007662095	0	0
423	11	1	0.083333333	0.010955795	0.0092885	0.84781617
424	11	1	0.083333333	0.026788466	0.03906071	1.458116694
425	40	4	0.090909091	0.034142901	0.047075298	1.378772643
426	14	2	0.125	0.011070067	0.011177023	1.009661724
427	19	3	0.136363636	0.017455484	0.01447108	0.829027704
428	49	13	0.209677419	0.062474442	0.038809962	0.62121343
429	15	4	0.210526316	0.006603815	0.007987782	1.209570756
430	7	2	0.222222222	0.007445698	0.006883475	0.924490222
431	3	1	0.25	0.01746967	0.02341094	1.340090568
432	3	1	0.25	0.014410203	0.01664657	1.155193276
433	6	2	0.25	0.010617393	0.009434461	0.888585395
434	23	8	0.258064516	0.008531677	0.008666567	1.01581049
435	14	5	0.263157895	0.009561702	0.008681146	0.907908006
436	36	13	0.265306122	0.030852282	0.020607618	0.667944677
437	11	4	0.266666667	0.009339286	0.008055785	0.86256973
438	55	21	0.276315789	0.015257913	0.014603232	0.957092373
439	5	2	0.285714286	0.010380125	0.014595927	1.406141764
440	5	2	0.285714286	0.022735078	0.02277304	1.001669755
441	10	4	0.285714286	0.006277365	0.007204817	1.14774549
442	12	5	0.294117647	0.024009632	0.024150234	1.005856032
443	25	12	0.324324324	0.011697818	0.011037285	0.943533675
444	4	2	0.333333333	0.012845418	0.012405145	0.965725326
445	8	4	0.333333333	0.017951892	0.015722446	0.875809983
446	8	4	0.333333333	0.012385544	0.010764003	0.869077912
447	8	4	0.333333333	0.006428633	0.006190722	0.962991959
448	10	5	0.333333333	0.119921225	0.05537364	0.46175012
449	10	5	0.333333333	0.025687048	0.029196426	1.136620526
450	16	8	0.333333333	0.027389304	0.033444921	1.22109422
451	47	25	0.347222222	0.028798631	0.021464712	0.745337919
452	24	14	0.368421053	0.039282772	0.041361554	1.052918401
453	11	7	0.388888889	0.042357683	0.029159384	0.688408393
454	6	4	0.4	0.033314193	0.024690015	0.741126005
455	30	21	0.411764706	0.005941974	0.00528454	0.889357677
456	36	26	0.419354839	0.016185407	0.01680418	1.038230347
457	4	3	0.428571429	0.008815584	0.008137709	0.92310495
458	8	6	0.428571429	0.006866273	0.006321338	0.920636025
459	24	19	0.441860465	0.02580151	0.024801054	0.961224913
460	5	4	0.444444444	0.042284016	0.043886328	1.037894024
461	18	15	0.454545455	0.012193374	0.011127907	0.912619143
462	46	39	0.458823529	0.012655458	0.0110985	0.876973384
463	17	15	0.46875	0.008351104	0.006401932	0.766597098
464	19	17	0.472222222	0.031998806	0.027080962	0.846311625
465	6	6	0.5	0.008620184	0.008353288	0.969038191
466	13	13	0.5	0.021242175	0.020690278	0.974018812
467	18	18	0.5	0.024614614	0.023459767	0.953082864
468	36	37	0.506849315	0.030603943	0.022635694	0.739633249
469	9	10	0.526315789	0.016516547	0.019098491	1.156324708
470	11	13	0.541666667	0.037839335	0.034193727	0.90365561

471	22	26	0.541666667	0.01697797	0.01545116	0.910071142
472	5	6	0.545454545	0.019166357	0.013163074	0.68678015
473	7	9	0.5625	0.035337521	0.038186941	1.080634396
474	16	21	0.567567568	0.021327288	0.014796391	0.693777422
475	13	20	0.606060606	0.009448379	0.011147053	1.179784686
476	3	5	0.625	0.007632742	0.006953253	0.910977104
477	9	15	0.625	0.016700072	0.013760605	0.823984763
478	9	15	0.625	0.00900163	0.008741245	0.971073523
479	2	4	0.666666667	0.01096182	0.013652337	1.24544437
480	8	18	0.692307692	0.007501856	0.008807088	1.173987801
481	2	5	0.714285714	0.013771595	0.012001483	0.87146645
482	1	3	0.75	0.02912947	0.03233937	1.110194247
483	1	3	0.75	0.05195434	0.053309997	1.026093232
484	1	3	0.75	0.008400163	0.00668953	0.796357166
485	4	24	0.857142857	0.010519809	0.010062432	0.956522267
486	1	7	0.875	0.02664623	0.02018858	0.757652396
487	1	7	0.875	0.01335119	0.01571738	1.177226899
488	1	8	0.888888889	0.04462148	0.03085775	0.691544745
489	0	5	1	0	0.051105998	Inf
490	0	7	1	0	0.023459057	Inf
491	0	7	1	0	0.029418637	Inf
492	4	0	0	0.01255427	0	0
493	4	0	0	0.008379982	0	0
494	4	0	0	0.01219371	0	0
495	5	0	0	0.009611049	0	0
496	5	0	0	0.010568338	0	0
497	5	0	0	0.008316626	0	0
498	6	0	0	0.010313648	0	0
499	8	0	0	0.006296334	0	0
500	8	0	0	0.006331246	0	0
501	8	0	0	0.012862967	0	0
502	9	0	0	0.008459066	0	0
503	9	0	0	0.025370884	0	0
504	10	0	0	0.010277893	0	0
505	10	0	0	0.009930891	0	0
506	10	0	0	0.006024574	0	0
507	15	0	0	0.009259294	0	0
508	18	0	0	0.023339291	0	0
509	11	1	0.083333333	0.016244528	0.01147886	0.706629326
510	8	1	0.111111111	0.009524582	0.0118374	1.242826145
511	11	2	0.153846154	0.01557702	0.01690705	1.085384079
512	5	1	0.166666667	0.016591858	0.0139015	0.83785071
513	20	4	0.166666667	0.007874115	0.009205533	1.169087994
514	18	4	0.181818182	0.014206856	0.015599485	1.098025149
515	4	1	0.2	0.009776142	0.00841911	0.861189458
516	4	1	0.2	0.009804392	0.007839629	0.799603769
517	4	1	0.2	0.023898905	0.03194294	1.336585923
518	8	2	0.2	0.009939338	0.010427693	1.04913349
519	15	4	0.210526316	0.007731719	0.007969293	1.03072716
520	11	3	0.214285714	0.004806169	0.004415486	0.918712197
521	7	2	0.222222222	0.013520469	0.01226614	0.907227433
522	10	3	0.230769231	0.015411699	0.012920699	0.838369578
523	3	1	0.25	0.01669043	0.01362649	0.816425341
524	3	1	0.25	0.01099497	0.009323474	0.847976302
525	6	2	0.25	0.00563748	0.004378195	0.776622599
526	9	3	0.25	0.027963248	0.019691867	0.704205278
527	16	6	0.272727273	0.008087071	0.007390058	0.913811441
528	26	10	0.277777778	0.011062443	0.009528082	0.861299965
529	18	8	0.307692308	0.028225932	0.031169509	1.104286268
530	4	2	0.333333333	0.007559321	0.008910092	1.178689396
531	4	2	0.333333333	0.004847747	0.009858879	2.033703392
532	4	2	0.333333333	0.013495915	0.015342845	1.13685104
533	4	2	0.333333333	0.01079332	0.009909614	0.918124683
534	6	3	0.333333333	0.009146098	0.007152923	0.782073812
535	6	3	0.333333333	0.009062386	0.009407353	1.038065785
536	8	4	0.333333333	0.019195251	0.02325827	1.211667912
537	10	5	0.333333333	0.008852535	0.006915799	0.781222387
538	10	5	0.333333333	0.026937773	0.020494458	0.760807436
539	9	5	0.357142857	0.008119302	0.011548615	1.422365411
540	7	4	0.363636364	0.009051697	0.012521131	1.383290945
541	7	4	0.363636364	0.009457091	0.005392208	0.570176142
542	5	3	0.375	0.010771762	0.01575657	1.462766228
543	5	3	0.375	0.02451773	0.03661376	1.49335848
544	3	2	0.4	0.013546857	0.017088413	1.261430019
545	3	2	0.4	0.012772423	0.011438783	0.895584393
546	3	2	0.4	0.014062007	0.006318552	0.449334995
547	3	2	0.4	0.004020964	0.004121929	1.025109611
548	3	2	0.4	0.008252308	0.008046049	0.975005941
549	6	4	0.4	0.014145187	0.020270025	1.432997989
550	13	9	0.409090909	0.024391079	0.026526054	1.087530986
551	17	12	0.413793103	0.0152022	0.014506464	0.95423453
552	11	8	0.421052632	0.004961219	0.004561985	0.919529043
553	11	8	0.421052632	0.017163531	0.013034786	0.759446665
554	11	8	0.421052632	0.004093771	0.003917235	0.95687685
555	4	3	0.428571429	0.017288167	0.017906282	1.035753627
556	4	3	0.428571429	0.011337325	0.008696756	0.767090603
557	4	3	0.428571429	0.031568015	0.028709367	0.909444787
558	8	6	0.428571429	0.006362415	0.006196082	0.973857016
559	5	4	0.444444444	0.005909153	0.005136981	0.869326213
560	5	4	0.444444444	0.018823334	0.015487575	0.822785963
561	10	8	0.444444444	0.014343266	0.010168115	0.708912076
562	6	5	0.454545455	0.013600482	0.009884358	0.726765234
563	8	7	0.466666667	0.020714123	0.017745424	0.856682412
564	9	8	0.470588235	0.018902077	0.023077554	1.220900419
565	19	18	0.486486486	0.015632035	0.014903938	0.953422779
566	2	2	0.5	0.008114453	0.009634367	1.187309422
567	2	2	0.5	0.011435799	0.015692934	1.372263844
568	2	2	0.5	0.009355752	0.009054846	0.967837326
569	2	2	0.5	0.013493455	0.01609886	1.193086574
570	3	3	0.5	0.02273382	0.02240372	0.985479783
571	3	3	0.5	0.009498991	0.010172151	1.070866477
572	5	5	0.5	0.035401011	0.03983673	1.125299231
573	5	5	0.5	0.020432046	0.012694306	0.621293926
574	5	5	0.5	0.005023519	0.005388358	1.072626346
575	5	5	0.5	0.013422928	0.008949861	0.666759185
576	10	10	0.5	0.015370372	0.010550541	0.686420686
577	12	12	0.5	0.011509461	0.011791197	1.024478696
578	8	9	0.529411765	0.007806968	0.007286966	0.933392564
579	12	14	0.538461538	0.015776654	0.018364233	1.164013146
580	5	6	0.545454545	0.006934399	0.013688371	1.97398085
581	5	6	0.545454545	0.02210542	0.023446572	1.060670716
582	10	12	0.545454545	0.004786681	0.00584349	1.220781154
583	4	5	0.555555556	0.013781493	0.01622415	1.17724187
584	8	10	0.555555556	0.004966125	0.009119099	1.836260366
585	11	14	0.56	0.008521998	0.008233051	0.966094065
586	11	16	0.592592593	0.011034181	0.00842448	0.763489357
587	2	3	0.6	0.011311091	0.009325781	0.824481122
588	2	3	0.6	0.011080505	0.008497926	0.766925846
589	4	6	0.6	0.012410859	0.008702084	0.701166912
590	4	6	0.6	0.007465424	0.007473636	1.001100005
591	4	6	0.6	0.025209923	0.023372023	0.927096199
592	6	9	0.6	0.014925094	0.013886462	0.930410386
593	6	9	0.6	0.027975207	0.038510456	1.376592352

594	5	9	0.642857143	0.009435235	0.005857309	0.620791012
595	2	4	0.666666667	0.006099317	0.006588801	1.080252223
596	3	6	0.666666667	0.049258373	0.017496872	0.355206042
597	3	6	0.666666667	0.01346681	0.012186329	0.904915765
598	4	8	0.666666667	0.010720404	0.011499827	1.072704644
599	4	8	0.666666667	0.012860008	0.01263579	0.982564736
600	6	12	0.666666667	0.012596256	0.009902515	0.786147521
601	4	9	0.692307692	0.021481281	0.016500141	0.768117151
602	3	7	0.7	0.013170501	0.012556656	0.953392447
603	5	12	0.705882353	0.035741448	0.048381163	1.353643068
604	2	5	0.714285714	0.014246785	0.01023763	0.7185923
605	2	5	0.714285714	0.009148074	0.009380017	1.025354377
606	2	5	0.714285714	0.01558595	0.018055092	1.158421014
607	4	10	0.714285714	0.005772115	0.007485777	1.296886366
608	1	3	0.75	0.02774141	0.011356422	0.409367164
609	4	12	0.75	0.015203625	0.01155458	0.759988506
610	4	13	0.764705882	0.012411501	0.013199444	1.063484905
611	3	12	0.8	0.009524317	0.009922687	1.041826578
612	2	9	0.818181818	0.005899887	0.008978223	1.521761839
613	3	14	0.823529412	0.005802769	0.005896897	1.016221255
614	2	11	0.846153846	0.013612909	0.008866777	0.651350665
615	1	8	0.888888889	0.005784459	0.005152981	0.890831969
616	1	10	0.909090909	0.004932482	0.009566263	1.939441948
617	0	13	1	0	0.008192533	Inf
618	0	13	1	0	0.011005837	Inf

Suppl Fig. 3d, e	sociability (w/o opto)						sociability (w opto)					
	mouse (s)	visit to novel	time per visit (s)	empty (s)	visit to familiar	time per visit (s)	mouse (s)	visit to novel	time per visit (s)	empty (s)	visit to familiar	time per visit (s)
Unstressed dams / mCherry1	134.0416	8	16.7552	67.0208	7	9.5744	215.615	13	16.58576923	49.8651	8	6.2331375
Unstressed dams / mCherry2	240.636	8	30.0795	143.005	5	28.601	259.603	8	32.450375	99.7919	5	19.95838
Unstressed dams / mCherry3	67.1376	5	13.42752	38.01	6	6.335	86.0232	6	14.3372	41.3712	6	6.8952
Unstressed dams / mCherry4	168.691	7	24.09871429	72.5017	4	18.125425	102.672	6	17.112	52.8162	8	6.602025
Unstressed dams / mCherry5	116.513	7	16.64471429	68.4993	8	8.5624125	104.029	10	10.4029	43.3719	3	14.4573
Unstressed dams / mCherry6	227	8	28.375	50.0936	4	12.5234	177.2571	10	17.72571	60.0193	5	12.00386
Unstressed dams / mCherry7	160.203	11	14.56390909	28.3276	5	5.66552	240.154	11	21.83218182	81.6253	7	11.66075714
Unstressed dams / mCherry8	151.973	11	13.81572727	112.999	8	14.124875	101.87	10	10.187	60.9781	5	12.19562
Unstressed dams / eNPHR1	187.496	8	23.437	60.658	8	7.58225	187.816	12	15.65133333	61.1383	6	10.18971667
Unstressed dams / eNPHR2	172.7	11	15.7	26.0891	4	6.522275	186.136	14	13.29542857	25.3677	5	5.07354
Unstressed dams / eNPHR3	162.2891	7	23.18415714	90.1072	6	15.01786667	186.936	5	37.3872	77.1429	5	15.42858
Unstressed dams / eNPHR4	147.479	6	24.57983333	62.0966	6	10.34943333	205.016	7	29.288	84.8231	5	16.96462
Unstressed dams / eNPHR5	182.129	10	18.2129	37.6902	4	9.42255	134.447	9	14.93855556	21.6076	3	7.202533333
Unstressed dams / eNPHR6	209.664	11	19.06036364	56.3373	5	11.26746	238.543	10	23.8543	46.1722	6	7.695366667
Unstressed dams / eNPHR7	180.8524	11	16.44112727	111.6324	10	11.16324	267.68	15	17.84533333	74.2621	10	7.42621
Unstressed dams / eNPHR8	226.719	8	28.339875	40.094	6	6.682333333	256.877	13	19.75976923	61.8586	10	6.18586
Stressed dams / tdTomato1	116.591	6	19.43183333	44.0115	3	14.6705	153.962	6	25.66033333	15.5242	2	7.7621
Stressed dams / tdTomato2	147.801	8	18.475125	77.3016	7	11.04308571	166.533	8	20.816625	72.6632	7	10.38045714
Stressed dams / tdTomato3	168.766	12	14.06383333	58.576	5	11.7152	161.723	15	10.78153333	62.8968	7	8.985257143
Stressed dams / tdTomato4	188.6095	6	31.43491667	44.4117	6	7.40195	104.748	7	14.964	33.769	6	5.628166667
Stressed dams / tdTomato5	170.1242	5	34.02484	69.5381	5	13.90762	84.7427	5	16.94854	53.6944	4	13.4236
Stressed dams / tdTomato6	88.5837	8	11.0729625	31.6885	6	5.281416667	153.88	11	13.98909091	75.6198	8	9.452475
Stressed dams / tdTomato7	106.909	7	15.27271429	59.8565	5	11.9713	108.034	7	15.43342857	45.2944	6	7.549066667
Stressed dams / tdTomato8	229.591	4	57.39775	18.9659	3	6.321966667	114.751	5	22.9502	54.4946	4	13.62365
Stressed dams / Chrimson1	242.1472	7	34.59245714	23.2064	2	11.6032	223.341	8	27.917625	59.5364	5	11.90728
Stressed dams / Chrimson2	247.984	10	24.7984	85.7823	7	12.25461429	168.286	4	42.0715	45.0523	3	15.01743333
Stressed dams / Chrimson3	153.169	6	25.52816667	52.0967	4	13.024175	235.904	9	26.21155556	70.7391	8	8.8423875
Stressed dams / Chrimson4	265.114	6	44.18566667	46.4929	5	9.29858	223.98	7	31.99714286	30.8083	4	7.702075
Stressed dams / Chrimson5	110.751	6	18.4585	38.5707	3	12.8569	71.6985	3	23.8995	34.6489	3	11.54963333
Stressed dams / Chrimson6	84.1035	4	21.025875	43.0521	2	21.52605	121.794	6	20.299	51.0542	4	12.76355
Stressed dams / Chrimson7	204.534	8	25.56675	92.5845	5	18.5169	203.494	8	25.43675	75.3001	5	15.06002
Stressed dams / Chrimson8	100.19	11	9.108181818	67.2203	7	9.6029	204.063	7	29.15185714	49.2952	7	7.042171429

Suppl Fig. 3g		PrL	
	c-Fos$^+$	area (mm^2)	cFOS/mm2
Unstressed dam / mCherry_1	62	0.231	268.3982684
Unstressed dam / mCherry_2	84	0.254	330.7086614
Unstressed dam / mCherry_3	76	0.224	339.2857143
Unstressed dam / mCherry_4	61	0.238	256.302521
Unstressed dam / mCherry_5	71	0.283	250.8833922
Unstressed dam / mCherry_6	61	0.279	218.6379928
Unstressed dam / mCherry_7	72	0.242	297.5206612
Unstressed dam / mCherry_8	87	0.291	298.9690722
Unstressed dam / eNPHR_1	54	0.259	208.4942085
Unstressed dam / eNPHR_2	34	0.284	119.7183099
Unstressed dam / eNPHR_3	57	0.213	267.6056338
Unstressed dam / eNPHR_4	42	0.288	145.8333333
Unstressed dam / eNPHR_5	37	0.242	152.892562
Unstressed dam / eNPHR_6	45	0.227	198.2378855
Unstressed dam / eNPHR_7	62	0.291	213.0584192
Unstressed dam / eNPHR_8	28	0.266	105.2631579
Stressed dam / tdTomato_1	38	0.278	136.6906475
Stressed dam / tdTomato_2	45	0.238	189.0756303
Stressed dam / tdTomato_3	31	0.285	108.7719298
Stressed dam / tdTomato_4	49	0.253	193.6758893
Stressed dam / tdTomato_5	36	0.257	140.077821
Stressed dam / tdTomato_6	41	0.201	203.9800995
Stressed dam / tdTomato_7	37	0.203	182.2660099
Stressed dam / tdTomato_8	56	0.238	235.2941176
Stressed dam / Chrimson_1	55	0.274	200.729927
Stressed dam / Chrimson_2	73	0.207	352.6570048
Stressed dam / Chrimson_3	76	0.225	337.7777778
Stressed dam / Chrimson_4	58	0.217	267.281106
Stressed dam / Chrimson_5	61	0.251	243.0278884
Stressed dam / Chrimson_6	51	0.203	251.2315271
Stressed dam / Chrimson_7	68	0.283	240.2826855
Stressed dam / Chrimson_8	62	0.204	303.9215686

Suppl Fig. 4a-d	sociability (w/o opto)						sociability (w opto)					
	Mouse-excited	Mouse-suppressed	Mouse-unresponsible	Empty-excited	Empty-suppressed	Empty-unresponsible	Mouse-excited	Mouse-suppressed	Mouse-unresponsible	Empty-excited	Empty-suppressed	Empty-unresponsible
Unstressed dams / mCherry1	86	85	52	81	94	48	62	104	57	85	99	39
Unstressed dams / mCherry2	44	24	6	19	54	1	44	20	10	17	53	4
Unstressed dams / mCherry3	75	103	13	77	69	45	82	100	9	79	65	47
Unstressed dams / mCherry4	58	66	13	41	62	34	76	49	12	54	49	34
Unstressed dams / mCherry5	52	86	6	43	58	43	87	49	8	64	47	33
Unstressed dams / mCherry6	70	44	45	80	63	16	45	59	55	66	86	7
Unstressed dams / mCherry7	88	60	5	70	76	7	80	64	9	64	81	8
Unstressed dams / mCherry8	35	42	7	43	35	6	26	51	7	40	37	7
Unstressed dams / eNPHR1	62	50	12	39	78	7	76	43	5	34	81	9
Unstressed dams / eNPHR2	52	75	11	68	60	10	55	67	16	62	67	9
Unstressed dams / eNPHR3	79	76	9	51	77	36	68	87	9	73	51	40
Unstressed dams / eNPHR4	42	69	52	72	68	23	59	52	52	69	75	19
Unstressed dams / eNPHR5	69	42	51	62	68	32	69	42	51	51	77	34
Unstressed dams / eNPHR6	52	51	10	43	41	29	56	47	10	41	45	27
Unstressed dams / eNPHR7	77	59	10	54	86	6	51	89	6	83	53	10
Unstressed dams / eNPHR8	72	81	12	56	67	42	75	79	11	71	56	38
Stressed dams / tdTomato1	71	60	12	85	45	13	59	68	16	55	79	9
Stressed dams / tdTomato2	51	82	75	46	110	52	33	91	84	81	79	48
Stressed dams / tdTomato3	78	72	14	70	80	14	71	74	19	74	76	14
Stressed dams / tdTomato4	32	77	44	51	75	27	37	69	47	61	58	34
Stressed dams / tdTomato5	79	53	10	50	60	32	79	48	15	59	49	34
Stressed dams / tdTomato6	51	33	6	37	40	13	51	34	5	40	43	7
Stressed dams / tdTomato7	75	56	15	57	52	37	50	82	14	59	44	43
Stressed dams / tdTomato8	75	64	11	80	66	4	103	38	9	69	72	9
Stressed dams / Chrimson1	43	57	16	48	43	25	59	44	13	26	54	36
Stressed dams / Chrimson2	65	91	11	85	55	27	86	69	12	76	60	31
Stressed dams / Chrimson3	46	27	13	40	40	6	43	34	9	38	43	5
Stressed dams / Chrimson4	57	62	54	60	77	36	58	66	49	57	85	31
Stressed dams / Chrimson5	64	49	40	49	94	10	41	75	37	75	71	7
Stressed dams / Chrimson6	69	39	8	38	50	28	53	56	7	45	44	27
Stressed dams / Chrimson7	59	56	16	42	61	28	59	56	16	51	45	35
Stressed dams / Chrimson8	60	69	16	56	74	15	56	73	16	68	63	14

Suppl Fig. 5b, c	Sociability						Social novelty					
	mouse (s)	visit to mouse	stay per visit	empty (s)	visit to empty	stay per visit	novel mouse (s)	visit to novel	stay per visit	familiar mouse (s)	visit to familiar	stay per visit
Virgin female with SILA1	141.795	10	14.1795	81.8355	10	8.18355	102.578	10	10.2578	82.4358	8	10.304475
Virgin female with SILA2	109.908	8	13.7385	67.3587	8	8.4198375	126.789	11	11.52627273	97.0427	10	9.70427
Virgin female with SILA3	150.665	13	11.58961538	104.245	11	9.476818182	89.3014	14	6.378671429	89.8349	17	5.284405882
Virgin female with SILA4	181.671	16	11.3544375	100.039	10	10.0039	188.678	9	20.96422222	68.7617	9	7.640188889
Virgin female with SILA5	155.46	17	9.144705882	120.246	10	12.0246	91.9024	7	13.12891429	32.4793	7	4.6399
Virgin female with SILA6	158.861	15	10.59073333	83.3653	12	6.947108333	113.377	12	9.448083333	90.9015	14	6.492964286
Virgin female with SILA7	91.3016	6	15.21693333	86.4331	7	12.34758571	118.113	13	9.085615385	52.1538	7	7.450542857
Virgin female with SILA8	117.045	9	13.005	29.8782	6	4.9797	157.061	15	10.47073333	70.1604	10	7.01604

Suppl Fig. 5d, e	sociability						social novelty					
	Mouse-excited	Mouse-suppressed	Mouse-unresponsible	Empty-excited	Empty-suppressed	Empty-unresponsible	Familiar-excited	Familiar-suppressed	Familiar-unresponsible	Novel-excited	Novel-suppressed	Novel-unresponsible
Virgin female with SILA1	85	83	28	90	66	40	62	90	26	110	61	7
Virgin female with SILA2	43	39	23	29	64	12	71	24	13	31	62	15
Virgin female with SILA3	93	56	20	42	86	41	49	95	18	101	45	16
Virgin female with SILA4	44	50	14	48	39	21	29	65	8	20	63	19
Virgin female with SILA5	72	81	37	95	63	32	88	80	27	88	99	8
Virgin female with SILA6	40	44	21	37	37	31	59	24	22	57	31	17
Virgin female with SILA7	67	48	4	59	27	33	56	50	14	43	60	17
Virgin female with SILA8	36	46	11	34	36	23	50	46	20	53	41	22

Suppl Fig. 6b	c-Fos+Vglu1+mm2	c-Fos+Vgat+mm2
Unstressed dam_1	91.15462525	23.61111111
Unstressed dam_2	71.47814018	29.78160159
Unstressed dam_3	90.74074074	40.22988506
Unstressed dam_4	125.8928571	42.14285714
Unstressed dam_5	102.020202	28.92561983
Unstressed dam_6	151.9230769	34.35114504
Unstressed dam_7	150.877193	40.49586777
Unstressed dam_8	136.1344538	25.61983471
Stressed dam_1	49.96577687	28.96551724
Stressed dam_2	25.65445026	24.16254805
Stressed dam_3	93.87755102	21.41680395
Stressed dam_4	65.04854369	42.14876033
Stressed dam_5	76.99115044	56.58914729
Stressed dam_6	49.61832061	34.7107438
Stressed dam_7	71.90082645	48.51485149
Stressed dam_8	63.80952381	49.5412844

Suppl Fig. 6d	Vglut1+/EGFP+_Labeled cell (%)	Vgat+/EGFP+_Labeled cell (%)
male_1	84.83290488	13.75358166
male_2	72.36467236	10.4859335
male_3	71.03174603	15.59633028
male_4	77.21774194	23.3490566
male_5	78.47358121	16.03053435
male_6	58.40266223	16.21129326
female_1	86.86514886	16.66666667
female_2	64.70588235	13.5451505
female_3	83.22033898	11.51241535
female_4	79.29606625	21.19700748
female_5	79.77272727	15.06024096
female_6	87.67123288	19.37377691

Suppl Fig. 6g, h	Sociability (w/o opto)						Sociability (w opto)					
	mouse (s)	visit to mouse	stay per visit	empty (s)	visit to empty	stay per visit	mouse (s)	visit to mouse	stay per visit	empty (s)	visit to empty	stay per visit
BLA-PrL/Unstressed dams / eNPHR1	149.662	10	14.9662	37.3153	4	9.328825	164.121	12	13.67675	56.2735	5	11.2547
BLA-PrL/Unstressed dams / eNPHR2	191.732	13	14.74861538	37.8502	8	4.731275	124.028	11	11.27527273	62.8968	6	10.4828
BLA-PrL/Unstressed dams / eNPHR3	183.005	10	18.3005	45.3511	4	11.337775	114.3079	8	14.2884875	66.6958	10	6.66958
BLA-PrL/Unstressed dams / eNPHR4	94.5028	7	13.5004	60.4898	6	10.08163333	171.125	12	14.26041667	28.8778	3	9.625933333
BLA-PrL/Unstressed dams / eNPHR5	168.446	12	14.03716667	62.5772	8	7.82215	136.381	10	13.6381	56.3566	9	6.261844444
BLA-PrL/Unstressed dams / eNPHR6	154.994	15	10.33293333	85.4999	10	8.54999	151.184	11	13.744	73.5617	6	12.26028333
BLA-PrL/Unstressed dams / eNPHR7	158.002	10	15.8002	59.7593	6	9.959883333	196.601	11	17.87281818	61.6905	11	5.608227273
BLA-PrL/Unstressed dams / eNPHR8	67.2302	7	9.604314286	60.5605	8	7.5700625	83.3008	8	10.4126	76.7648	7	10.9664
BLA-PrL/Stressed dams / Chrimson1	202.505	10	20.2505	26.3011	3	8.767033333	134.242	8	16.78025	71.0928	6	11.8488
BLA-PrL/Stressed dams / Chrimson2	147.299	8	18.412375	58.8769	8	7.3596125	192.975	12	16.08125	105.799	9	11.75544444
BLA-PrL/Stressed dams / Chrimson3	141.399	12	11.78325	20.7266	3	6.908866667	106.994	8	13.37425	27.8489	3	9.282966667
BLA-PrL/Stressed dams / Chrimson4	135.712	8	16.964	114.672	7	16.38171429	189.331	11	17.21190909	93.9451	6	15.65751667
BLA-PrL/Stressed dams / Chrimson5	142.182	10	14.2182	41.9499	5	8.38998	144.93	6	24.155	46.0201	3	15.34003333
BLA-PrL/Stressed dams / Chrimson6	173.395	15	11.55966667	61.7583	11	5.614390909	158.932	13	12.22553846	73.4968	9	8.166311111
BLA-PrL/Stressed dams / Chrimson7	145.184	13	11.168	73.4966	9	8.166288889	159.131	12	13.26091667	60.0244	7	8.574914286
BLA-PrL/Stressed dams / Chrimson8	107.514	5	21.5028	55.6057	4	13.901425	140.649	9	15.62766667	59.4103	7	8.487185714

Suppl Fig. 6g, h	Social novelty (w/o opto)						Social novelty (w opto)					
	novel mouse (s)	visit to novel	stay per visit	familiar mouse (s)	visit to familiar	stay per visit	novel mouse (s)	visit to novel	stay per visit	familiar mouse (s)	visit to familiar	stay per visit
BLA-PrL/Unstressed dams / eNPHR1	136.486	13	10.49892308	80.8391	10	8.08391	167.392	10	16.7392	116.819	14	8.344214286
BLA-PrL/Unstressed dams / eNPHR2	107.7035	7	15.38621429	64.1772	5	12.83544	123.068	8	15.3835	67.5381	9	7.504233333
BLA-PrL/Unstressed dams / eNPHR3	133.383	8	16.672875	95.6415	9	10.62683333	164.455	10	16.4455	58.6224	9	6.5136
BLA-PrL/Unstressed dams / eNPHR4	94.3618	5	18.87236	32.0123	3	10.67076667	149.256	7	21.32228571	58.8922	6	9.815366667
BLA-PrL/Unstressed dams / eNPHR5	133.719	12	11.14325	98.3117	14	7.022264286	106.2392	5	21.24784	40.285	7	5.755
BLA-PrL/Unstressed dams / eNPHR6	157.374	15	10.4916	63.8244	12	5.3187	167.723	16	10.4826875	69.8934	9	7.765933333
BLA-PrL/Unstressed dams / eNPHR7	173.859	10	17.3859	65.9588	13	5.073753846	126.315	13	9.716538462	102.572	10	10.2572
BLA-PrL/Unstressed dams / eNPHR8	138.656	15	9.243733333	75.4302	13	5.802323077	156.404	17	9.200235294	82.3041	14	5.878864286
BLA-PrL/Stressed dams / Chrimson1	67.0876	8	8.38595	96.9265	12	8.077208333	101.065	10	10.1065	133.241	15	8.882733333
BLA-PrL/Stressed dams / Chrimson2	112.213	11	10.20118182	116.152	13	8.934769231	110.277	12	9.18975	109.61	10	10.961
BLA-PrL/Stressed dams / Chrimson3	75.7841	10	7.57841	61.1394	6	10.1899	106.193	6	17.69883333	95.1492	9	10.57213333
BLA-PrL/Stressed dams / Chrimson4	70.7425	8	8.8428125	40.0127	6	6.668783333	113.39	10	11.339	128.914	7	18.41628571
BLA-PrL/Stressed dams / Chrimson5	125.314	11	11.39218182	88.1001	15	5.87334	129.857	10	12.9857	70.6309	12	5.885908333
BLA-PrL/Stressed dams / Chrimson6	125.717	15	8.381133333	140.19	13	10.78384615	131.588	15	8.772533333	103.509	10	10.3509
BLA-PrL/Stressed dams / Chrimson7	72.7629	9	8.084766667	137.723	17	8.101352941	66.7607	6	11.12678333	94.7055	9	10.52283333
BLA-PrL/Stressed dams / Chrimson8	97.1932	10	9.71932	96.3922	7	13.77031429	71.2263	7	10.17518571	57.6753	7	8.239328571

Suppl Fig. 6i

	sociability (w/o opto)						sociability (w opto)					
	Mouse-excited	Mouse-suppressed	Mouse-unresponsible	Empty-excited	Empty-suppressed	Empty-unresponsible	Mouse-excited	Mouse-suppressed	Mouse-unresponsible	Empty-excited	Empty-suppressed	Empty-unresponsible
BLA-PrL/Unstressed dams / eNPHR1	20	23	3	18	23	5	19	21	6	22	19	5
BLA-PrL/Unstressed dams / eNPHR2	59	70	33	85	47	30	54	80	28	52	75	35
BLA-PrL/Unstressed dams / eNPHR3	49	67	23	63	66	10	59	49	31	69	53	17
BLA-PrL/Unstressed dams / eNPHR4	37	50	6	29	53	11	60	27	6	33	58	2
BLA-PrL/Unstressed dams / eNPHR5	65	61	29	71	61	23	69	50	36	55	71	29
BLA-PrL/Unstressed dams / eNPHR6	76	55	26	63	62	32	57	74	26	67	63	27
BLA-PrL/Unstressed dams / eNPHR7	70	63	28	58	69	34	42	91	28	74	56	31
BLA-PrL/Unstressed dams / eNPHR8	63	39	17	57	50	12	63	40	16	78	32	9
BLA-PrL/Stressed dams / Chrimson1	35	50	3	52	36	0	22	59	7	64	22	2
BLA-PrL/Stressed dams / Chrimson2	65	81	11	68	76	13	70	54	33	76	69	12
BLA-PrL/Stressed dams / Chrimson3	67	44	37	64	61	23	66	73	9	55	62	31
BLA-PrL/Stressed dams / Chrimson4	75	52	28	73	65	17	66	55	34	88	50	17
BLA-PrL/Stressed dams / Chrimson5	54	73	32	50	82	27	52	73	34	59	65	35
BLA-PrL/Stressed dams / Chrimson6	80	61	59	76	89	35	80	82	38	77	95	28
BLA-PrL/Stressed dams / Chrimson7	62	78	11	56	62	33	55	82	14	52	63	36
BLA-PrL/Stressed dams / Chrimson8	53	89	17	55	63	41	53	75	31	63	64	32

Suppl Fig. 6j	social novelty (w/o opto)						social novelty (w opto)					
	Familiar-excited	Familiar-suppressed	Familiar-unresponsible	Novel-excited	Novel-suppressed	Novel-unresponsible	Familiar-excited	Familiar-suppressed	Familiar-unresponsible	Novel-excited	Novel-suppressed	Novel-unresponsible
BLA-PrL/Unstressed dams / eNPHR1	23	31	9	27	28	8	9	47	7	26	31	6
BLA-PrL/Unstressed dams / eNPHR2	57	75	11	98	28	17	57	73	13	79	49	15
BLA-PrL/Unstressed dams / eNPHR3	66	86	20	98	58	16	87	63	22	95	49	28
BLA-PrL/Unstressed dams / eNPHR4	32	40	11	24	42	17	43	34	6	33	37	13
BLA-PrL/Unstressed dams / eNPHR5	58	73	19	60	66	24	63	70	17	62	59	29
BLA-PrL/Unstressed dams / eNPHR6	62	90	14	82	66	18	61	91	14	82	66	18
BLA-PrL/Unstressed dams / eNPHR7	68	68	14	71	61	18	48	86	16	88	50	12
BLA-PrL/Unstressed dams / eNPHR8	54	86	15	61	62	32	54	76	25	51	78	26
BLA-PrL/Stressed dams / Chrimson1	37	42	7	43	35	8	20	63	3	50	32	4
BLA-PrL/Stressed dams / Chrimson2	50	84	19	67	69	17	61	58	34	71	71	11
BLA-PrL/Stressed dams / Chrimson3	57	75	22	67	76	11	63	78	13	58	85	11
BLA-PrL/Stressed dams / Chrimson4	60	59	20	41	84	14	60	65	14	66	58	15
BLA-PrL/Stressed dams / Chrimson5	80	63	20	59	87	17	67	82	14	55	84	24
BLA-PrL/Stressed dams / Chrimson6	57	80	11	79	58	11	82	55	11	65	67	16
BLA-PrL/Stressed dams / Chrimson7	72	74	14	52	81	27	64	86	10	57	92	11
BLA-PrL/Stressed dams / Chrimson8	67	64	18	67	72	10	65	71	13	54	80	15

Suppl Fig. 8a, b	Neuronal activity during interaction with mouse cage (off->on)									Neuronal activity during interaction with empty cage (off->on)								
	unresponsible->unresponsible	unresponsible->excited	unresponsible->suppressed	excited->unresponsible	excited->excited	excited->suppressed	suppressed->unresponsible	suppressed->excited	suppressed->suppressed	unresponsible->unresponsible	unresponsible->excited	unresponsible->suppressed	excited->unresponsible	excited->excited	excited->suppressed	suppressed->unresponsible	suppressed->excited	suppressed->suppressed
Unstressed dams / mCherry1	41	2	9	11	32	43	5	28	52	30	8	10	5	37	39	4	40	50
Unstressed dams / mCherry2	1	4	1	6	30	8	3	10	11	0	0	1	1	9	9	3	8	43
Unstressed dams / mCherry3	2	3	8	3	43	29	4	36	63	33	9	3	7	44	26	7	26	36
Unstressed dams / mCherry4	2	4	7	4	44	10	6	28	32	21	4	9	4	18	19	9	32	21
Unstressed dams / mCherry5	0	0	6	2	43	7	6	44	36	21	14	8	5	22	16	7	28	23
Unstressed dams / mCherry6	40	2	3	9	37	24	6	6	32	1	4	11	4	38	38	2	24	37
Unstressed dams / mCherry7	0	3	2	6	59	23	3	18	39	0	7	0	4	27	39	4	30	42
Unstressed dams / mCherry8	1	1	5	1	23	11	5	2	35	0	3	3	4	26	13	3	11	21
Unstressed dams / eNPHR1	1	5	6	1	48	13	3	23	24	0	4	5	1	15	18	6	20	55
Unstressed dams / eNPHR2	0	6	5	5	29	18	11	20	44	0	7	3	4	37	27	5	18	37
Unstressed dams / eNPHR3	2	3	4	4	44	31	3	21	52	30	5	1	6	26	19	4	42	31
Unstressed dams / eNPHR4	40	8	4	6	20	16	6	31	32	3	13	7	8	32	32	8	24	36
Unstressed dams / eNPHR5	43	4	4	8	45	16	7	18	17	20	7	5	6	28	28	8	16	44
Unstressed dams / eNPHR6	1	6	3	3	37	12	6	13	32	20	5	4	5	21	17	2	15	24
Unstressed dams / eNPHR7	0	4	6	3	34	40	3	13	43	0	3	3	2	37	15	8	43	35
Unstressed dams / eNPHR8	1	5	6	7	49	16	3	21	57	30	10	2	3	30	23	5	31	31
Stressed dams / tdTomato1	2	5	5	9	40	22	5	14	41	0	5	8	7	36	42	2	14	29
Stressed dams / tdTomato2	43	5	27	9	22	20	32	6	44	31	13	8	5	17	24	12	51	47
Stressed dams / tdTomato3	1	7	6	10	35	33	8	29	35	0	6	8	5	33	32	9	35	36
Stressed dams / tdTomato4	41	0	3	1	13	18	5	24	48	22	2	3	7	22	22	5	37	33
Stressed dams / tdTomato5	1	5	4	6	55	18	8	19	26	20	5	7	7	23	20	7	31	22
Stressed dams / tdTomato6	1	1	4	2	39	10	2	11	20	1	6	6	3	22	12	3	12	25
Stressed dams / tdTomato7	0	5	10	9	34	32	5	11	40	30	5	2	6	28	23	7	26	19
Stressed dams / tdTomato8	0	8	3	6	56	13	3	39	22	0	2	2	6	40	34	3	27	36
Stressed dams / Chrimson1	5	6	5	4	29	10	4	24	29	21	1	3	9	19	20	6	6	31
Stressed dams / Chrimson2	0	6	5	3	45	17	9	35	47	21	3	3	4	49	32	6	24	25
Stressed dams / Chrimson3	3	5	5	4	29	13	2	9	16	0	4	2	4	24	12	1	10	29
Stressed dams / Chrimson4	21	8	25	11	40	6	17	10	35	21	10	5	6	20	34	4	27	46
Stressed dams / Chrimson5	20	2	18	5	22	37	12	17	20	0	8	2	2	20	27	5	47	42
Stressed dams / Chrimson6	1	3	4	3	39	27	3	11	25	20	3	5	4	17	17	3	25	22
Stressed dams / Chrimson7	4	7	5	6	31	22	6	21	29	22	3	3	7	17	18	6	31	24
Stressed dams / Chrimson8	3	7	6	4	30	26	9	19	41	0	10	5	7	29	20	7	29	38

Suppl Fig. 9b-e

	sociability (w/o opto)						sociability (with opto)					
	mouse (s)	visit to mouse	time per visit	empty (s)	visit to empty	time per visit	mouse (s)	visit to mouse	time per visit	empty (s)	visit to empty	time per visit
virgin-male_AI-PrL_mCherry_1	142.388	12	11.86566667	65.6251	9	7.291677778	154.581	13	11.89084615	65.5544	8	8.1943
virgin-male_AI-PrL_mCherry_2	167.894	8	20.98675	102.113	9	11.34588889	169.322	12	14.11016667	52.8931	6	8.815516667
virgin-male_AI-PrL_mCherry_3	117.871	14	8.419357143	15.4441	3	5.148033333	121.9446	9	13.5494	24.0037	3	8.001233333
virgin-male_AI-PrL_mCherry_4	104.268	10	10.4268	24.8867	3	8.295566667	125.3055	10	12.53055	65.5349	7	9.362128571
virgin-male_AI-PrL_mCherry_5	113.577	18	6.309833333	35.9471	9	3.994122222	128.1706	14	9.155042857	95.8993	14	6.84995
virgin-male_AI-PrL_mCherry_6	133.158	8	16.64475	44.4125	6	7.402083333	97.9608	8	12.2451	40.3456	5	8.06912
virgin-male_AI-PrL_mCherry_7	159.21	19	8.379473684	84.1615	16	5.26009375	156.39	14	11.17071429	119.983	12	9.998583333
virgin-male_AI-PrL_mCherry_8	114.232	6	19.03866667	78.9633	8	9.8704125	156.516	16	9.78225	72.0908	12	6.007566667
virgin-male_AI-PrL_eNPHR3_1	161.162	12	13.43016667	45.5318	7	6.504542857	81.8627	8	10.2328375	50.494	7	7.213428571
virgin-male_AI-PrL_eNPHR3_2	108.51	10	10.851	70.8193	8	8.8524125	136.044	12	11.337	69.0621	10	6.90621
virgin-male_AI-PrL_eNPHR3_3	124.584	9	13.84266667	73.897	11	6.717909091	211.164	19	11.11389474	60.828	10	6.0828
virgin-male_AI-PrL_eNPHR3_4	182.475	17	10.73382353	51.3544	8	6.4193	190.077	15	12.6718	62.1584	11	5.650763636
virgin-male_AI-PrL_eNPHR3_5	147.127	7	21.01814286	56.4233	7	8.060471429	132.473	13	10.19023077	52.7799	7	7.539985714
virgin-male_AI-PrL_eNPHR3_6	133.455	11	12.13227273	73.2302	8	9.153775	176.541	19	9.291631579	42.6178	6	7.102966667
virgin-male_AI-PrL_eNPHR3_7	121.446	14	8.674714286	88.834	10	8.8834	85.5666	9	9.5074	40.4824	5	8.09648
virgin-male_AI-PrL_eNPHR3_8	178.803	21	8.514428571	89.5684	12	7.464033333	156.194	15	10.41293333	45.8177	9	5.090855556
virgin-female_AI-PrL_mCherry_1	112.31	13	8.639230769	82.5654	11	7.505945455	149.792	13	11.52246154	78.2343	14	5.588164286
virgin-female_AI-PrL_mCherry_2	165.942	17	9.761294118	87.0393	11	7.912663636	98.845	10	9.8845	51.8281	9	5.758677778
virgin-female_AI-PrL_mCherry_3	97.0401	8	12.1300125	58.224	7	8.317714286	150.527	14	10.75192857	95.576	9	10.61955556
virgin-female_AI-PrL_mCherry_4	140.46	19	7.392631579	47.3534	8	5.919175	96.4387	8	12.0548375	65.8971	6	10.98285
virgin-female_AI-PrL_mCherry_5	125.723	16	7.8576875	92.3748	13	7.105753846	117.586	18	6.532555556	78.5067	15	5.23378
virgin-female_AI-PrL_mCherry_6	202.348	22	9.197636364	50.3537	8	6.2942125	172.469	20	8.62345	73.1667	10	7.31667
virgin-female_AI-PrL_mCherry_7	170.601	19	8.979	89.8357	13	6.910438462	147.125	18	8.173611111	114.049	15	7.603266667
virgin-female_AI-PrL_mCherry_8	103.4292	12	8.6191	87.3681	10	8.73681	115.58	15	7.705333333	53.2255	11	4.838681818
virgin-female_AI-PrL_eNPHR3_1	78.0927	13	6.007130769	44.6838	6	7.4473	96.6373	12	8.053108333	86.7001	11	7.881827273
virgin-female_AI-PrL_eNPHR3_2	151.861	15	10.12406667	94.1044	11	8.554945455	157.404	16	9.83775	94.1092	10	9.41092
virgin-female_AI-PrL_eNPHR3_3	179.128	13	13.77907692	75.7623	9	8.418033333	167.866	16	10.491625	57.4892	13	4.422246154
virgin-female_AI-PrL_eNPHR3_4	42.7422	3	14.2474	40.7492	6	6.791533333	105.24	9	11.69333333	69.7598	10	6.97598
virgin-female_AI-PrL_eNPHR3_5	195.543	23	8.501869565	74.8992	12	6.2416	131.318	12	10.94316667	63.7583	11	5.796209091
virgin-female_AI-PrL_eNPHR3_6	184.065	14	13.1475	132.586	13	10.19892308	137.919	14	9.851357143	48.7519	9	5.416877778
virgin-female_AI-PrL_eNPHR3_7	149.917	19	7.890368421	120.046	16	7.502875	166.138	16	10.383625	97.5087	11	8.864427273
virgin-female_AI-PrL_eNPHR3_8	158.054	13	12.158	104.641	10	10.4641	137.386	13	10.56815385	27.0103	5	5.40206

	social novelty (w/o opto)						social novelty (with opto)					
	novel mouse (s)	visit to novel	time per visit	familiar mouse (s)	visit to familiar	time per visit	novel mouse (s)	visit to novel	time per visit	familiar mouse (s)	visit to familiar	time per visit
virgin-male_AI-PrL_mCherry_1	146.645	10	14.6645	85.8334	12	7.152783333	121.7018	15	8.113453333	109.575	11	9.961363636
virgin-male_AI-PrL_mCherry_2	195.485	10	19.5485	102.988	13	7.922153846	137.954	10	13.7954	80.5056	10	8.05056
virgin-male_AI-PrL_mCherry_3	171.399	8	21.424875	90.5049	10	9.05049	176.92	10	17.692	42.1714	7	6.024485714
virgin-male_AI-PrL_mCherry_4	54.4868	5	10.89736	52.8947	5	10.57894	146.591	12	12.21591667	69.8587	8	8.7323375
virgin-male_AI-PrL_mCherry_5	132.573	12	11.04775	90.3682	16	5.6480125	114.6324	10	11.46324	68.4262	12	5.702183333
virgin-male_AI-PrL_mCherry_6	124.429	11	11.31172727	47.8625	5	9.5725	146.714	5	29.3428	45.2184	6	7.5364
virgin-male_AI-PrL_mCherry_7	123.522	15	8.2348	83.5711	11	7.597372727	165.188	17	9.716941176	81.0325	11	7.366590909
virgin-male_AI-PrL_mCherry_8	120.919	9	13.43544444	73.0317	11	6.639245455	136.853	9	15.20588889	66.4925	8	8.3115625
virgin-male_AI-PrL_eNPHR3_1	119.552	13	9.196307692	76.3404	12	6.3617	55.3749	11	5.034081818	70.0189	8	8.7523625
virgin-male_AI-PrL_eNPHR3_2	216.149	8	27.018625	94.1099	8	11.7637375	105.313	14	7.522357143	84.4268	13	6.494369231
virgin-male_AI-PrL_eNPHR3_3	132.521	13	10.19392308	82.1003	14	5.864307143	87.6126	14	6.258042857	102.76	10	10.276
virgin-male_AI-PrL_eNPHR3_4	146.016	10	14.6016	77.6482	13	5.972938462	91.642	9	10.18244444	104.915	13	8.070384615
virgin-male_AI-PrL_eNPHR3_5	164.868	13	12.68215385	79.2325	8	9.9040625	113.446	10	11.3446	105.377	13	8.105923077
virgin-male_AI-PrL_eNPHR3_6	106.243	6	17.70716667	105.91	6	17.65166667	98.2411	12	8.186758333	57.6241	10	5.76241
virgin-male_AI-PrL_eNPHR3_7	148.323	12	12.36025	111.043	18	6.169055556	137.919	16	8.6199375	131.917	16	8.2448125
virgin-male_AI-PrL_eNPHR3_8	118.313	18	6.572944444	50.4863	13	3.883561538	124.515	14	8.893928571	138.587	15	9.239133333
virgin-female_AI-PrL_mCherry_1	184.213	19	9.695421053	79.101	13	6.084692308	147.795	13	11.36884615	44.7519	7	6.393128571
virgin-female_AI-PrL_mCherry_2	127.519	7	18.217	56.2229	8	7.0278625	131.659	7	18.80842857	52.6903	7	7.527185714
virgin-female_AI-PrL_mCherry_3	138.19	11	12.56272727	74.5639	9	8.284877778	192.554	14	13.75385714	93.8424	9	10.42693333
virgin-female_AI-PrL_mCherry_4	116.38	10	11.638	48.4861	7	6.926585714	156.063	9	17.34033333	63.0255	8	7.8781875
virgin-female_AI-PrL_mCherry_5	176.137	13	13.549	88.702	12	7.391833333	169.803	15	11.3202	88.5032	13	6.807938462
virgin-female_AI-PrL_mCherry_6	170.143	19	8.954894737	86.1053	14	6.150378571	75.2971	8	9.4121375	126.118	20	6.3059
virgin-female_AI-PrL_mCherry_7	154.528	11	14.048	92.3701	13	7.105392308	168.938	20	8.4469	94.3734	17	5.551376471
virgin-female_AI-PrL_mCherry_8	107.649	12	8.97075	104.647	15	6.976466667	141.39	13	10.87615385	51.3538	11	4.668527273
virgin-female_AI-PrL_eNPHR3_1	140.187	9	15.57633333	39.2817	5	7.85634	110.443	7	15.77757143	69.6271	6	11.60451667
virgin-female_AI-PrL_eNPHR3_2	155.536	11	14.13963636	104.714	14	7.479571429	116.713	13	8.977923077	63.0253	13	4.8481
virgin-female_AI-PrL_eNPHR3_3	165.605	12	13.80041667	75.1659	9	8.351766667	92.041	11	8.367363636	91.3074	11	8.300672727
virgin-female_AI-PrL_eNPHR3_4	109.6228	6	18.27046667	34.1464	5	6.82928	71.2277	5	14.24554	44.5507	4	11.137675
virgin-female_AI-PrL_eNPHR3_5	162.664	13	12.51261538	95.371	13	7.336230769	113.243	14	8.088785714	147.523	16	9.2201875
virgin-female_AI-PrL_eNPHR3_6	174.535	12	14.54458333	129.05	17	7.591176471	108.975	11	9.906818182	139.12	15	9.274666667
virgin-female_AI-PrL_eNPHR3_7	164.997	15	10.9998	64.3582	12	5.363183333	92.8403	10	9.28403	81.7688	9	9.085422222
virgin-female_AI-PrL_eNPHR3_8	135.186	10	13.5186	91.769	14	6.554928571	129.856	12	10.82133333	108.981	12	9.08175

Suppl Fig. 10a, b	S-off						S-on					
	mouse positive	mouse negative	mouse NS	empty positive	empty negative	empty NS	mouse positive	mouse negative	mouse NS	empty positive	empty negative	empty NS
virgin-male_AI-PrL_mCherry_1	79	69	20	70	86	12	75	74	19	86	69	13
virgin-male_AI-PrL_mCherry_2	73	58	18	56	65	28	76	56	17	51	69	29
virgin-male_AI-PrL_mCherry_3	70	50	23	57	56	30	67	54	22	58	59	26
virgin-male_AI-PrL_mCherry_4	26	33	6	26	30	9	32	27	6	28	30	7
virgin-male_AI-PrL_mCherry_5	36	87	28	69	57	25	62	63	26	52	78	21
virgin-male_AI-PrL_mCherry_6	87	55	13	70	57	28	60	82	13	75	65	15
virgin-male_AI-PrL_mCherry_7	72	73	12	54	86	17	75	69	13	47	85	25
virgin-male_AI-PrL_mCherry_8	20	43	9	34	33	5	37	31	4	36	34	2
virgin-male_AI-PrL_eNPHR3_1	72	55	21	50	73	25	62	65	21	52	65	31
virgin-male_AI-PrL_eNPHR3_2	71	67	12	85	55	10	68	64	18	70	72	8
virgin-male_AI-PrL_eNPHR3_3	67	49	11	59	37	31	39	73	15	73	26	28
virgin-male_AI-PrL_eNPHR3_4	27	64	8	56	34	9	58	34	7	42	53	4
virgin-male_AI-PrL_eNPHR3_5	56	81	13	78	65	7	64	71	15	57	79	14
virgin-male_AI-PrL_eNPHR3_6	42	11	8	11	43	7	31	21	9	28	30	3
virgin-male_AI-PrL_eNPHR3_7	75	63	37	51	97	27	76	72	27	51	89	35
virgin-male_AI-PrL_eNPHR3_8	64	61	17	62	58	22	67	57	18	57	65	20
virgin-female_AI-PrL_mCherry_1	52	40	10	25	72	5	50	42	10	36	14	52
virgin-female_AI-PrL_mCherry_2	100	49	15	79	50	35	95	50	19	69	67	28
virgin-female_AI-PrL_mCherry_3	45	72	20	54	49	34	43	73	21	74	39	24
virgin-female_AI-PrL_mCherry_4	107	41	12	61	73	26	86	40	34	71	62	27
virgin-female_AI-PrL_mCherry_5	64	83	22	72	58	39	60	81	28	80	54	35
virgin-female_AI-PrL_mCherry_6	39	46	14	41	54	4	44	47	8	48	44	7
virgin-female_AI-PrL_mCherry_7	58	39	71	49	53	66	58	60	50	28	122	18
virgin-female_AI-PrL_mCherry_8	28	48	13	34	41	14	26	50	13	45	33	11
virgin-female_AI-PrL_eNPHR3_1	60	50	30	60	49	31	50	57	33	51	67	22
virgin-female_AI-PrL_eNPHR3_2	61	33	14	40	58	10	58	34	16	49	51	8
virgin-female_AI-PrL_eNPHR3_3	60	45	17	56	50	16	38	60	24	40	62	20
virgin-female_AI-PrL_eNPHR3_4	63	49	28	50	58	32	53	64	23	69	44	27
virgin-female_AI-PrL_eNPHR3_5	68	55	14	60	69	8	66	68	3	64	61	12
virgin-female_AI-PrL_eNPHR3_6	64	78	18	53	83	24	62	75	23	64	73	23
virgin-female_AI-PrL_eNPHR3_7	50	78	19	60	68	19	61	72	14	60	68	19
virgin-female_AI-PrL_eNPHR3_8	76	73	10	56	63	40	90	51	18	69	71	19

Suppl Fig. 10c, d			SN-off						SN-on			
	familiar positive	familiar negative	familiar NS	novel positive	novel negative	novel NS	familiar positive	familiar negative	familiar NS	novel positive	novel negative	novel NS
virgin-male_AI-PrL_mCherry_1	64	65	24	65	66	22	70	67	16	70	73	10
virgin-male_AI-PrL_mCherry_2	58	60	13	64	59	8	65	53	13	70	40	21
virgin-male_AI-PrL_mCherry_3	55	52	8	58	52	5	43	62	10	46	51	18
virgin-male_AI-PrL_mCherry_4	46	58	14	60	50	8	49	62	7	53	51	14
virgin-male_AI-PrL_mCherry_5	71	63	25	69	65	25	59	65	35	52	90	17
virgin-male_AI-PrL_mCherry_6	67	82	14	65	73	25	71	78	14	88	53	22
virgin-male_AI-PrL_mCherry_7	72	66	36	86	66	22	66	84	24	81	62	31
virgin-male_AI-PrL_mCherry_8	78	57	19	78	62	14	81	56	17	79	64	11
virgin-male_AI-PrL_eNPHR3_1	64	71	22	85	52	20	53	82	22	82	58	17
virgin-male_AI-PrL_eNPHR3_2	50	63	13	64	42	20	52	57	17	54	56	16
virgin-male_AI-PrL_eNPHR3_3	57	54	21	31	86	15	39	77	16	41	70	21
virgin-male_AI-PrL_eNPHR3_4	59	65	18	67	65	10	65	62	15	53	74	15
virgin-male_AI-PrL_eNPHR3_5	84	61	14	86	52	21	67	71	21	58	96	5
virgin-male_AI-PrL_eNPHR3_6	22	34	6	25	23	14	20	30	12	25	24	13
virgin-male_AI-PrL_eNPHR3_7	74	66	24	83	59	22	71	61	32	71	72	21
virgin-male_AI-PrL_eNPHR3_8	69	63	21	84	59	10	67	65	21	53	84	16
virgin-female_AI-PrL_mCherry_1	31	46	10	27	46	14	33	42	12	39	34	14
virgin-female_AI-PrL_mCherry_2	56	47	11	74	25	15	49	43	22	64	37	13
virgin-female_AI-PrL_mCherry_3	87	62	24	87	69	17	60	89	24	76	67	30
virgin-female_AI-PrL_mCherry_4	51	68	22	78	54	9	75	38	28	94	34	13
virgin-female_AI-PrL_mCherry_5	59	70	16	67	67	11	60	64	21	56	68	21
virgin-female_AI-PrL_mCherry_6	46	45	11	36	57	9	58	31	13	43	48	11
virgin-female_AI-PrL_mCherry_7	56	79	23	58	66	34	54	82	22	60	77	21
virgin-female_AI-PrL_mCherry_8	76	63	17	72	69	15	54	80	22	57	73	26
virgin-female_AI-PrL_eNPHR3_1	74	67	14	78	52	25	78	59	18	54	87	14
virgin-female_AI-PrL_eNPHR3_2	48	42	7	29	57	11	38	41	18	27	52	18
virgin-female_AI-PrL_eNPHR3_3	58	58	24	60	64	16	54	69	17	46	69	25
virgin-female_AI-PrL_eNPHR3_4	66	42	27	62	53	20	51	67	17	47	68	20
virgin-female_AI-PrL_eNPHR3_5	49	71	19	65	54	20	42	80	17	49	79	11
virgin-female_AI-PrL_eNPHR3_6	52	80	14	68	59	19	49	78	19	79	49	18
virgin-female_AI-PrL_eNPHR3_7	67	69	19	72	68	15	69	70	16	52	84	19
virgin-female_AI-PrL_eNPHR3_8	42	41	17	51	38	11	49	34	17	35	47	18

Suppl Fig. 11	Neuronal activity during interaction with novel mice (off->on)								
	unresponsible->unresponsible	unresponsible->excited	unresponsible->suppressed	excited->unresponsible	excited->excited	excited->suppressed	suppressed->unresponsible	suppressed->excited	suppressed->suppressed
virgin-male_AI-PrL_mCherry_1	10	12	0	0	58	7	0	0	66
virgin-male_AI-PrL_mCherry_2	0	3	5	9	43	12	12	24	23
virgin-male_AI-PrL_mCherry_3	2	2	1	9	27	22	7	17	28
virgin-male_AI-PrL_mCherry_4	2	3	3	7	30	23	5	20	25
virgin-male_AI-PrL_mCherry_5	3	3	19	8	42	19	6	7	52
virgin-male_AI-PrL_mCherry_6	2	8	15	8	39	18	12	41	20
virgin-male_AI-PrL_mCherry_7	4	8	10	16	53	17	11	20	35
virgin-male_AI-PrL_mCherry_8	1	7	6	5	44	29	5	28	29
virgin-male_AI-PrL_eNPHR3_1	1	13	6	8	38	24	8	31	28
virgin-male_AI-PrL_eNPHR3_2	0	9	11	8	26	30	8	19	15
virgin-male_AI-PrL_eNPHR3_3	5	6	4	4	16	11	12	19	55
virgin-male_AI-PrL_eNPHR3_4	3	3	4	4	38	25	8	12	45
virgin-male_AI-PrL_eNPHR3_5	1	9	11	3	37	46	1	12	39
virgin-male_AI-PrL_eNPHR3_6	4	3	7	3	17	5	6	5	12
virgin-male_AI-PrL_eNPHR3_7	4	8	10	7	33	43	10	30	19
virgin-male_AI-PrL_eNPHR3_8	0	3	7	9	40	35	7	10	42
virgin-female_AI-PrL_mCherry_1	2	7	5	4	12	11	8	20	18
virgin-female_AI-PrL_mCherry_2	3	4	8	7	51	16	3	9	13
virgin-female_AI-PrL_mCherry_3	4	3	10	13	52	22	13	21	35
virgin-female_AI-PrL_mCherry_4	2	2	5	5	62	11	6	30	18
virgin-female_AI-PrL_mCherry_5	2	5	4	10	32	25	9	19	39
virgin-female_AI-PrL_mCherry_6	0	7	2	5	20	11	6	16	35
virgin-female_AI-PrL_mCherry_7	8	5	21	3	29	26	10	26	30
virgin-female_AI-PrL_mCherry_8	3	7	5	14	33	25	9	17	43
virgin-female_AI-PrL_eNPHR3_1	4	5	16	5	36	37	5	13	34
virgin-female_AI-PrL_eNPHR3_2	1	3	7	3	9	17	14	15	28
virgin-female_AI-PrL_eNPHR3_3	4	1	11	12	18	30	9	27	28
virgin-female_AI-PrL_eNPHR3_4	1	10	9	8	21	33	11	16	26
virgin-female_AI-PrL_eNPHR3_5	3	7	10	3	30	32	5	10	37
virgin-female_AI-PrL_eNPHR3_6	1	11	7	11	36	21	6	32	21
virgin-female_AI-PrL_eNPHR3_7	4	2	9	7	38	27	8	22	38
virgin-female_AI-PrL_eNPHR3_8	2	3	6	8	27	16	8	5	25

Suppl Fig. 12b	novel object recognition (without opto)		novel object recognition (with opto)	
	familiar object (s)	novel object (s)	familiar object (s)	novel object (s)
Unstressed dams / mCherry1	29.32815	72.86	48.0133	71.22
Unstressed dams / mCherry2	33.48945	57.016	39.9311	36.21005
Unstressed dams / mCherry3	26.8874	70.8195	29.96825	43.77205
Unstressed dams / mCherry4	31.40865	60.1765	21.6859	57.1355
Unstressed dams / mCherry5	74.3805	62.0285	19.5654	67.9785
Unstressed dams / mCherry6	51.014	64.018	41.8115	64.858
Unstressed dams / mCherry7	45.9346	51.6565	16.64515	67.381
Unstressed dams / mCherry8	23.72635	76.3005	43.97175	57.6955
Unstressed dams / eNPHR1	41.61145	35.84985	45.05225	41.73135
Unstressed dams / eNPHR2	40.17075	71.259	31.6887	32.16885
Unstressed dams / eNPHR3	34.84965	66.2985	59.219	82.2265
Unstressed dams / eNPHR4	24.76685	59.0965	44.53215	81.9425
Unstressed dams / eNPHR5	36.80985	55.735	29.6481	66.258
Unstressed dams / eNPHR6	25.92695	45.6122	34.651	52.0165
Unstressed dams / eNPHR7	37.69025	72.0995	37.6901	69.6185
Unstressed dams / eNPHR8	29.88815	44.132	32.8891	48.41345
Stressed dams / tdTomato1	43.1874	61.694	46.0006	87.6795
Stressed dams / tdTomato2	63.2605	78.462	66.018	62.633
Stressed dams / tdTomato3	47.1326	50.7735	27.0073	52.8545
Stressed dams / tdTomato4	24.808	37.292	50.3281	73.98
Stressed dams / tdTomato5	31.2086	39.931	19.7656	61.2975
Stressed dams / tdTomato6	16.60425	66.697	16.4044	51.3335
Stressed dams / tdTomato7	30.52855	54.9755	26.24735	35.77
Stressed dams / tdTomato8	21.5258	43.61175	18.2049	55.655
Stressed dams / Chrimson1	22.3614	42.4199	42.5219	54.1425
Stressed dams / Chrimson2	50.4325	71.3595	53.5365	63.5815
Stressed dams / Chrimson3	30.10585	35.31265	27.26895	46.16025
Stressed dams / Chrimson4	33.17655	36.9815	28.7708	57.2745
Stressed dams / Chrimson5	28.9682	66.2985	53.0145	91.065
Stressed dams / Chrimson6	24.4985	76.633	40.0501	49.89575
Stressed dams / Chrimson7	44.12415	44.1575	53.87	67.6215
Stressed dams / Chrimson8	21.19325	34.77695	44.7584	43.6236

Suppl Fig. 12c, d	Light off						Light on					
	Familiar object-excited	Familiar object-suppressed	Familiar object-unresponsible	Novel object-excited	Novel object-suppressed	Novel object-unresponsible	Familiar object-excited	Familiar object-suppressed	Familiar object-unresponsible	Novel object-excited	Novel object-suppressed	Novel object-unresponsible
Unstressed dams / mCherry1	49	44	12	41	57	7	44	50	11	33	60	12
Unstressed dams / mCherry2	74	54	20	49	72	26	70	62	16	39	89	20
Unstressed dams / mCherry3	79	78	26	63	88	32	86	69	28	65	94	24
Unstressed dams / mCherry4	31	55	20	42	45	20	40	52	14	22	63	21
Unstressed dams / mCherry5	45	43	17	47	45	13	24	60	21	47	48	10
Unstressed dams / mCherry6	35	73	13	51	53	17	52	56	13	66	34	21
Unstressed dams / mCherry7	55	29	10	39	42	13	44	33	17	41	41	12
Unstressed dams / mCherry8	49	52	13	8	94	12	62	32	20	39	63	12
Unstressed dams / eNPHR1	37	32	13	51	18	13	47	19	16	45	25	12
Unstressed dams / eNPHR2	59	54	7	49	60	11	37	66	17	48	59	13
Unstressed dams / eNPHR3	41	56	15	29	71	12	45	52	15	53	42	17
Unstressed dams / eNPHR4	75	82	30	58	100	29	80	85	22	66	95	26
Unstressed dams / eNPHR5	39	45	22	38	52	16	34	64	8	43	50	13
Unstressed dams / eNPHR6	47	77	22	43	81	22	54	71	21	45	82	19
Unstressed dams / eNPHR7	103	46	12	53	95	13	75	55	31	75	58	28
Unstressed dams / eNPHR8	38	52	9	50	35	14	38	50	11	20	66	13
Stressed dams / tdTomato1	35	11	7	19	26	8	27	22	4	19	20	14
Stressed dams / tdTomato2	91	56	22	61	87	21	68	60	41	59	82	28
Stressed dams / tdTomato3	41	92	21	65	69	20	74	67	13	35	100	19
Stressed dams / tdTomato4	55	65	20	45	79	16	48	80	12	79	31	30
Stressed dams / tdTomato5	39	55	7	32	60	9	37	55	9	34	57	10
Stressed dams / tdTomato6	55	53	17	56	58	11	62	54	9	54	60	11
Stressed dams / tdTomato7	53	56	13	56	47	19	60	53	9	48	65	9
Stressed dams / tdTomato8	71	51	21	52	71	20	66	55	22	63	63	17
Stressed dams / Chrimson1	66	50	8	59	50	15	60	45	19	37	60	27
Stressed dams / Chrimson2	55	34	6	41	43	11	54	32	9	34	49	12
Stressed dams / Chrimson3	65	72	13	53	74	23	73	65	12	52	80	18
Stressed dams / Chrimson4	37	48	13	44	50	4	60	26	12	31	49	18
Stressed dams / Chrimson5	59	72	22	45	89	19	47	86	20	53	82	18
Stressed dams / Chrimson6	38	55	7	38	48	14	39	50	11	44	47	9
Stressed dams / Chrimson7	55	63	25	59	63	21	59	73	11	67	69	7
Stressed dams / Chrimson8	65	73	29	59	91	17	73	77	17	98	55	14

Suppl Fig. 12f	familiar object (s)	visit to familiar object	time per visit	novel object (s)	visit to novel object	time per visit
Unstressed dam_1	36.8141	12	3.067841667	66.511	19	3.500578947
Unstressed dam_2	25.3707	11	2.306427273	56.9833	16	3.56145625
Unstressed dam_3	41.6202	22	1.891827273	72.03	21	3.43
Unstressed dam_4	50.03	19	2.633157895	81.7847	13	6.291130769
Unstressed dam_5	25.8464	7	3.692342857	65.146	16	4.071625
Unstressed dam_6	29.6074	15	1.973826667	49.787	25	1.99148
Unstressed dam_7	40.0928	16	2.5058	54.5041	19	2.868636842
Unstressed dam_8	30.8105	8	3.8513125	50.506	14	3.607571429
Stressed dam_1	29.7733	12	2.481108333	82.924	21	3.948761905
Stressed dam_2	19.8507	5	3.97014	39.9011	10	3.99011
Stressed dam_3	23.8533	12	1.987775	42.6609	14	3.047207143
Stressed dam_4	37.6167	16	2.35104375	53.106	16	3.319125
Stressed dam_5	30.6612	18	1.7034	68.441	23	2.975695652
Stressed dam_6	35.8598	16	2.2412375	60.436	23	2.627652174
Stressed dam_7	21.7756	18	1.209755556	73.637	22	3.347136364
Stressed dam_8	45.5391	21	2.168528571	79.16	28	2.827142857

Suppl Fig. 12h, i			PPD7						PPD13			
	familiar object (s)	visit to familiar object	time per visit	novel object (s)	visit to novel object	time per visit	familiar object (s)	visit to familiar object	time per visit	novel object (s)	visit to novel object	time per visit
Unstressed dam_1	31.2529	11	2.841172727	81.162	19	4.271684211	37.3746	14	2.669614286	74.885	18	4.160277778
Unstressed dam_2	18.9694	12	1.580783333	48.4243	17	2.848488235	45.7833	13	3.521792308	65.96	18	3.664444444
Unstressed dam_3	41.5372	18	2.307622222	73.031	19	3.843736842	33.4557	13	2.573515385	68.914	16	4.307125
Unstressed dam_4	41.6211	18	2.312283333	55.508	14	3.964857143	24.1734	14	1.726671429	58.189	23	2.529956522
Unstressed dam_5	42.4851	10	4.24851	56.5034	17	3.323729412	25.5317	11	2.321063636	60.281	18	3.348944444
Unstressed dam_6	25.333	12	2.111083333	71.636	18	3.979777778	26.8121	10	2.68121	71.23	18	3.957222222
Unstressed dam_7	23.2099	12	1.934158333	73.15	17	4.302941176	17.1688	10	1.71688	66.036	15	4.4024
Unstressed dam_8	39.2965	14	2.806892857	55.2633	16	3.45395625	35.2504	10	3.52504	53.72	18	2.984444444
Stressed dam_1	38.1756	18	2.120866667	66.199	23	2.878217391	36.4219	18	2.023438889	70.196	18	3.899777778
Stressed dam_2	48.2595	15	3.2173	81.111	17	4.771235294	31.739	13	2.441461538	61.471	16	3.8419375
Stressed dam_3	37.9792	15	2.531946667	68.58	26	2.637692308	36.3384	12	3.0282	89.236	15	5.949066667
Stressed dam_4	42.2217	13	3.247823077	56.409	17	3.318176471	31.0561	15	2.070406667	53.7814	12	4.481783333
Stressed dam_5	28.0942	11	2.554018182	49.7057	13	3.823515385	33.8205	11	3.074590909	47.313	11	4.301181818
Stressed dam_6	31.2964	12	2.608033333	73.838	18	4.102111111	24.0948	9	2.6772	64.918	14	4.637
Stressed dam_7	42.6177	19	2.243036842	65.717	18	3.650944444	21.3304	6	3.555066667	65.5444	12	5.462033333
Stressed dam_8	32.4939	14	2.320992857	73.351	21	3.492904762	35.3333	11	3.212118182	75.234	17	4.425529412

Suppl Fig. 12k	Stimulated side (sec)	Unstimulated side (sec)
Closed-loop (group-eNPHR3) 10	870.72	919.84
closed-loop (group-eNPHR3) 11	924.48	865.52
closed-loop (group-eNPHR3) 12	778.535	1002.759
closed-loop (group-eNPHR3) 13	849.182	949.526
closed-loop (group-eNPHR3) 14	830.05	968.806
closed-loop (group-eNPHR3) 15	839.419	952.089
closed-loop (group-eNPHR3) 16	1043.226	690.816
closed-loop (group-eNPHR3) 17	766.68	1033.467
closed-loop (isolation-chrimson) 10	990.038	787.097
closed-loop (isolation-chrimson) 11	893.047	893.929
closed-loop (isolation-chrimson) 12	795.023	996.357
closed-loop (isolation-chrimson) 13	937.459	859.595
closed-loop (isolation-chrimson) 14	998.018	800.687
closed-loop (isolation-chrimson) 15	902.634	894.236
closed-loop (isolation-chrimson) 16	792.449	1006.905
closed-loop (isolation-chrimson) 17	885.686	909.69

Suppl Fig. 13b, c		sociability (without opto)						sociability (with opto)					
		mouse (s)	visit to mouse	time per visit (s)	empty (s)	visit to empty	time per visit (s)	mouse (s)	visit to mouse	time per visit (s)	empty (s)	visit to empty	time per visit (s)
closed-loop_Unstressed dams / eNPHR2	exploration	116.912	10	11.6912	95.7058	9	10.63397778	107.389	13	8.260692308	74.18	10	7.418
closed-loop_Unstressed dams / eNPHR4	exploration	97.9459	5	19.58918	36.2496	2	18.1248	209.107	10	20.9107	58.7388	5	11.74776
closed-loop_Unstressed dams / eNPHR6	exploration	106.989	4	26.74725	56.9752	4	14.2438	160.764	8	20.0955	52.8945	8	6.6118125
closed-loop_Unstressed dams / eNPHR8	exploration	128.275	11	11.66136364	44.2519	9	4.916877778	141.878	12	11.82316667	64.4973	8	8.0621625
closed-loop_Unstressed dams / eNPHR10	exploration	199.015	17	11.70676471	55.6153	11	5.055936364	142.599	16	8.9124375	40.491	10	4.0491
closed-loop_Unstressed dams / eNPHR12	exploration	114.351	8	14.293875	58.2559	7	8.322271429	84.1027	6	14.01711667	37.05	4	9.2625
closed-loop_Unstressed dams / eNPHR14	exploration	154.281	16	9.6425625	50.2534	6	8.375566667	164.764	11	14.97854545	55.8549	6	9.30915
closed-loop_Unstressed dams / eNPHR16	exploration	172.605	20	8.63025	37.93	7	5.418571429	112.19	11	10.19909091	41.5311	7	5.933014286
closed-loop_Unstressed dams / eNPHR1	interaction	287.775	9	31.975	136.778	6	22.79633333	229.091	15	15.27273333	61.011	11	5.546454545
closed-loop_Unstressed dams / eNPHR3	interaction	135.637	13	10.43361538	106.669	10	10.6669	141.477	11	12.86154545	92.0239	10	9.20239
closed-loop_Unstressed dams / eNPHR5	interaction	77.8651	5	15.57302	54.4976	4	13.6244	108.829	8	13.603625	75.5404	7	10.79148571
closed-loop_Unstressed dams / eNPHR7	interaction	120.192	16	7.512	81.5418	14	5.824414286	173.328	14	12.38057143	78.1017	11	7.100154545
closed-loop_Unstressed dams / eNPHR9	interaction	108.349	14	7.739214286	38.6503	6	6.441716667	105.228	13	8.094461538	93.1449	13	7.164992308
closed-loop_Unstressed dams / eNPHR11	interaction	126.193	13	9.707153846	46.8122	7	6.687457143	74.1006	8	9.262575	56.2556	6	9.375933333
closed-loop_Unstressed dams / eNPHR13	interaction	190.372	8	23.7965	72.0198	6	12.0033	144.359	13	11.10453846	51.7741	5	10.35482
closed-loop_Unstressed dams / eNPHR15	interaction	169.086	9	18.78733333	59.6161	7	8.516585714	181.168	19	9.535157895	49.6133	10	4.96133
closed-loop_Stressed dams / Chrimson3	exploration	129.955	6	21.65916667	35.3695	3	11.78983333	148.04	9	16.44888889	58.3356	9	6.481733333
closed-loop_Stressed dams / Chrimson4	exploration	82.3739	5	16.47478	59.4775	6	9.912916667	116.819	6	19.46983333	23.2303	4	5.807575
closed-loop_Stressed dams / Chrimson6	exploration	167.406	18	9.300333333	86.1039	10	8.61039	177.159	19	9.324157895	69.6887	13	5.360669231
closed-loop_Stressed dams / Chrimson8	exploration	94.8254	6	15.80423333	49.1331	5	9.82662	152.12	12	12.67666667	44.9718	4	11.24295
closed-loop_Stressed dams / Chrimson7	exploration	270.953	11	24.63209091	47.9958	7	6.856542857	172.291	9	19.14344444	53.0691	7	7.5813
closed-loop_Stressed dams / Chrimson10	exploration	130.836	9	14.53733333	53.4547	6	8.909116667	184.85	10	18.485	76.5006	8	9.562575
closed-loop_Stressed dams / Chrimson12	exploration	146.047	14	10.43192857	72.5832	9	8.0648	102.269	10	10.2269	59.5367	8	7.4420875
closed-loop_Stressed dams / Chrimson14	exploration	145.806	10	14.5806	53.697	5	10.7394	93.7053	8	11.7131625	90.1843	9	10.02047778
closed-loop_Stressed dams / Chrimson1	interaction	150.93	15	10.062	71.5599	11	6.505445455	191.582	14	13.68442857	66.9535	7	9.564785714
closed-loop_Stressed dams / Chrimson2	interaction	112.013	7	16.00185714	77.9684	7	11.13834286	156.062	7	22.29457143	75.3612	6	12.5602
closed-loop_Stressed dams / Chrimson5	interaction	123.428	10	12.3428	77.8349	8	9.7293625	127.899	9	14.211	96.2581	6	16.04301667
closed-loop_Stressed dams / Chrimson9	interaction	128.681	11	11.69827273	53.7772	5	10.75544	144.686	9	16.07622222	44.4142	5	8.88284
closed-loop_Stressed dams / Chrimson11	interaction	108.11	5	21.622	74.8206	5	14.96412	209.099	9	23.23322222	52.8148	3	17.60493333
closed-loop_Stressed dams / Chrimson13	interaction	186.94	12	15.57833333	55.6978	9	6.188644444	133.877	12	11.15641667	64.3378	10	6.43378
closed-loop_Stressed dams / Chrimson15	interaction	207.095	9	23.01055556	61.0563	6	10.17605	131.802	10	13.1802	74.904	11	6.809454545
closed-loop_Stressed dams / Chrimson16	interaction	212.536	10	21.2536	34.4891	5	6.89782	170.845	10	17.0845	53.7741	5	10.75482

Suppl Fig. 13d, e		sociability (w/o opto)						sociability (w opto)					
		Mouse-excited	Mouse-suppressed	Mouse-unresponsible	Empty-excited	Empty-suppressed	Empty-unresponsible	Mouse-excited	Mouse-suppressed	Mouse-unresponsible	Empty-excited	Empty-suppressed	Empty-unresponsible
closed-loop_Unstressed dams / eNPHR2	exploration	59	35	8	47	45	10	45	50	7	48	43	11
closed-loop_Unstressed dams / eNPHR4	exploration	78	50	17	50	80	15	81	44	20	65	61	19
closed-loop_Unstressed dams / eNPHR6	exploration	87	69	39	79	87	29	78	86	31	87	78	30
closed-loop_Unstressed dams / eNPHR8	exploration	29	52	21	45	48	9	32	43	27	38	45	19
closed-loop_Unstressed dams / eNPHR10	exploration	32	47	23	40	49	13	36	56	10	46	39	17
closed-loop_Unstressed dams / eNPHR12	exploration	56	61	7	54	46	24	75	40	9	69	39	16
closed-loop_Unstressed dams / eNPHR14	exploration	46	38	8	37	39	16	47	40	5	33	42	17
closed-loop_Unstressed dams / eNPHR16	exploration	33	69	14	43	48	25	26	78	12	20	82	14
closed-loop_Unstressed dams / eNPHR1	interaction	34	35	15	28	51	5	29	35	20	40	34	10
closed-loop_Unstressed dams / eNPHR3	interaction	44	58	20	50	43	29	54	37	31	47	48	27
closed-loop_Unstressed dams / eNPHR5	interaction	69	52	18	65	56	18	68	44	27	54	66	19
closed-loop_Unstressed dams / eNPHR7	interaction	79	79	36	78	89	27	100	72	22	97	81	16
closed-loop_Unstressed dams / eNPHR9	interaction	41	51	11	32	49	22	39	54	10	42	37	24
closed-loop_Unstressed dams / eNPHR11	interaction	65	67	22	51	70	33	55	76	23	43	84	27
closed-loop_Unstressed dams / eNPHR13	interaction	81	69	15	81	72	12	74	77	14	97	57	11
closed-loop_Unstressed dams / eNPHR15	interaction	58	47	10	57	46	12	42	64	9	43	65	7
closed-loop_Stressed dams / Chrimson3	exploration	20	35	5	23	35	2	24	33	3	29	22	9
closed-loop_Stressed dams / Chrimson4	exploration	49	79	31	77	44	38	71	52	36	53	71	35
closed-loop_Stressed dams / Chrimson6	exploration	74	51	40	44	100	21	74	58	33	77	55	33
closed-loop_Stressed dams / Chrimson7	exploration	59	47	21	55	54	18	67	38	22	47	62	18
closed-loop_Stressed dams / Chrimson8	exploration	53	39	11	36	43	24	45	51	7	39	45	19
closed-loop_Stressed dams / Chrimson10	exploration	56	51	10	54	43	20	48	57	12	41	61	15
closed-loop_Stressed dams / Chrimson12	exploration	44	39	21	47	38	19	41	44	19	29	57	18
closed-loop_Stressed dams / Chrimson14	exploration	63	70	13	69	55	22	71	62	13	63	50	33
closed-loop_Stressed dams / Chrimson1	interaction	37	51	25	53	42	18	48	43	22	46	45	22
closed-loop_Stressed dams / Chrimson2	interaction	47	29	4	42	25	13	47	30	3	32	32	16
closed-loop_Stressed dams / Chrimson5	interaction	78	50	25	62	53	38	72	53	28	62	69	22
closed-loop_Stressed dams / Chrimson9	interaction	22	50	17	29	33	27	20	49	20	43	28	18
closed-loop_Stressed dams / Chrimson11	interaction	85	48	20	66	63	24	69	60	24	60	71	22
closed-loop_Stressed dams / Chrimson13	interaction	43	38	21	35	59	8	57	27	18	37	51	14
closed-loop_Stressed dams / Chrimson15	interaction	74	51	25	59	71	20	67	56	27	50	79	21
closed-loop_Stressed dams / Chrimson16	interaction	56	89	22	71	81	15	63	84	20	83	62	22

Suppl Fig. 13g

Neuronal activity during interaction with novel mice (off->on)

		unresponsible->unresponsible	unresponsible->excited	unresponsible->suppressed	excited->unresponsible	excited->excited	excited->suppressed	suppressed->unresponsible	suppressed->excited	suppressed->suppressed
closed-loop_Unstressed dams / eNPHR1	interaction	2	7	0	1	18	15	6	20	13
closed-loop_Unstressed dams / eNPHR3	interaction	2	7	7	6	27	20	8	18	22
closed-loop_Unstressed dams / eNPHR5	interaction	1	13	15	10	28	29	10	24	26
closed-loop_Unstressed dams / eNPHR7	interaction	4	12	10	14	32	36	12	43	34
closed-loop_Unstressed dams / eNPHR9	interaction	0	4	3	3	18	13	3	14	43
closed-loop_Unstressed dams / eNPHR11	interaction	2	12	7	7	35	22	6	19	40
closed-loop_Unstressed dams / eNPHR13	interaction	2	13	15	8	64	22	7	14	48
closed-loop_Unstressed dams / eNPHR15	interaction	2	2	4	6	38	21	1	12	29
closed-loop_Stressed dams / Chrimson1	interaction	2	4	7	11	25	24	5	10	37
closed-loop_Stressed dams / Chrimson2	interaction	1	5	5	5	18	14	6	13	21
closed-loop_Stressed dams / Chrimson5	interaction	1	8	7	11	46	9	4	45	19
closed-loop_Stressed dams / Chrimson9	interaction	0	2	2	4	16	7	3	10	15
closed-loop_Stressed dams / Chrimson11	interaction	3	5	6	6	32	6	12	45	38
closed-loop_Stressed dams / Chrimson13	interaction	1	8	7	6	30	12	3	15	21
closed-loop_Stressed dams / Chrimson15	interaction	2	7	7	11	38	15	8	33	32
closed-loop_Stressed dams / Chrimson16	interaction	3	4	9	3	23	43	5	40	37

Suppl Fig. 14b

	AI	area (mm2)	GR/mm2	PrL	area (mm2)	GR/mm2
Unstressed dam_1	1438	2.618	549.2742552	1008	2.727	369.6369637
Unstressed dam_2	1374	2.723	504.5905252	1051	2.669	393.7804421
Unstressed dam_3	1141	2.968	384.4339623	971	2.776	349.7838617
Unstressed dam_4	1574	2.864	549.5810056	1021	2.719	375.5057006
Unstressed dam_5	1055	2.577	409.3907645	821	2.499	328.5314126
Unstressed dam_6	1432	2.978	480.8596373	901	2.901	310.5825577
			479.6883583			354.6368231
Stressed dam_1	1443	2.656	543.2981928	1192	2.512	474.522293
Stressed dam_2	1492	2.703	551.9792823	1001	2.811	356.1010317
Stressed dam_3	1501	2.815	533.2149201	996	2.824	352.6912181
Stressed dam_4	1043	2.988	349.0629183	1043	2.615	398.8527725
Stressed dam_5	1326	2.707	489.8411526	799	2.871	278.3002438
Stressed dam_6	1211	2.761	438.6091996	901	2.882	312.630118
			484.3342776			362.1829462

Suppl Fig. 15c	tdt positive	GFAP/tdt positive	%
BL6_ CRE DOG 1	181	169	93.37016575
BL6_ CRE DOG 2	131	127	96.94656489
BL6_ CRE DOG 3	191	186	97.38219895
	503	482	95.8250497

Suppl Fig. 15f	tdt positive	GFAP/tdt positive	%
Ai14_ CRE DOG 1	672	648	96.42857
Ai14_ CRE DOG 2	544	532	97.79412
Ai14_ CRE DOG 3	791	775	97.97724
	2007	1955	97.40907

Suppl Fig. 16c	mCherry positive	mCherry/GR positive	%
group/GR_mCherry/CRE-DOG 1	114	109	0.956140351
group/GR_mCherry/CRE-DOG 2	98	88	0.897959184
group/GR_mCherry/CRE-DOG 3	121	110	0.909090909
group/GR_mCherry/CRE-DOG 4	109	99	0.908256881
group/GR_mCherry/CRE-DOG 5	142	129	0.908450704
group/GR_mCherry/CRE-DOG 6	74	70	0.945945946
